# Single-cell multi-omics elucidates the role of RPS27-RPS24 fusion gene in osteosarcoma chemoresistance and metabolic regulation

**DOI:** 10.1038/s41420-025-02487-9

**Published:** 2025-04-25

**Authors:** Zhiwei Tao, Pingan Zou, Zhengxu Yang, Tao Xiong, Zhi Deng, Qincan Chen

**Affiliations:** Bone and Soft Tissue Sarcoma Department, Jiangxi Cancer Hospital, 330029 Nanchang, P.R. China

**Keywords:** Cancer, Molecular biology

## Abstract

Osteosarcoma (OS) presents significant treatment challenges due to chemoresistance. This study explores the molecular mechanisms underlying chemoresistance in OS, focusing on the novel fusion gene RPS27-RPS24. Using single-cell multi-omics techniques, we identified a significant upregulation of RPS27-RPS24 in chemoresistant OS cells. Our analyses revealed that RPS27-RPS24 enhances glutaminase (GLS)-mediated glutamine metabolism and inhibits copper-induced cell death, thereby promoting chemoresistance. In vitro experiments with adriamycin-resistant (ADMR) OS cells confirmed that overexpression of RPS27-RPS24 leads to increased cell viability and proliferation under chemotherapy. In vivo studies further validated these findings, demonstrating that targeting glutamine metabolism can reverse chemoresistance. Our results suggest that the RPS27-RPS24 fusion gene plays a critical role in OS chemoresistance through metabolic reprogramming, providing a potential therapeutic target for improving OS treatment outcomes.

**Figure:**
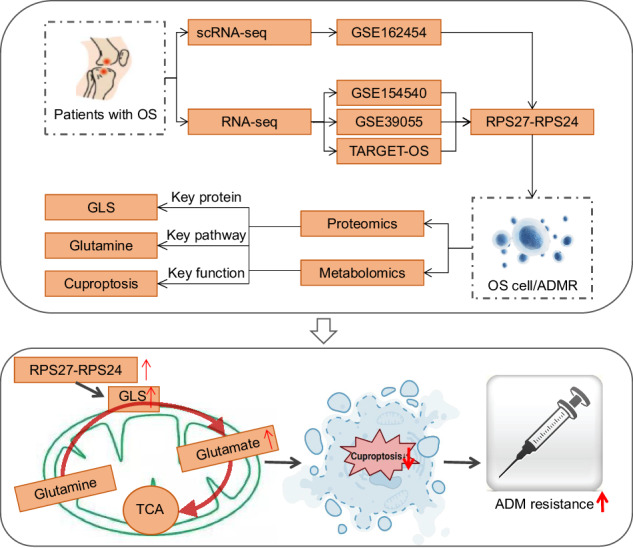
The application of multiple analytical techniques in this study (as shown in the upper image) and the hypothesized mechanism (as shown in the lower image).

The application of multiple analytical techniques in this study (as shown in the upper image) and the hypothesized mechanism (as shown in the lower image).

## Introduction

Osteosarcoma (OS) is a highly lethal malignant tumor with chemoresistance, presenting significant challenges in its treatment [1, 2]. The development of chemoresistance in the treatment of OS is increasingly evident, yet its mechanisms remain unclear (CITE). Previous studies have indicated the crucial role of fusion genes in tumor biology [3–5]. Consequently, investigating the function and mechanisms of fusion genes in OS is of great significance in improving its therapeutic efficacy [6–8].

Fusion genes are formed by fusion events of two distinct genes and have diverse origins and important functions [9]. In tumorigenesis and progression, fusion genes play a vital role in cell cycle regulation, transcription regulation, and epigenetic regulation [10, 11]. In OS, several fusion genes associated with occurrence and development have been identified, such as BCR-ABL, EWS-FLI1, etc. [3, 12, 13]. Although fusion genes have been extensively studied, their specific role in the mechanism of chemoresistance in OS remains unclear [3, 12, 13].

Single-cell multi-omics techniques, such as single-cell RNA sequencing (scRNA-seq) and proteomics and metabolomics analysis, can reveal the molecular mechanisms of complex diseases, including the formation of chemoresistance [14, 15]. The application of these techniques can overcome the limitations of traditional research and provide new perspectives for a deeper understanding of the mechanisms underlying chemoresistance in OS [16, 17]. By utilizing these techniques, we can obtain information on gene expression, protein, and metabolite levels in individual cells, thus elucidating the role of fusion genes in chemoresistance in OS.

This study employed scRNA-seq technology, along with data analysis and cell annotation methods, to screen the malignant cell markers in tumor tissues of OS patients. Key fusion genes associated with chemoresistance were identified through gene co-expression network analysis and prediction of fusion genes using online databases. Additionally, functional validation was performed using in vitro cell experiments and CRISPR/Cas9 editing technology to investigate the impact of key genes, glutamine metabolism, and copper death on chemoresistance in OS cells. To validate the experimental results, a subcutaneous xenograft mouse model of OS was constructed for in vivo experiments.

The aim of this study was to provide a comprehensive understanding of the molecular mechanisms underlying chemoresistance in OS, particularly those related to the fusion gene RPS27-RPS24. Uncovering these mechanisms is crucial for understanding chemoresistance in OS, developing new therapeutic strategies, and improving patient prognosis. By delving into the functions and regulatory mechanisms of the fusion gene RPS27-RPS24, we hope to identify new targets and approaches that can offer more effective strategies and tools for personalized treatment and precision medicine. Despite some limitations, such as limited sample size and the absence of an animal model, we believe these findings will provide new directions and insights for future OS research and translational studies in other tumors.

## Results

### Characterization of cellular heterogeneity and malignancy markers in OS

OS is a highly aggressive and metastatic malignant tumor originating from bone or soft tissues, posing a longstanding challenge in cancer treatment. This disease has a higher incidence rate in children and adolescents, causing significant physiological and psychological stress to patients and their families. Therefore, understanding the mechanisms underlying the development of OS is of critical importance for saving patients’ lives [18, 19]. Cellular changes in the tumor microenvironment play a vital role in the malignant progression of OS [20, 21].

**Fig. 1:**
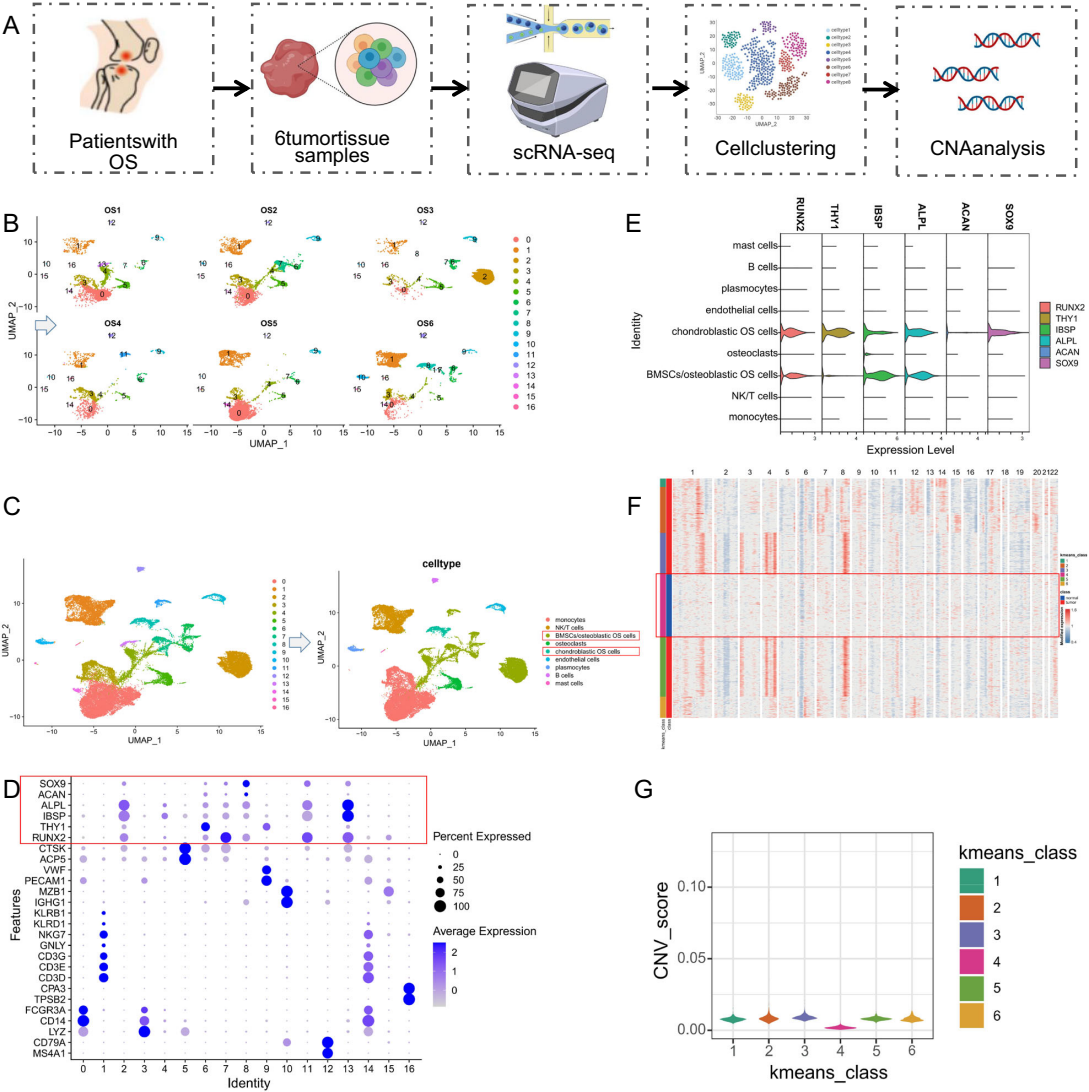
UMAP clustering, cell annotation, and CNV analysis of scRNA-seq data. **A** Workflow for scRNA-seq data analysis. **B** UMAP clustering analysis classified cells into 17 cell clusters (shown by sample origin). **C** 17 cell clusters annotated as 9 cell types, with suspected OS cells highlighted in the red box. **D** Bubble plot of marker genes for the 17 cell clusters, with suspected OS cell marker genes highlighted in the red box. **E** Violin plot showing expression of suspected OS cell marker genes across the 9 cell types. **F** CNV analysis of scRNA-seq data, with blue indicating DNA copy loss, red indicating DNA copy gain, and normal cell samples highlighted in the red box. **G** Comparison of CNV scores after *K*-means clustering.

To gain a deeper understanding of the development process of OS and changes in the tumor microenvironment, we obtained OS-related scRNA-seq data from the GEO database, specifically the GSE162454 dataset. This dataset consisted of tumor tissue samples from six OS patients, and the data analysis workflow is summarized in Fig. 1A. Firstly, we used the Seurat package to integrate the sequencing data and examine the gene counts (nFeature_RNA), the number of mRNA molecules (nCount_RNA), and the percentage of mitochondrial genes (percent.mt) in all cells of the scRNA-seq data (Fig. S1A). A quality control step was performed by filtering the data based on the criteria of 200 < nFeature_RNA < 5000, 200 < nCount_RNA < 20,000, and percent.mt < 25, resulting in a total of 37,432 cells. The calculation of sequencing depth correlation revealed a correlation coefficient of *r* = −0.1 between nCount_RNA and percent.mt, and a correlation coefficient of *r* = 0.88 between nCount_RNA and nFeature_RNA (Fig. S1B), indicating good quality of the filtered cell data. Next, we identified highly variable genes in the filtered cells from OS, including a total of 25,841 genes, and performed gene expression variance analysis to identify the top 2,000 variable genes for downstream analysis (Fig. S1C).

The CellCycleScoring function was used to calculate the cell cycle of the samples (Fig. S1D), and the data was normalized. The RunPCA function was employed for PCA on the top 2000 variable genes to reduce the dimensionality of the data. The expression plot of the main correlated genes of the first two principal components is shown selectively (Fig. S1E). An ElbowPlot was generated to rank the principal components by standard deviation, where the “important” components had higher standard deviations (Fig. S1F). Additionally, the JackStrawPlot function was used to visualize the *P* distribution of the top 50 principal components compared to the mean distribution (Fig. S1G). The “important” PCs typically had small *P* (represented by solid lines above the dashed line) and reflected the information contained in the highly variable genes selected earlier. Based on these results, the first 30 principal components adequately represented the information in the selected highly variable genes and were chosen for subsequent UMAP analysis.

Using UMAP clustering analysis, we identified 17 cell clusters, where the cellular composition did not differ significantly between the sample sources but varied in proportions within the cell clusters (Fig. 1B). By searching relevant literature and consulting the CellMarker online database, we annotated the 17 cell clusters into nine cell types (Fig. 1C). These included five immune cell types: B cells (marked by MS4A1 and CD79A), monocytes (marked by LYZ, CD14, and FCGR3A), mast cells (marked by TPSB2 and CPA3), NK/T cells (marked by CD3D, CD3E, CD3G, GNLY, NKG7, KLRD1, and KLRB1), and plasmocytes (marked by IGHG1 and MZB1) (Fig. 1D). Additionally, four non-immune cell types were identified: endothelial cells (marked by PECAM1 and VWF), osteoclasts (marked by ACP5 and CTSK), BMSCs/osteoblastic OS cells (marked by RUNX2, THY1, IBSP, and ALPL), and chondroblastic OS cells (marked by ACAN and SOX9) (Fig. 1D, E).

To accurately identify malignant cells from BMSCs/osteoblastic OS cells and chondroblastic OS cells, we employed the “inferCNV” package to detect widespread CNV, using NK/T cells as a control. The results demonstrated that a majority of BMSCs/osteoblastic OS cells and chondroblastic OS cells exhibited CNV (Fig. 1F). Subsequently, the cells in “Observation” were clustered into six classes, revealing that the fourth class included all normal cells with the lowest CNV score (Fig. 1G). After excluding the fourth class, a total of 6443 malignant OS cells were obtained. Subsequently, we applied a threshold of avg_log2FC > 1 and *P* < 0.05 to select 3805 genes as markers for further analysis.

### Identification of key genes and fusion genes in chemoresistance in OS

**Fig. 2:**
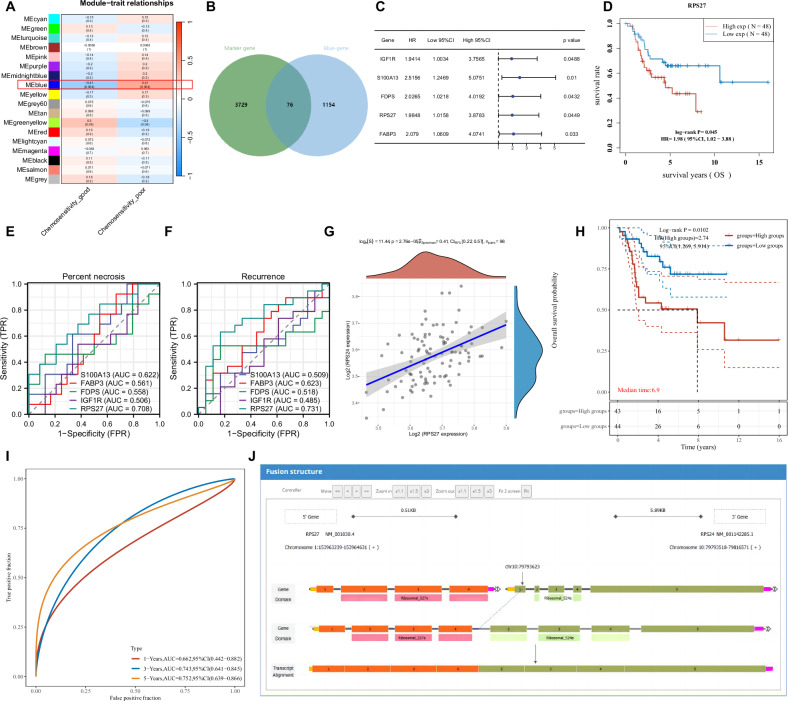
Identification of key genes and fusion genes in chemoresistance in OS using single-cell integrated transcriptomic data. **A** Correlation heatmap between module feature genes and clinical features, each cell contains the corresponding correlation and *p*-value. **B** Venn diagram showing the intersection between blue module genes and malignant OS cell marker genes. **C** Forest plot showing the prediction of overall survival in OS patients using 5 high-risk genes (*N* = 96). **D** Survival curve of patients with high expression of RPS27 (*N* = 48) and low expression of RPS27 (*N* = 48). **E** ROC curve showing the prediction of mortality rate in OS patients using 5 high-risk genes (*N* = 37). **F** ROC curve showing the prediction of tumor recurrence in OS patients using 5 high-risk genes (*N* = 37). **G** Correlation analysis of expression levels between RPS27 and RPS24 (*N* = 96). **H** Survival curve of patients with high expression of RPS24 (*N* = 48) and low expression of RPS24 (*N* = 48). **I** ROC curve showing the prediction of overall survival at 1, 3, and 5 years using RPS24 expression (*N* = 96). **J** Interaction network of the fusion between RPS27 and RPS24 genes.

To identify key genes closely associated with chemoresistance in OS, we obtained RNA-seq data from the GSE154540 dataset in the GEO database and constructed a scale-free co-expression network using the R package WGCNA. Initially, we performed hierarchical clustering on tumor tissue samples from 50 OS patients and removed outliers (Fig. S2A). A soft threshold of *β* = 11 was chosen to establish the scale-free network (Fig. S2B). The parameters minModule size and mergeCutHeight were set to 300 and 0.25, respectively, resulting in the division of the network into 18 modules (Fig. S2C). Furthermore, module-trait correlation analysis revealed that the blue module was significantly associated with chemoresistance in OS (*r* = 0.41, *P* = 0.004) (Figs. 2A and S2D).

Next, we extracted 1230 genes from the blue module and intersected them with the previously identified 3805 malignant OS cell marker genes, resulting in 76 overlapping genes (Fig. 2B). To investigate the association between these intersection genes and the prognosis of OS patients, we downloaded RNA-seq data (TARGET-OS) and corresponding survival information from 96 OS tumor tissue samples in the TARGET database. Univariate survival analysis of the 76 intersection genes revealed that 5 high-risk genes (IGF1R, S100A13, FDPS, RPS27, FABP3) were significantly correlated with overall survival in OS patients (Fig. 2C, D). In addition, using RNA-seq data from 37 OS patients in the GEO dataset GSE39055, along with clinical information on necrosis and tumor recurrence, we performed ROC analysis on the 5 high-risk genes and found that only RPS27 showed good predictive value for necrosis and tumor recurrence in OS patients (Fig. 2E, F). These findings highlight RPS27 as a key gene in chemoresistance in OS identified through single-cell transcriptomic analysis.

RPS27 (Ribosomal Protein S27), also known as Metallopanstimulin-1 or MPS-1, is a ribosomal gene that encodes a component of the 40S subunit. Previous studies have demonstrated the upregulation of RPS27 expression in various human malignancies, such as melanoma [22] and gastric cancer [23]. However, the role and mechanism of RPS27 in OS or chemoresistance have yet to be elucidated, making it a suitable target for our study. Gene fusion events are commonly observed in tumors and have important implications for diagnosis, prognosis, and treatment [24]. Additionally, several studies have reported a close association between fusion genes and tumor chemoresistance [25–28].

To investigate whether there are RPS27-related gene fusions in OS, we first used the FusionGDB2, ChimerDB 3.0, and ChiTaRS 5.0 databases to predict fusion gene pairs involving RPS27. These databases predicted 6, 1, and 3 fusion gene pairs, respectively, and we obtained the intersection of these predictions, resulting in the identification of RPS27-RPS24 as a fusion gene pair. We performed correlation analysis on the expression levels of RPS27 and RPS24 in the TARGET-OS data, which showed a significant positive correlation between them (Fig. 2G). Furthermore, OS patients with high expression of RPS24 exhibited poorer prognosis (Fig. 2H), and RPS24 demonstrated good predictive value for 3-year and 5-year overall survival in OS patients (Fig. 2I). The prediction results from ChimerDB 3.0 also revealed the gene fusion interaction process between RPS27 and RPS24 (Fig. 2J). Therefore, we hypothesize that the RPS27-RPS24 fusion gene may be involved in chemoresistance in OS.

### Overexpression of RPS27-RPS24 confers chemoresistance to OS cells

We selected the most commonly used chemotherapy drug in OS treatment, ADM [29, 30]. U2OS and MG63 cells were cultured in a medium supplemented with ADM at a concentration of 1 µg/ml for 6 months, resulting in the establishment of U2OS/ADMR and MG63/ADMR cells. Subsequently, we assessed the response of these OS cells to ADM and explored the potential correlation between the expression of the fusion gene RPS27-RPS24 and ADM resistance.

**Fig. 3:**
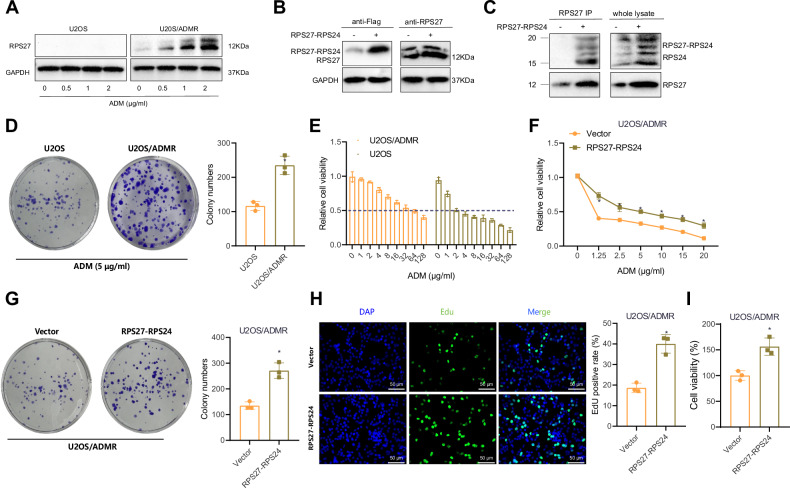
Impact of RPS27-RPS24 on chemoresistance in U2OS cells. **A** Western blot analysis of RPS27 protein expression in U2OS and U2OS/ADMR cells in different groups. **B** Western blot analysis of RPS27 protein expression in U2OS/ADMR cells transfected with either Vector or RPS27-RPS24 overexpression plasmids. **C** IP experiment detecting the expression of RPS27-RPS24 protein in U2OS/ADMR cells transfected with either Vector or RPS27-RPS24 overexpression plasmids. **D** Clonogenic assay measuring cell proliferation of U2OS and U2OS/ADMR cells after treatment with 5 µg/ml ADM, along with the corresponding statistical graph. **E** Sensitivity of U2OS and U2OS/ADMR cells to ADM. **F** MTT assay evaluating cell viability of U2OS/ADMR cells treated with different concentrations of ADM for 48 h. **G** Clonogenic assay measuring cell proliferation of U2OS/ADMR cells treated with 5 µg/ml ADM after transfection with either Vector or RPS27-RPS24 overexpression plasmids, along with the corresponding statistical graph. **H** EdU assay assessing cell proliferation of U2OS/ADMR cells treated with 5 µg/ml ADM after transfection with either Vector or RPS27-RPS24 overexpression plasmids (scale bar: 50 μm), along with the corresponding statistical graph. **I** Treatment of U2OS/ADMR cells with 1 µM Thapsigargin for 24 h, followed by MTT assays to assess cell proliferation after transfection with Vector or RPS27-RPS24 overexpression plasmids. An independent sample *t*-test was used for comparing two groups, while two-way ANOVA was used for comparisons across different time points. * Indicates *P* < 0.05 compared to the U2OS group or Vector group. Cell experiments were performed in triplicates.

The cells were exposed to different concentrations of ADM (0, 0.5, 1, and 2 µg/ml) for 48 h, and the expression of RPS27 was then detected through Western blot analysis. The results showed a significant increase in the levels of RPS27 protein in U2OS/ADMR and MG63/ADMR cells after exposure to different concentrations (1 and 2 µg/ml) of ADM for 48 h, compared to U2OS and MG63 cells treated with different concentrations of ADM. It is worth noting that during the detection of RPS27 protein expression, we observed a distinct protein band above, which exhibited changes parallel to the expression trend of RPS27. This upper band could possibly correspond to RPS27-RPS24 (Figs. 3A and S3A). Next, we constructed an overexpression plasmid for RPS27-RPS24 and transfected it into U2OS/ADMR and MG63/ADMR cells. We observed the detection of a smaller protein band, consistent in size with the protein detected by the anti-Flag antibody and the upper band mentioned earlier (Figs. 3A and S3A). Furthermore, the expression of this protein was significantly increased in the cells overexpressing RPS27-RPS24 (Figs. 3B and S3B). Additionally, IP experiments demonstrated that in cells modified with the fusion gene RPS27-RPS24, a larger protein band was detected by the RPS24 antibody, with a size of ~15–20 kDa larger than the natural RPS27 protein, confirming the expression of RPS27-RPS24 protein (Figs. 3C and S3C). Overall, these results indicate an upregulation of the RPS27-RPS24 fusion gene expression in OS ADM-resistant cells.

Furthermore, as expected, colony formation experiments showed that compared to U2OS and MG63 cells, treatment with ADM (5 µg/ml) did not lead to a decrease in the colony formation ability or a reduction in the size of U2OS/ADMR and MG63/ADMR colonies (Figs. 3D and S3D). Moreover, cell viability assays confirmed that U2OS/ADMR and MG63/ADMR cells were able to tolerate higher concentrations of ADM compared to parental U2OS and MG63 cells. The IC50 of ADM in U2OS and MG63 cells was significantly lower than that in MG63/ADMR and U2OS/ADMR cells, suggesting the potential role of increased RPS27-RPS24 expression in both intrinsic and acquired ADM resistance in OS cells (Figs. 3E and S3E). Thus, it was evident that ADM induced upregulation of the fusion gene RPS27-RPS24 and that increased expression of RPS27-RPS24 may partially contribute to the intrinsic and acquired resistance of OS cells to ADM.

Subsequently, we examined the effect of overexpressing RPS27-RPS24 on the chemoresistance of OS cells using MTT, colony formation, and EdU assays. The results revealed a significant attenuation of the response of U2OS/ADMR and MG63/ADMR cells to ADM after overexpression of RPS27-RPS24, characterized by a weakened inhibitory effect of the drug on cell viability and proliferation (Figs. 3F–H and S3F-H). These findings suggested that the upregulation of RPS27-RPS24 during ADM treatment in OS cells impedes the cytotoxic effect of the drug. We performed MTT assays to assess the ability of RPS27-RPS24 overexpression to resist endoplasmic reticulum (ER) stress in OS cells. The results demonstrated that overexpression of RPS27-RPS24 significantly enhanced the ability of U2OS/ADMR and MG63/ADMR cells to resist the ER stress inducer thapsigargin (Figs. 3I and S3I).

### RPS27-RPS24 regulates a network of DEPs in OS cells

**Fig. 4:**
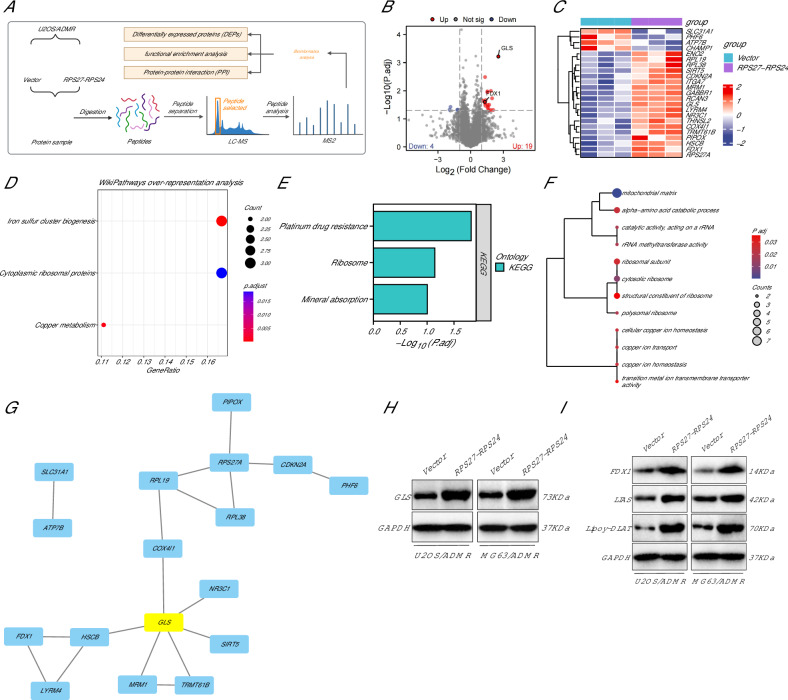
TMT-labeled quantitative proteomic analysis under RPS27-RPS24 intervention. **A** Schematic diagram of the proteomics workflow in this study. **B** Volcano plot analysis of the differences between the Vector group (*N* = 3) and RPS27-RPS24 group (*N* = 3) U2OS/ADMR cell samples, with red representing upregulated proteins, blue representing downregulated proteins, and gray indicating proteins with insignificant differential expression. **C** Expression heatmap of 23 DEPs in the Vector group (*N* = 3) and RPS27-RPS24 group (*N* = 3) U2OS/ADMR cell samples. **D** Bubble plot of the WikiPathways enrichment analysis results for DEPs. **E** Bar plot of the KEGG enrichment analysis results for DEPs. **F** Dendrogram of the GO enrichment analysis results for DEPs. **G** Protein–protein interaction network of DEPs. **H** Western blot analysis of GLS protein expression in cells from each group. **I** Western blot analysis of FDX1, LIAS, and Lipoy-DLAT protein expression in cells from each group, with the cell experiment repeated three times.

We analyzed U2OS/ADMR cell samples with stable transfection of Vector or RPS27-RPS24 using the TMT quantitative proteomics technique. The analysis workflow is shown in Fig. 4A. Through this study, we have revealed that the impact of RPS27-RPS24 on chemoresistance in OS may be related to differential protein expression.

Initially, we identified 7141 protein molecules, applying a threshold of |log2FC| > 1 and *P* < 0.05. We filtered out 23 DEPs, including 19 upregulated proteins (GLS, RPL19, RPS27A, NR3C1, HSCB, RPL38, PIPOX, RCAN3, CDKN2A, MRM1, COX4I1, GABBR1, TRMT61B, ITGA7, LYRM4, THNSL2, ENO2, FDX1, and SIRT5) and 4 downregulated proteins (CHAMP1, PHF6, ATP7B, and SLC31A1) (Fig. 4B, C). Functional enrichment analysis of these 23 DEPs, with a threshold of *P* < 0.05, revealed that they were primarily involved in pathways such as chemoresistance, ribosome, and copper metabolism (Fig. 4D, E). Furthermore, they were enriched in BP, such as copper ion transport, copper ion homeostasis, and alpha-amino acid catabolic processes, CC such as cytoplasmic ribosome, polysome, ribosomal subunit, and cytoplasmic large ribosomal subunit, as well as MF such as rRNA methyltransferase activity, catalytic activity acting on rRNA, and transition metal ion transmembrane transporter activity (Fig. 4F). These findings indicate a close association between RPS27-RPS24 and OS cell copper death.

Subsequently, we constructed a protein–protein interaction (PPI) network for the DEPs and identified the core proteins through topological analysis, with GLS being located at the central position (Fig. 4G). Western blot analysis confirmed a significant upregulation in the expression of GLS in RPS27-RPS24 group OS cells compared to the Vector group (Fig. 4H), further validating the accuracy of the proteomic analysis.

Copper death is characterized by the loss of Fe–S cluster proteins FDX1 and LIAS, as well as reduced protein lipoylation of DLAT. Therefore, we further examined the protein expression of FDX1, LIAS, and Lipoy-DLAT. The results showed a significant upregulation in the expression of FDX1, LIAS, and Lipoy-DLAT in RPS27-RPS24 group OS cells compared to the Vector group (Fig. 4I). Recent evidence suggests that GLS, as a glutaminase, participates in tumor progression by influencing the copper death process [31–33]. This suggests that RPS27-RPS24 may affect OS-resistant cells’ copper death by promoting GLS expression.

In conclusion, we have uncovered the role of a series of DEPs upon overexpression of RPS27-RPS24, with particular emphasis on the central role of GLS and the association of RPS27-RPS24 with copper death. This discovery enhances our understanding of the molecular mechanisms by which RPS27-RPS24 affects chemoresistance in OS, and further investigations will be conducted in follow-up experiments.

### RPS27-RPS24 promotes glutamine metabolism and chemoresistance

**Fig. 5:**
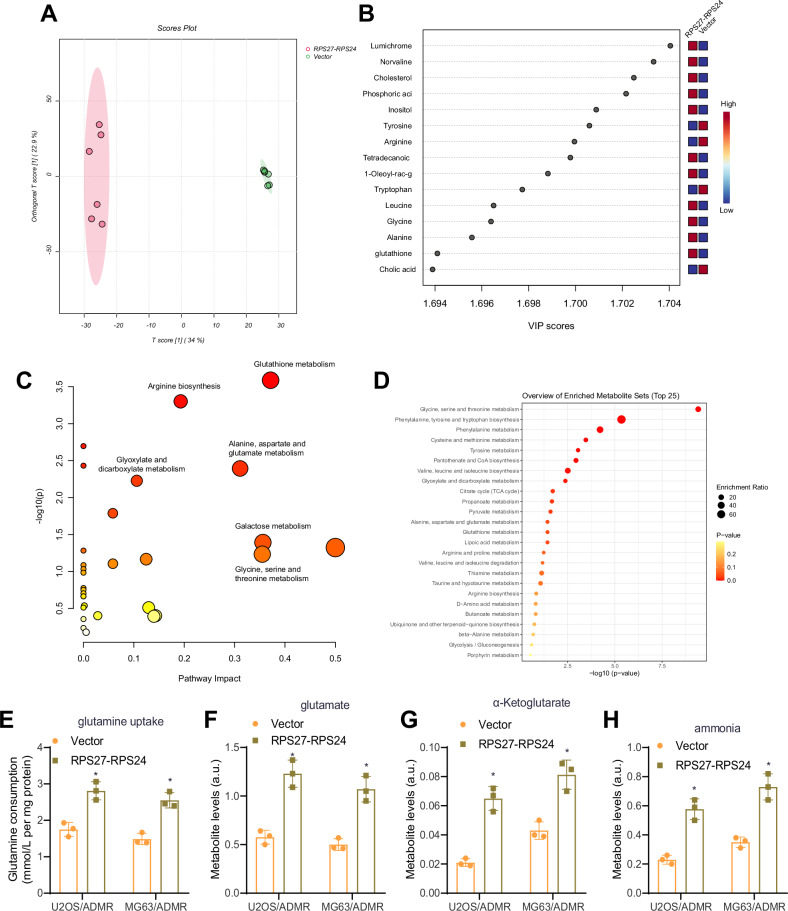
Metabolomic analysis under RPS27-RPS24 intervention. **A** OPLSDA-PCA-2D plot, with a *T* score on the *x*-axis and an Orthogonal *T* score on the *y*-axis. **B** Bubble plot of the top 15 metabolites based on VIP value. **C** Pathway enrichment analysis results of the differential metabolites in the MetaboAnalyst database. **D** Functional enrichment analysis results of the differential metabolites in the MetaboAnalyst database. **E** Glutamine uptake content in cells from each group. **F**–**H** Metabolic levels of glutamate (**F**), α-ketoglutarate (**G**), and ammonia (**H**) in cells after 8 h of glutamine supplementation. The comparison of two sets of data was conducted using an independent samples *t*-test. *Indicates *P* < 0.05 compared to the Vector group, with the cell experiment repeated three times.

To investigate the potential mechanism by which RPS27-RPS24 functions further, we collected culture supernatant samples from U2OS/ADMR cells transfected with a stable vector or RPS27-RPS24 for untargeted metabolomics analysis. The analysis process is outlined in Fig. S4A. Firstly, we conducted PCA using EZinfo software to analyze the acquired mass spectrometry data, as shown in Fig. S4B, C. Differential metabolites (highlighted in red boxes) were selected based on a VIP > 1 cutoff (Fig. S4D). Subsequently, data were further corrected using the MetaboAnalyst website (Fig. S4E), fold change and *P* were calculated, and differentially expressed metabolites were identified using a threshold of |log2FC| > 2 and *P* < 0.05, resulting in 32 differential metabolites (Fig. S4F, G). The VIP values of each metabolite were calculated using the OPLSDA algorithm, and the top 15 metabolites with the highest VIP values were presented (Fig. 5A, B). Pathway and functional enrichment analysis revealed that the glutamine metabolism pathway was prominently enriched (Fig. 5C, D). These results suggest that RPS27-RPS24 may be involved in OSchemoresistance by regulating glutamine metabolism.

Glutamine is a non-essential amino acid that plays a crucial role in cellular metabolism. The metabolic products of glutamine include: (1) glutamate, which is converted from glutamine through the action of GLS; (2) α-ketoglutarate, a key intermediate of the tricarboxylic acid (TCA) cycle that is generated from glutamine by further deamination; (3) ammonia, which is released during glutamine conversion to α-ketoglutarate [34]. To assess the level of glutamine intake in the culture medium, we used a YSI 7100 multi-channel biochemical analyzer. Additionally, the concentrations of metabolites (glutamate, α-ketoglutarate, and ammonia) were measured using high-performance liquid chromatography (HPLC), and the changes in metabolite concentrations relative to fresh growth medium were normalized to protein content per well. We found that compared to the Vector Group, the RPS27-RPS24 group had higher levels of glutamine intake and significantly increased concentrations of glutamate, α-ketoglutarate, and ammonia (Fig. 5E–H), consistent with the results of the metabolomics analysis. These findings suggest that overexpression of RPS27-RPS24 promotes glutamine metabolism.

In line with previous studies linking GLS-driven glutamine catabolism to the occurrence and progression of prostate cancer and pancreatic cancer [35, 36], as well as enhanced glutamine hydrolysis driving hypoxia-induced chemoresistance in pancreatic cancer [37], we hypothesize that RPS27-RPS24 may promote glutamine metabolism by upregulating GLS expression, which may be closely associated with OSchemoresistance and copper death.

### Reversal of chemoresistance by GLS knockout and inhibition of copper death in OS cells

**Fig. 6:**
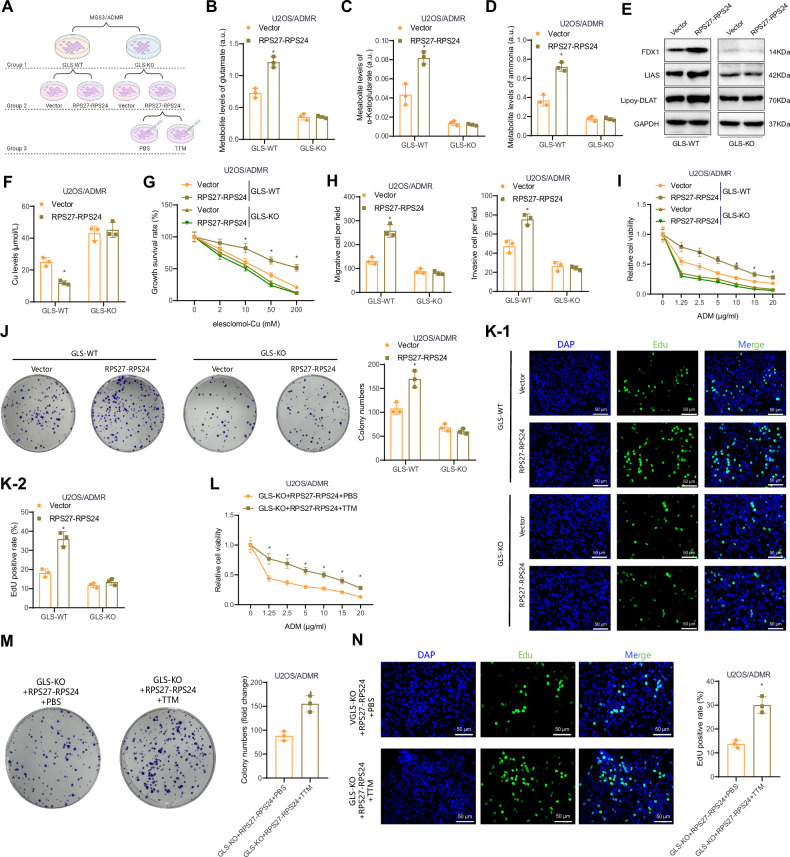
The impact of RPS27-RPS24 on GLS-mediated glutamine metabolism on U2OS/ADMR cells in copper death and chemoresistance. **A** Schematic representation of the cell grouping process, using U2OS/ADMR as an example. **B**–**D** Represents the metabolic levels of glutamate (**B**), α-ketoglutarate (**C**), and ammonia (**D**) in the cells after 8 h of culture in the presence of glutamine medium. **E** Western blot analysis of FDX1, LIAS, and Lipoy-DLAT protein expression in the cells of each group. **F** Copper ion content in the cells of each group. **G** Cell viability was determined by CCK-8 assay after 72 h of treatment with different concentrations of elesclomol–Cu (ratio = 1:1). **H** Cell migration capability assessed by scratch experiment (upper image) (scale bar: 200 μm) and cell invasion ability assessed by Transwell assay (lower image) (scale bar: 100 μm) after 72 h of treatment with 50 mM elesclomol–Cu (ratio = 1:1). **I** Cell viability of GLS-WT and GLS-KO U2OS/ADMR cells determined by MTT assay. **J** Cell proliferation of GLS-WT and GLS-KO groups assessed by clone formation experiment and statistical analysis after treatment with 5 µg/ml ADM (scale bar: 50 μm). **K** Cell proliferation of GLS-WT and GLS-KO groups assessed by EdU assay and statistical analysis after treatment with 5 µg/ml ADM (scale bar: 50 μm). **L** Cell viability of GLS-KO + RPS27-RPS24 + PBS and GLS-KO + RPS27-RPS24 + TTM groups determined by MTT assay. **M** Cell proliferation of GLS-KO + RPS27-RPS24 + PBS and GLS-KO + RPS27-RPS24 + TTM groups assessed by clone formation experiment and statistical analysis after treatment with 5 µg/ml ADM. **N** Cell proliferation of GLS-KO + RPS27-RPS24 + PBS and GLS-KO + RPS27-RPS24 + TTM groups assessed by EdU assay and statistical analysis after treatment with 5 µg/ml ADM. For the comparison of data across different time periods or factors, a two-way ANOVA was conducted. *Indicates *P* < 0.05 compared to the Vector group or the GLS-KO + RPS27-RPS24 + PBS group, and all cell experiments were repeated three times.

Cell grouping was performed as shown in Fig. 6A (using U2OS/ADMR as an example). Firstly, U2OS/ADMR and MG63/ADMR cell lines with GLS knockout were constructed using CRISPR/Cas9 gene editing technology (Fig. S5A). The efficiency of gene knockout was validated through RT-qPCR and Western blot analysis, which revealed a significant reduction in GLS expression levels in GLS-KO U2OS/ADMR and MG63/ADMR cells compared to GLS-WT cells (Fig. S5B, C). This demonstrates the successful knockout of GLS. The impact of GLS knockout on chemoresistance in OS cells was assessed through MTT, colony formation, and EdU assays. The results revealed a significant enhancement in the response of GLS-KO cells to ADM compared to GLS-WT cells, evidenced by the increased inhibitory effect of the drug on cell viability and proliferation (Fig. S6A–C). This indicates that the knockout of GLS considerably weakens chemoresistance in OS cells.

We also measured the concentrations of glutamine metabolism products (glutamate, α-ketoglutarate, and ammonia) in the different groups of cells. The results showed a significant increase in the concentrations of these metabolites in the RPS27-RPS24 group of GLS-WT cells compared to the Vector group. However, there were no significant differences in the concentrations of these metabolites between the Vector group and the RPS27-RPS24 group in GLS-KO cells (Figs. 6B–D and S7A–C). These findings demonstrate that the knockout of GLS effectively reverses the promoting effect of RPS27-RPS24 overexpression on glutamine metabolism.

Next, we evaluated the protein expression levels of copper death-related factors and the copper ion content in the different groups of cells. The results showed a significant upregulation of FDX1, LIAS, and Lipoy-DLAT protein expression and a significant reduction in copper ion levels in the RPS27-RPS24 group of GLS-WT cells compared to the Vector group. However, no significant differences were observed in protein expression levels between the Vector group and the RPS27-RPS24 group in GLS-KO cells (Figs. 6E, F and S7D, E). Additionally, we treated the cells with different concentrations of elesclomol–Cu (ratio = 1:1) for 72 h and assessed cell viability through CCK-8 assays, cell migration through scratch assays, and cell invasion using Transwell assays. The results showed that the RPS27-RPS24 group of GLS-WT cells exhibited higher cell viability, enhanced migration, and invasion capabilities compared to the Vector group. However, there were no significant differences in cell viability, migration, and invasion capabilities between the Vector group and the RPS27-RPS24 group in GLS-KO cells (Figs. 6G, H and S7F, G). This indicates that the knockout of GLS significantly reverses the inhibitory effect of RPS27-RPS24 overexpression on copper death in drug-resistant OS cells.

To further confirm whether RPS27-RPS24 affects copper death through GLS-mediated glutamine metabolism and ultimately promotes chemoresistance, we treated cells overexpressing RPS27-RPS24 with the glutamine metabolism inhibitor CB-839 [38]. The cells were divided into the RPS27-RPS24 + PBS group and the RPS27-RPS24 + CB-839 group, and changes in copper death-related indicators were assessed. It was found that compared to the RPS27-RPS24 + PBS group, the RPS27-RPS24 + CB-839 group exhibited significant downregulation of FDX1, LIAS, and Lipoy-DLAT protein expression, a significant increase in copper ion levels, lower cell viability after elesclomol-Cu treatment, and reduced migration and invasion capabilities (Fig. S8A–D). This provides further evidence that RPS27-RPS24 promotes the inhibition of copper death through GLS-mediated glutamine metabolism.

The results of chemoresistance-related assays showed that the RPS27-RPS24 group of GLS-WT cells exhibited significantly reduced response to ADM compared to the Vector group, resulting in a weaker inhibitory effect of the drug on cell viability and proliferation. However, there were no significant differences in response to ADM between the Vector group and the RPS27-RPS24 group in GLS-KO cells (Figs. 6I–K, and S7H–J). These results indicate that the knockout of GLS significantly reverses the promoting effect of RPS27-RPS24 overexpression on chemoresistance in OS cells.

Finally, to clarify the impact of copper death on chemoresistance and whether RPS27-RPS24 promotes chemoresistance through regulating GLS expression to inhibit copper death, GLS-KO cells transfected with RPS27-RPS24 plasmids were treated with PBS or the copper death inhibitor TTM. The results showed a significantly reduced response to ADM in the GLS-KO + RPS27-RPS24 + TTM group compared to the GLS-KO + RPS27-RPS24 + PBS group (Figs. 6L–N and S7K–M). This indicates that the inhibition of copper death promotes chemoresistance in OS cells, and the specific mechanism involves RPS27-RPS24 promoting GLS-mediated glutamine metabolism to inhibit copper death and ultimately promote chemoresistance.

### RPS27-RPS24 promotes in vivo tumor growth and chemoresistance via enhanced glutamine metabolism

**Fig. 7:**
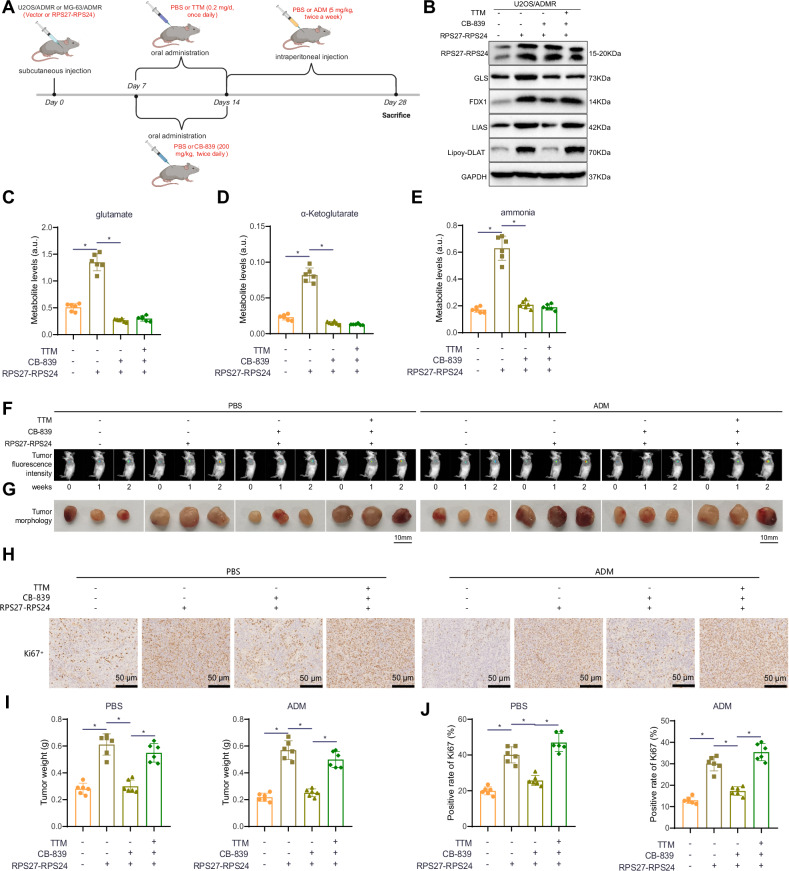
Impact of RPS27-RPS24 on tumorigenesis and chemoresistance in U2OS cells through regulation of glutamine metabolism and copper death. **A** Schematic representation of the animal grouping process. **B** Western blot analysis of RPS27-RPS24, GLS, FDX1, LIAS, and Lipoy-DLAT protein expression levels in the tumor tissues of each group of mice. **C**–**E** Metabolic levels of glutamate (C), α-ketoglutarate (**D**), and ammonia (**E**) in the tumor tissues of each group of mice. **F** Measurement of tumor growth at different time points using bioluminescence intensity. **G** Morphology of tumor tissues from each group, with three representative examples shown for each group. **H** Immunohistochemical staining to determine protein expression levels of Ki67 in the tumor tissues of each group (scale bar = 50 μm). **I** Tumor tissue weights of each group. **J** Statistical analysis of Ki67-positive expression. For comparing data between different groups, one-way ANOVA was utilized. *Indicates *P* < 0.05 compared to the Vector + PBS + PBS group or the RPS27-RPS24 + PBS + PBS group or the RPS27-RPS24 + CB-839 + PBS group, with six mice per group.

The above paragraph describes the results of an in vitro cell experiment investigating the role of RPS27-RPS24 in promoting GLS-mediated glutamine metabolism inhibition and chemoresistance in OS cells. To validate whether this mechanism affects the tumorigenic capability of OS cells in vivo, we injected OS cells overexpressing RPS27-RPS24 into the axilla of mice to establish subcutaneous xenograft tumor models. The mice were treated with either the glutamine metabolism inhibitor CB-839 or the copper death inhibitor TTM, starting from day 7, via intraperitoneal injection of a PBS solution or 5 mg/kg ADM treatment (Fig. 7A).

Firstly, Western blot analysis revealed that compared to the Vector+PBS + PBS group, the RPS27-RPS24 + PBS + PBS group of mice showed a significant increase in the expression levels of RPS27-RPS24, GLS, FDX1, LIAS, and Lipoy-DLAT proteins in tumor tissues. In comparison to the RPS27-RPS24 + PBS + PBS group, the RPS27-RPS24 + CB-839 + PBS group exhibited no significant change in RPS27-RPS24 expression and a significant decrease in the expression of GLS, FDX1, LIAS, and Lipoy-DLAT proteins. Additionally, compared to the RPS27-RPS24 + CB-839 + PBS group, the RPS27-RPS24 + CB-839 + TTM group showed no significant changes in RPS27-RPS24 and GLS expression but had a significant increase in the expression of FDX1, LIAS, and Lipoy-DLAT proteins (Figs. 7B and S9A). These results suggest that RPS27-RPS24 overexpression can promote GLS expression and inhibit copper death, while the glutamine metabolism inhibitor can reverse the inhibitory effect of RPS27-RPS24 overexpression on copper death. Furthermore, the addition of the copper death inhibitor can further reverse the effect of the glutamine metabolism inhibitor.

We also measured the concentrations of glutamine metabolism products in the tumor tissues of each group of mice. The results showed that compared to the Vector+PBS + PBS group, the RPS27-RPS24 + PBS + PBS group of mice had significantly increased levels of glutamate, α-ketoglutarate, and ammonia in tumor tissues. In comparison to the RPS27-RPS24 + PBS + PBS group, the RPS27-RPS24 + CB-839 + PBS group exhibited significant decreases in the concentrations of all metabolites. There were no significant changes in the concentrations of these metabolites between the RPS27-RPS24 + CB-839 + PBS group and the RPS27-RPS24 + CB-839 + TTM group (Figs. 7C–E and S9B–D). These findings indicate that RPS27-RPS24 overexpression can promote glutamine metabolism and increase the production of glutamate, α-ketoglutarate, and ammonia. The glutamine metabolism inhibitor can reverse the effect of RPS27-RPS24 overexpression, while the addition of the copper death inhibitor has no effect on glutamine metabolism. This suggests that RPS27-RPS24 inhibits copper death by promoting glutamine metabolism.

After one and two weeks, tumor growth in mice was assessed using bioluminescent imaging. At week 4, tumor tissue was excised to observe tumor morphology, measure tumor weight, and perform Ki67 immunohistochemical staining. In the PBS treatment group, compared to the Vector+PBS + PBS group, the RPS27-RPS24 + PBS + PBS group of mice exhibited faster tumor growth, a significant increase in tumor weight, and a significant increase in Ki67 protein positivity. In comparison to the RPS27-RPS24 + PBS + PBS group, the tumor growth rate, tumor weight, and Ki67 protein positivity were significantly reduced in the RPS27-RPS24 + CB-839 + PBS group. Moreover, compared to the RPS27-RPS24 + CB-839 + PBS group, the RPS27-RPS24 + CB-839 + TTM group showed faster tumor growth, increased tumor weight, and a significant increase in Ki67 protein positivity (Figs. 7F, J and S9E–I). When compared to the PBS treatment group, all the groups treated with ADM showed slower tumor growth, lighter tumor weight, and lower Ki67 protein positivity, with similar changes as observed in the PBS group (Figs. 7F, J and S9E–I). These results demonstrate that RPS27-RPS24 overexpression can promote tumor growth and chemoresistance in OS cells. The glutamine metabolism inhibitor can reverse the promoting effect of RPS27-RPS24 overexpression on tumor growth and chemoresistance, while the addition of the copper death inhibitor can further reverse the effect of the glutamine metabolism inhibitor.

In summary, RPS27-RPS24 overexpression can promote glutamine metabolism, inhibit copper death, and thereby promote tumor growth and chemoresistance in OS cells.

## Discussion

One of the significant challenges in OS is its resistance to chemotherapy, which has been widely documented [39, 40]. The development of chemoresistance is a major cause of treatment failure and recurrence in tumors [41, 42]. Therefore, investigating the molecular mechanisms underlying chemoresistance is of great significance in enhancing OS treatment efficacy [43, 44]. The aim of this study is to explore the molecular mechanisms of a novel fusion gene, RPS27-RPS24, in chemoresistance, providing new insights and approaches for the prevention and treatment of chemoresistance in OS.

To date, several key genes related to chemoresistance have been identified in previous studies [45–47]. However, in comparison to previous research, our study discovered a new fusion gene, RPS27-RPS24. Comparative analysis revealed differences in the mechanism of action of RPS27-RPS24 in chemoresistance in OS when compared to previously discovered key genes. Furthermore, we have utilized data from previous studies to support and compare our study findings.

In previous research, various metabolic regulation mechanisms and signaling pathways related to chemoresistance in OS have been described [48, 49]. However, in this study, we unveil new findings regarding the role of RPS27-RPS24 in GLS-mediated glutamine metabolism regulation and copper death. These novel discoveries provide important clues and new research directions for further understanding the mechanisms of chemoresistance in OS.

There are differences in experimental methods and techniques between previous studies and this study. Compared to previous research, this study utilizes more advanced and precise experimental methods to comprehensively and accurately analyze the regulatory role of RPS27-RPS24 in chemoresistance in OS. When analyzing the consistency and differences in research results, we found that the novelty of this study lies in uncovering a novel regulatory mechanism of RPS27-RPS24 in chemoresistance.

In this study, fusion genes related to OS prognosis and chemoresistance were screened using single-cell transcriptome data. Downstream key proteins and metabolic pathways were identified through proteomics and metabolomics techniques. By integrating in vivo and in vitro experimental results, we have come to the following conclusions: the novel fusion gene RPS27-RPS24 promotes GLS-mediated glutamine metabolism, inhibits copper death, and ultimately enhances OS cell chemoresistance, thereby promoting the progression of OS. Comparing and evaluating the results with previous studies, we found some similarities with previous research and also revealed some inconsistencies. These findings are important not only for the understanding of OS development and treatment but also for providing new ideas and directions for the development of new therapeutic strategies and drug targets.

Our study demonstrates that RPS27-RPS24 influences copper-induced cell death mechanisms by promoting GLS-mediated glutamine metabolism. Copper concentration within the cell is closely associated with mitochondrial function. Elevated copper ion levels may alter mitochondrial membrane potential, which is often linked to cell death processes [50]. Specifically, copper-induced cell death may lead to oxidative stress and mitochondrial damage, causing a loss of mitochondrial membrane potential [51]. Upregulation of GLS could further affect mitochondrial health by modulating energy metabolism, influencing cell survival and death pathways. Based on our findings, RPS27-RPS24 may play a crucial role in copper-induced cell death through glutamine metabolism regulation, linking this process to the maintenance of mitochondrial membrane potential. This discovery suggests that exploring the relationship between copper ions and cell metabolism could offer new approaches to overcoming OS chemoresistance.

In this study, our focus was on the role of RPS27 in cell metabolism, not directly on its relationship with autophagy or mitophagy. However, existing research highlights that autophagy is vital for cellular homeostasis and metabolic regulation, with RPS27 potentially playing a significant role in this process [52]. For instance, RPS27 upregulation may influence autophagy through the mTOR pathway, promoting cell survival under stress [53]. Mitophagy, a specific form of autophagy, is essential for removing damaged mitochondria to maintain cellular energy balance and function. Studies suggest that RPS27 might indirectly affect mitophagy by regulating the cellular response to oxidative stress [54]. Future research should explore the connections between RPS27, autophagy, and mitophagy to comprehensively understand its biological functions.

We also investigated the combined effects of ADM and other treatments on OS cell lines U2OS and MG-63, focusing on copper-induced cell death and ER stress. Our experiments reveal that the RPS27-RPS24 fusion gene plays a critical role in regulating glutamine metabolism and copper-induced cell death. Specifically, ADM treatment followed by GLS knockdown and RPS27-RPS24 overexpression experiments demonstrated that the copper death inhibitor TTM significantly weakened the cells’ response to ADM. This indicates that inhibiting copper-induced cell death may be a key mechanism of chemoresistance in OS cells. The RPS27-RPS24 fusion gene enhances resistance to copper-induced cell death through GLS-mediated glutamine metabolism, affecting the efficacy of chemotherapy. These findings align with current literature showing that glutamine metabolism is crucial for tumor cell survival and resistance [55]. Additionally, our results suggest that the RPS27-RPS24 fusion gene enhances resistance to ER stress, indicating that it may regulate ER stress responses and influence chemotherapy sensitivity. ER stress has been shown to play a vital role in tumor cell survival and resistance [56]. Thus, the role of RPS27-RPS24 in OS chemoresistance involves complex interactions between copper-induced cell death and ER stress regulation. We plan to further investigate these mechanisms in future studies to reveal potential therapeutic applications for RPS27-RPS24 in OS treatment.

Although we have not specifically identified the post-translational modifications (PTMs) of the RPS27-RPS24 fusion protein in this study, the literature indicates that PTMs are crucial in regulating protein function, stability, and interactions [57]. Known PTMs include phosphorylation, ubiquitination, acetylation, and methylation, which may influence the biological function of RPS27-RPS24 [58]. Phosphorylation, for example, plays a key role in cell signaling and protein activity regulation [59], while ubiquitination is closely related to protein degradation and cell cycle control [60]. Understanding how these PTMs affect the fusion protein’s regulation of GLS-mediated glutamine metabolism and copper-induced cell death will be essential for elucidating its role in chemoresistance. Future studies should focus on identifying these PTMs and investigating their effects on the fusion protein’s functions and its role in cancer cell survival and stress responses. Moreover, the RPS27-RPS24 fusion protein may participate in forming multiprotein complexes, interacting with other ribosomal and signaling proteins to regulate metabolic pathways and chemoresistance. Exploring these interactions will further our understanding of RPS27-RPS24’s role in tumor biology.

While important discoveries have been made in this study, there are limitations in the methods and data. Due to the limited sample size, our research results may have certain biases. Additionally, we face technical and time limitations. To further validate and strengthen the results of this study, we will focus on expanding the sample size, adopting more advanced experimental techniques, and improving the accuracy of data analysis.

In summary, this study has discovered the regulatory role of the novel fusion gene RPS27-RPS24 in chemoresistance in OS and revealed its specific molecular mechanism. This discovery deepens our understanding of the mechanisms underlying chemoresistance in OS and has significant scientific value. These results provide new targets and strategies for OS treatment and offer new directions and potential for precision medicine development.

The results of this study have important clinical applications, especially in chemoresistance reversal and precision treatment. Based on a deep understanding of RPS27-RPS24 regulatory mechanisms, we can develop new treatment strategies and drug designs targeting this fusion gene. However, further research and challenges are still needed in the future to overcome the limitations of this study and to enhance improvements and developments in OS treatment. We will commit to conducting more clinical trials and in-depth research to fully understand the clinical potential of RPS27-RPS24 in OS and to explore more targeted treatment methods and strategies to provide patients with more effective and personalized treatment options.

## Materials and methods

### Ethical statement

All experiments involving mice were approved by Jiangxi Cancer Hospital (2024ky058).

### Public data download

OS-related scRNA-seq data GSE162454 and transcriptome RNA sequencing (RNA-seq) data GSE154540, GSE39055 were obtained from the Gene Expression Omnibus (GEO, https://www.ncbi.nlm.nih.gov/geo/). The GSE162454 dataset includes tumor tissue samples from 6 OS patients. The GSE154540 dataset contains tumor tissue samples from 29 chemotherapy-sensitive OS patients and 21 chemoresistance OS patients. The GSE39055 dataset consists of tumor tissue samples from 37 OS patients. In addition, RNA-seq data (TARGET-OS) of tumor tissue from 96 OS patients, along with clinical information, were downloaded from the TARGET database (https://ocg.cancer.gov/programs/target) (Table S1) [61]. Since these data are sourced from public databases, ethical committee approval is not required.

### scRNA-seq data analysis

The scRNA-seq data (GSE162454) was analyzed using the R software package Seurat [62]. First, the data was subjected to quality control based on the following criteria: 200 < nFeature_RNA < 5000, 200 < nCount_RNA < 20,000, and percent.mt < 25. Next, batch effects were eliminated using the CCA method. The data, which was normalized using the LogNormalize function, underwent principal component analysis (PCA) using the RunPCA function. The most significant principal components were selected for UMAP clustering analysis through the use of the JackStrawPlot and ElbowPlot functions. CellMarker database (http://bio-bigdata.hrbmu.edu.cn/CellMarker/) was used for cell annotation [63] by obtaining marker genes for each cell cluster with the FindAllMarkers function. DotPlot and VlnPlot functions were employed to visualize the expression of marker genes in different cell clusters. Additionally, putative malignant cells were identified using the subset function. These cells underwent copy number variation (CNV) analysis using the inferCNV package in R. *K*-means algorithm was utilized to exclude non-malignant cells, resulting in the identification of marker genes specific to malignant cells.

### Bioinformatics analysis

The gene expression clustering analysis and phenotype association analysis of genes in the GSE154540 dataset were performed using the R software package WGCNA, known as weighted gene co-expression network analysis. This analysis aimed to identify important gene modules related to chemoresistance in OS and extract the genes within those modules [64].

The intersection of the module genes and malignant OS cell marker genes was obtained through the jvenn website. Subsequently, single-gene survival analysis was conducted on the intersection genes based on the TARGET-OS data, with overall survival as the predicted parameter. The survival package in R was used to perform the proportional hazards assumption test and to establish a fitted survival regression model. The results were visualized using the survminer and ggplot2 packages in R. Log-rank tests were employed to display survival curves and compare the overall survival differences between the high-expression group and the low-expression group. A significance level of *P* < 0.05 was considered [65]. Additionally, the candidate genes were subjected to receiver operating characteristic (ROC) analysis using the R package pROC, based on the GEO dataset GSE39055, to evaluate their predictive value in OS patient mortality and tumor recurrence.

FusionGDB2 (https://compbio.uth.edu/FusionGDB2/index.html), ChimerDB 3.0 (https://www.kobic.re.kr/chimerdbv3/mindex.cdb), and ChiTaRS 5.0 (http://biosrv.org/chmb/search) databases were utilized to retrieve fusion gene pairs related to RPS27 [66–68]. Moreover, the ChimerDB 3.0 database was used to obtain the gene fusion interaction network involving RPS27 and RPS24. Subsequently, based on the TARGET-OS data, R software package corplot was employed to analyze the correlation between the expression levels of RPS27 and RPS24.

### Cell culture

The human OS cell lines U2OS (HTB-96) and MG-63 (CRL-1427) were obtained from ATCC (USA). U2OS and MG-63 cells were cultured in McCoy’s 5A medium (16600082, Gibco, USA) supplemented with 10% FBS (10099141, Gibco, USA), 10 μg/ml streptomycin, and 100 U/ml penicillin (15140148, Gibco, USA).

The 293 T cell line was purchased from ATCC (CRL-3216) and cultured in DMEM medium (11965092, Gibco, USA) supplemented with 10% FBS, 10 μg/ml streptomycin, and 100 U/ml penicillin. All cells were cultured in a humidified incubator at 37 °C with 5% CO_2_ (Heracell™ Vios 160i CR CO_2_ incubator, 51033770, Thermo Scientific™, Germany). When the cells reached 80–90% confluency, subculturing was performed [69].

To establish U2OS/adriamycin-resistant (ADMR) and MG63/ADMR cells, U2OS and MG63 cells were cultured in a medium supplemented with ADM (D5220, Sigma-Aldrich, USA) at a concentration of 1 µg/ml for 6 months [70].

### Construction and transfection of fusion genes

Specific primers were designed to amplify the corresponding regions of RPS27 and RPS24 genes. The PCR products were purified, and the digested fragments of RPS27 and RPS24 were obtained using the respective restriction enzymes. The digested fragments of RPS27 and RPS24 were ligated together to form the fusion gene RPS27-RPS24. The fusion gene was then inserted into the mammalian expression vector pCDNA3.1 to generate the recombinant expression vector. Finally, the bacterial transformation and selection of resistant clones were conducted, followed by sequencing validation to confirm the correct expression vector of the RPS27-RPS24 fusion gene [13].

The RPS27-RPS24 coding sequences were cloned into the pAd-DsRed-IRES-EGFP adenovirus reporter vector. 293 T cells were cultured in a 6-well plate for 2–3 weeks and infected with the amplified adenovirus. After the production of adenovirus, the lysates were harvested. The constructed RPS27-RPS24 fusion gene adenovirus (MOI = 10, virus titer 1 × 10^8^ TU/mL) was then transfected into appropriate U2OS/ADMR and MG63/ADMR cells. The infected cells were screened with puromycin (2 μg/ml, HY-K1057, Med Chem Express, USA) for 7 days and validated through protein-level experiments [12].

### Validation of the Binding of RPS27 and RPS27-RPS24 through Co-immunoprecipitation (Co-IP)

U2OS/ADMR or MG-63/ADMR cells were seeded in 6 cm culture dishes and treated with 10 μM proteasome inhibitor MG132 (HY-13259, Med Chem Express, USA) for 6 h. The cells were then collected and lysed using NP-40 lysis buffer (P0013F, Bioteke Corporation, Shanghai, China). Forty micrograms of total protein were prepared as the input group, and the remaining protein was adjusted to a concentration of 1 mg/mL in 1 mL and divided into three tubes. Anti-RPS27 antibody (10 μg, 114838, Beijing NuoPu Bio-Technology Co., Ltd., China) or anti-Flag antibody (ab205606, Abcam, UK) or IgG (ab6715, 1:1000, Abcam, UK) antibody (10 μg) was added to the total protein and incubated with slow agitation overnight at 4 °C. Protein A/G agarose (sc-2003, Santa Cruz Biotechnology, USA) was added and incubated for 4 hours at 4 °C, followed by three washes with pre-chilled TBS solution. Immunoprecipitated proteins were detected by protein immunoblot analysis [71, 72].

### Protein sample preparation and assessment

U2OS/ADMR cells overexpressing Stable Trans Vector (*N* = 3) or RPS27-RPS24 (*N* = 3) were transferred to 5 cm^3^ centrifuge tubes. Subsequently, ultrasonication under ice bath conditions was performed using a SCIENTZ-IID ultrasonic cell disruptor (Scientz, Ningbo, China) with a phenol extraction buffer containing 10 mM DTT (R0861, Solarbio, Beijing, China), 1% protease inhibitor cocktail (P6731, Solarbio, Beijing, China) and 2 mM EDTA (E1170, Solarbio, Beijing, China). This step was repeated 8 times. Next, an equal volume of Tris-saturated phenol (pH 8.0) (HC1380, Vander Biological, Beijing, China) was added, and the mixture was vortexed for 4 min. Subsequently, centrifugation at 5000×*g* for 10 minutes at 4 °C was performed to transfer the upper phase of phenol to a new centrifuge tube. The phenol solution was supplemented with 0.1 M ammonium sulfate (101217, Merck, USA) and saturated methanol (106035, Merck, USA) in a 1:5 volume ratio and left overnight to precipitate the proteins. Afterward, centrifugation at 4 °C for 10 min was carried out to remove the supernatant. Finally, the remaining precipitate was washed once with cold methanol and three times with cold acetone. The washed protein was then dissolved in 8 M urea (U8020, Solarbio, Beijing, China), and its concentration was determined using the BCA assay kit (P0012, BiyunTian, Shanghai, China) according to the manufacturer’s instructions [73].

### Protease digestion, peptide labeling, and nano-LC–MS/MS analysis

For each sample, 50 µg of protein was subjected to enzymatic digestion. The protein solution was mixed with DTT to a final concentration of 5 mM and incubated at 56 °C for 30 min. Then, acetonitrile was added to achieve a concentration of 11 mM, and the mixture was incubated at room temperature for 15 minutes. Finally, the urea concentration of the sample was diluted to less than 2 M, and trypsin (25200056, Thermo Fisher Scientific, USA) was added at a 1:50 (w/w) ratio to the protein and incubated overnight at 37 °C. Subsequently, trypsin digestion continued for an additional 4 h at a 1:100 (trypsin:protein) ratio.

Following trypsin digestion, peptide desalting was performed using a HyperSep™ C18 purification column (60108-302, Thermo Fisher Scientific, USA), followed by vacuum drying. The peptides were then reconstituted in 0.5 M TEAB (90114, Thermo Fisher Scientific, USA) and processed according to the manufacturer’s instructions for the TMT reagent kit (90064CH, Thermo Fisher Scientific, USA). In brief, one unit of the TMT reagent was thawed and reconstituted in acetonitrile (113212, Merck, USA). The peptide mixture was incubated at room temperature for 2 h, followed by desalting and drying using a vacuum centrifugal concentrator. Equal amounts of each labeled peptide fraction from different groups were combined, and the dried peptides were resuspended in 0.1% formic acid (159002, Merck, USA).

For nano-LC separation, 2 µg of peptides from each sample were loaded onto an Easy nLC 1200 nano-UPLC system (Thermo Fisher Scientific, USA). The sample was first trapped onto a Trap C18 column (100 µm × 20 mm, 5 µm) and then separated on a C18 analytical column (75 µm × 150 mm, 3 µm) using a gradient elution at a flow rate of 300 nL/min. The mobile phase included 0.1% formic acid in water as solvent A and 0.1% formic acid in water-acetonitrile (containing 95% acetonitrile) as solvent B. The gradient elution program was as follows: 0 → 2 min, 2% → 8% B; 2 → 71 min, 8% → 28% B; 71 → 79 min, 28% → 40% B; 79 → 81 min, 40% → 100% B; 81 → 90 min, 100% B. The separated peptides were then subjected to mass spectrometric analysis on a Q-Exactive HFX mass spectrometer (Thermo Fisher Scientific, USA). The analysis lasted for 60 min with an electrospray voltage of 2.1 kV. The detection mode was positive ion mode with a survey scan range of 350–1200*m*/*z*. The resolution of the primary mass spectrometry was set at 60,000 (FWHM at *m*/*z* 200) with an AGC target of 3e6 and a maximum injection time of 30 ms. The resolution of the secondary mass spectrometry was set at 15,000 (FWHM at *m*/*z* 200) with an AGC target of 1e6 and a maximum injection time of 25 ms. HCD was used as the activation type for MS2, with an isolation window of 20 Th and a normalized collision energy of 32 [73, 74].

### Proteomic data analysis

The LC–MS/MS data obtained were processed using MaxQuant software (v.1.5.2.8), which included peptide identification and protein quantification. For tandem mass spectrometry searching, UniProt 14.1 (2009)—Gossypium hirsutum database and the reversed decoy database were used. Trypsin was selected as the cleavage enzyme, with up to 2 missed cleavages allowed. The initial search used a mass tolerance of 20 ppm, followed by a main search using 5 ppm and a fragment ion mass tolerance of 0.02 Da. Peptide false discovery rate (FDR) ≤ 0.01, protein FDR ≤ 0.01, and peptide score distribution were used as filtering criteria for database searching [73, 75].

To identify differentially expressed proteins (DEPs) between the Vector and RPS27-RPS24 groups, we employed the R package limma. The criteria for differential expression were set as |log2FC | > 1 and *P* < 0.05. The identified DEPs were subjected to the STRING database (https://string-db.org/), with the species limited to “Homo sapiens”. A minimum required interaction score of ≥0.4 was set as the threshold to construct a protein-protein interaction network, which was visualized using Cytoscape software (v3.8.2) [76].

To investigate the functions of the DEPs further, we utilized the R package clusterProfiler and performed enrichment analysis for gene ontology (GO), Kyoto Encyclopedia of Genes and Genomes (KEGG), and WikiPathways. The significance threshold was set at *P* < 0.05. The GO terms included biological processes (BP), cellular components (CC), and molecular function (MF) [77].

### Metabolomic analysis

Metabolomic analysis was conducted using the LC20 ultra-high-performance liquid chromatography system (Shimadzu, Japan) coupled with a Triple TOF-6600 mass spectrometer (AB Sciex). U2OS/ADMR cell samples from the stable Vector group (*N* = 6) and RPS27-RPS24 group (*N* = 6) were targeted for analysis. Chromatographic separation was performed using a Waters ACQUITY UPLC HSS T3 C18 column (100 × 2.1 mm, 1.8 μm). The column temperature was maintained at 40 °C, and the flow rate was set at 0.4 mL/min. The mobile phase consisted of an acetonitrile-water solution containing 0.1% formic acid. The gradient elution program for mobile phase B was as follows: 5%, 0.0–11.0 min; 90%, 11.0–12.0 min; 5%, 12.1–14 min. The eluent was directly introduced to the mass spectrometer without splitting [78].

For positive/negative ion mode mass spectrometry, the conditions were set as follows: ionization voltage of 5500 V, capillary temperature of 550 °C, spray gas flow rate of 50 psi, and auxiliary heating gas flow rate of 60 psi. To prevent overfitting, the preprocessed data were analyzed using orthogonal partial least-squares discriminant analysis (OPLS-DA) and permutation tests (100 permutations). Metabolites with VIP scores > 1 and *P* < 0.05 in the OPLS-DA model were identified as differential metabolites (DMs). Additionally, differential metabolites were selected based on the combination of multivariate analysis with fold change ≥ 2 and ≤0.5 and univariate analysis with *P* < 0.05 in the Student’s *t*-test. Metabolic pathways related to the identified metabolites were determined using the MetaboAnalyst software (v5.0) [79].

### CRISPR/Cas9 editing technology

The sgRNA used to construct GLS-KO cells through CRISPR/Cas9 technology is as follows: GLS-sgRNA: F: 5’-TCCTTCAGCTCCTGCTG-3’ (PAM: AGG); R: 5’-TGACAATAATAGTTTCAACA-3’ (PAM: TGG). The sgRNA was inserted into the Lenti-CRISPR v2 vector (obtained from Hanheng Biotech, Shanghai, China) containing the Streptococcus pyogenes Cas9 nuclease gene. Cells were transduced with the lentiviral Lenti-CRISPR v2 vector and subsequently subjected to a CRISPR/Cas9 editing system to generate GLS-KO cells. Transfected cells with sgRNA plasmid and donor sequence were selected using 4 μg/mL puromycin. Surviving cells were screened by limiting dilution cloning and confirmed as GLS-KO cells through RT-qPCR and Western blot analysis [80].

### RT-qPCR

Total RNA was extracted using the Trizol reagent kit (T9424, Sigma-Aldrich, USA), and the quality and concentration of the RNA were assessed using UV–visible spectrophotometry (ND-1000, Nanodrop, Thermo Fisher, USA). Reverse transcription was performed using the PrimeScript™ RT-qPCR kit (RR086A, TaKaRa, Mountain View, CA, USA). Real-time quantitative reverse transcription polymerase chain reaction (RT-qPCR) was conducted using the SYBR Green PCR Master Mix (4364344, Applied Biosystems, USA) and ABI PRISM 7500 Sequence Detection System (Applied Biosystems, USA). GAPDH was used as an endogenous control for mRNA. Primer design was provided by Shanghai General Biotechnology Co., Ltd. (Table S2 for details). The fold change in expression of the target gene between the experimental and control groups was determined using the formula 2^−ΔΔCt^ (ΔΔCT = ΔCt experimental group−ΔCt control group, where ΔCt = target gene Ct−endogenous control Ct) [81].

### Western blot

First, tissues or cells were collected and lysed using an enhanced RIPA lysis buffer containing a protease inhibitor (P0013B, Beyotime, Shanghai, China). Protein concentration was determined using the BCA protein quantification kit (P0012, Beyotime, Shanghai, China). The proteins were separated by 10% SDS-PAGE and transferred onto a PVDF membrane. The membrane was then blocked with 5% BSA at room temperature for 2 h to prevent nonspecific binding. Diluted primary antibodies (Table S3 for details) were added and incubated at room temperature for 1 h. All primary antibodies were rabbit anti-human antibodies. After washing the membrane, HRP-conjugated secondary antibodies, goat anti-rabbit IgG (ab6721, 1:2000, Abcam, UK) or goat anti-mouse IgG (ab6785, 1:1000, Abcam, UK), were added and incubated at room temperature for 1 h. An equal amount of A and B reagents from Pierce™ ECL Western blot substrate (32209, Thermo Scientific™, Germany) were mixed and dropped onto the membrane, followed by exposure using a gel imaging system [82]. The Western blot images were captured using the Bio-Rad imaging system (BIO-RAD, USA), and the band intensities were quantified using Image J analysis software, with GAPDH as the endogenous control. Each experiment was repeated three times. The Full and uncropped western blots can be found at the supplementary materials.

### Cell grouping

The U2OS/ADMR and MG63/ADMR cell groups were organized as follows: (1) GLS-WT group (control cells), further divided into the Vector group (stable Vector transfected control cells), and the RPS27-RPS24 group (stable RPS27-RPS24 transfected control cells); (2) GLS-KO group (GLS knockout cells), further divided into the Vector group (stable Vector transfected GLS knockout cells) and the RPS27-RPS24 group (stable RPS27-RPS24 transfected GLS knockout cells); (3) GLS-KO + RPS27-RPS24 + PBS group (GLS knockout cells with stable RPS27-RPS24 transfection, along with the addition of PBS); (4) GLS-KO + RPS27-RPS24 + TTM group (GLS knockout cells with stable RPS27-RPS24 transfection, along with the addition of the copper death inhibitor tetrathiomolybdate, TTM); (5) RPS27-RPS24 + PBS group (stable RPS27-RPS24 transfected cells with the addition of PBS); (6) RPS27-RPS24 + CB-839 group (stable RPS27-RPS24 transfected cells with the addition of the glutamine metabolism inhibitor CB-839). CB-839 (catalog number: HY-12248, 1 μM, treated for 72 h) and TTM (catalog number: HY-128530, 20 μM, treated for 12 h) were purchased from Med Chem Express [83]. In addition, for copper death-related testing, we treated the above cell groups with different concentrations of the copper ion carrier elesclomol–Cu (ratio = 1:1) for 72 h [84]. Afterward, we used the CCK-8 assay to measure cell viability, the scratch test to assess cell migration, and the Transwell assay to evaluate cell invasion. The elesclomol compound (catalog number: HY-12040) was purchased from Med Chem Express.

### MTT and CCK-8 assays for cell viability

MTT Assay: The test cells were seeded in a 96-well cell culture plate at a density of 3–5 × 10^4^ cells/mL and incubated for 48 h. MTT solution (CT02, Sigma Aldrich, USA) was added to the cell suspension and incubated for 4 h. Then, dimethyl sulfoxide (DMSO) was added, and the mixture was shaken for 10 min. The absorbance (OD 490 nm) was measured using a spectrophotometer (Laspec, China). The resistance to ADM was determined as the half inhibitory concentration (IC50) of ADM [85].

CCK-8 Assay: Each cell group was digested and resuspended, and the cell density was adjusted to 1 × 10^5^ cells/mL. Next, 100 μL/well of the cell suspension was seeded in a 96-well plate for routine culture. After the cells adhered to the well surface, drug treatment was applied, followed by overnight incubation. On the first and fourth days after incubation, the cell viability was assessed using the instructions provided with the CCK-8 assay kit (C0041, Beyotime, Shanghai, China). In each assessment, 10 μL of CCK-8 detection solution was added to each well and incubated for 4 h in a cell culture incubator. Subsequently, the absorbance at 450 nm was measured using an enzyme-linked immunosorbent assay reader, and the cell survival rate was calculated [86].

### Clonal formation assay for evaluating the capacity of cell cloning

Cells in the exponential growth phase were selected for the experiment. Conventional digestion and passage were used to prepare cell suspension, ensuring a single-cell rate of more than 95%. Subsequently, the cells were counted and diluted to an appropriate concentration. Each plate was seeded with 5 mL of cell suspension to achieve a uniform distribution of cells in a 60 mm culture dish. Gentle shaking in a crosswise direction was performed to facilitate dispersion. The culture dishes were then incubated at 37 °C with 5% CO_2_ for 2–3 weeks. Once visible clones appeared in the dishes, the cultivation was terminated. After discarding the culture medium, the dishes were carefully washed twice with PBS and air-dried. Samples were fixed with methanol for 15 min, followed by removal of methanol and air-drying. Subsequently, the samples were stained with Giemsa solution for 10 minutes, gently rinsed with running water, and air-dried again. Finally, the clones were visually counted using naked eyes or a low-power microscope, ensuring a count of more than 10 clones. The clonal formation rate was calculated using the following formula: Clonal formation rate = (number of clones/number of seeded cells) × 100% [87].

### EdU assay for cell proliferation

The test cells were seeded in a 24-well plate, and the culture medium was supplemented with EdU (C10310-2, Guangzhou Libo Biological Technology Co., Ltd., Guangzhou) to achieve a concentration of 10 µmol/L. After incubating in the CO_2_ incubator for 2 h, the culture medium was aspirated, and the cells were fixed with a PBS solution containing 4% paraformaldehyde at room temperature for 15 minutes. Subsequently, the cells were washed twice with PBS containing 3% BSA. Then, PBS containing 0.5% Triton-100 was added to each well and incubated at room temperature for 20 min, followed by two washes with PBS containing 3% BSA. Each well was then treated with 100 µL of staining solution and incubated in the dark at room temperature for 30 minutes. Afterward, DAPI was added for nuclear staining for 5 minutes. The samples were finally mounted on slides, and 6–10 random fields were observed under a fluorescence microscope (BX63, Olympus, Japan). The number of positive cells in each field was recorded. The EdU labeling rate (%) was calculated as the number of positive cells divided by the sum of positive and negative cells multiplied by 100% [88]. Each experiment was repeated three times.

### Scratch experiment to assess cell migration ability

Uniform horizontal lines were drawn on the back of a 6six-well plate using a marker, with each line spaced 1 cm apart, crossing through the wells. Approximately 5 × 10^5^ cells were seeded in each well and cultured until they reached a confluence of 100%. Subsequently, scratches were made perpendicular to the plane of the cells along the lines on the cell layer on the back of the plate. After completing the scratches, the cells were washed three times with sterile PBS to remove non-adherent cells, making the gaps created by the scratches clearly visible. Fresh serum-free culture medium was then replaced, and the cells were placed in a 37 °C, 5% CO_2_ incubator for static culture. After 24 h, the width of the scratches was observed and measured under a microscope, and the results were recorded by taking photographs [89]. Finally, migration rate analysis was performed using Image J software.

### Transwell assay for cell invasion

Cell invasion was evaluated using Transwell chambers with 8 µm pore size (3391, Corning, USA). For the invasion assay, Matrigel (354277, BD Biosciences, USA) was added to each well according to the manufacturer’s instructions, and cells from each group (2 × 10^5^ cells) were added to the serum-free culture medium and placed in the upper chamber of the Transwell. After incubating at 37 °C for 24 h, cells that had migrated were stained with 0.5% crystal violet at room temperature for 20 min. Using a Nikon Eclipse Ci optical microscope (Nikon, Tokyo, Japan), five random areas (top, bottom, center, left, and right) were selected for observation and photography. The number of positively stained cells in the five fields presenting a purple color was then counted and subjected to statistical analysis [90].

### In vivo animal experiment

Male NOD/SCID mice aged 6–8 weeks and weighing between 20 and 30 g were purchased from Hunan Slacc Jingda Experimental Animals Co., Ltd., China. The mice were housed in specific pathogen-free (SPF) animal facilities with a humidity range of 60–65%, temperature maintained at 22–25 °C, and provided ad libitum access to food and water under a 12-h light–dark cycle. After one week of acclimatization, the mice’s health status was observed before initiating the experiment. All animal procedures followed the guidelines for the care and use of laboratory animals [91].

A total of 5 × 10^6^ pretreated, co-cultured U2OS/ADMR or MG-63/ADMR cells, stably transfected with Vector or RPS27-RPS24, were injected subcutaneously in the left axillary region of the mice. Tumor growth was monitored and photographed regularly. Tumor volume was measured every other day, and once the subcutaneous tumor reached a diameter of approximately 3 mm, the mice received intraperitoneal injections of ADM twice a week at a dose of 5 mg/kg. Two weeks after drug injection, the fluorescent signals of the implanted pretreated OS cell lines were analyzed using the CRi Maestro in vivo imaging system (Cambridge Research & Instrumentation, Massachusetts, USA) [92]. On day 28, after the drug injection, the mice were euthanized with CO_2_ inhalation, and the tumors were excised and weighed. The collected tumor tissue was used for Western blot and immunohistochemical analysis.

The mice were randomly divided into 16 groups with 6 mice in each group: (1) Vector + PBS + PBS group: U2OS/ADMR or MG63/ADMR cells transfected with Vector were injected, followed by PBS injections; (2) RPS27-RPS24 + PBS + PBS group: U2OS/ADMR or MG63/ADMR cells transfected with RPS27-RPS24 were injected, followed by PBS injections; (3) RPS27-RPS24 + CB-839 + PBS group: U2OS/ADMR or MG63/ADMR cells transfected with RPS27-RPS24 were injected, followed by CB-839 and PBS injections of equal volume; (4) RPS27-RPS24 + CB-839 + TTM group: U2OS/ADMR or MG63/ADMR cells transfected with RPS27-RPS24 were injected, followed by oral administration of CB-839 (200 mg/kg, twice daily) or TTM (0.2 mg/day, once daily) [83, 93]. In each group, mice were treated with PBS or ADM.

### Immunohistochemical staining

Immunohistochemical (IHC) staining is a technique used to detect the expression of the Ki67 protein in tumor tissue. The specific steps are as follows: Firstly, subcutaneous transplant tumor tissue from mice is fixed overnight with 4% paraformaldehyde. It is then dehydrated, embedded, and prepared into 4 μm-thick paraffin sections. Next, the sections are deparaffinized with xylene and hydrated with a gradient of alcohol (including anhydrous ethanol, 95% ethanol, and 75% ethanol, each for 3 min). Subsequently, the sections are boiled in 0.01 M citrate buffer for 15–20 min for antigen retrieval. After that, they are incubated with 3% H_2_O_2_ at room temperature for 30 min to inactivate endogenous peroxidase. Then, goat serum-blocking solution is added and left for 20 min to remove excess fluid. Subsequently, a primary antibody against Ki67 (ab16667, 1:200, Abcam, UK) is added and incubated at room temperature for 1 h, followed by washing with PBS. Next, the sections are incubated with a secondary antibody (ab150077, 1:1000, Abcam, UK) at 37 °C for 20 min, then washed with PBS and treated with streptavidin-peroxidase (SP) at 37 °C for 30 min, followed by PBS washing. Then, DAB (P0202, Shanghai, China) from the Bi Yun Tian company is dropped on the sections, and after 5–10 min of staining, the reaction is stopped by washing with water for 10 min. Subsequently, the sections are counterstained with hematoxylin (C0107, Shanghai, China) from the Bi Yun Tian company for 2 min, followed by differentiation with hydrochloric acid ethanol and a final water rinse for 10 min. The sections are then dehydrated with gradient alcohol (using xylene for transparency) and finally mounted with 2–3 drops of neutral resin. Finally, the results are observed, and the following statistical analysis is performed under a light microscope: five high-power fields are randomly selected from each section, with 100 cells observed in each field, and the ratio of Ki67-positive cells is calculated [94].

### Statistical software and data analysis methods

In our study, we utilized R language, version 4.2.1, for data analysis and implemented compilation through the RStudio integrated development environment, version 2022.12.0-353. For file processing, we employed Perl language, version 5.30.0. Additionally, GraphPad Prism software, version 8.0, was utilized.

Quantitative data were presented as mean ± standard deviation. For comparing two groups of data, an independent sample *t*-test was employed. One-way analysis of variance (ANOVA) was used to compare data among different groups, while two-way ANOVA was utilized to compare data among different time points of multiple groups. Bonferroni correction was employed for post hoc analysis. The significance level was set at *P* < 0.05 [95].

## Supplementary information

Full and uncropped western blots

Supplementary Materials

## Data Availability

The datasets generated and/or analyzed during the current study are available from the corresponding author on reasonable request.
