# Supplementary material for: Single-cell multi-omics elucidates the role of RPS27-RPS24 fusion gene in osteosarcoma chemoresistance and metabolic regulation
Source: Cell Death Discov. 2025 Apr 25;11:197. doi: 10.1038/s41420-025-02487-9 (PMC12032165; doi:10.1038/s41420-025-02487-9)
Supplement: Supplementary file 1 — Full and uncropped western blots [file 41420_2025_2487_MOESM1_ESM.docx]

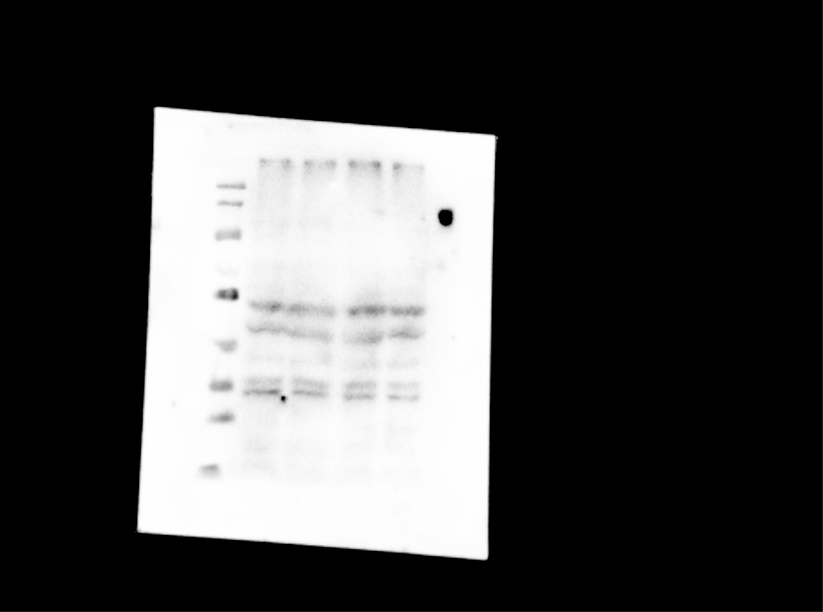


Full and uncropped western blots for figure 3A-1-1


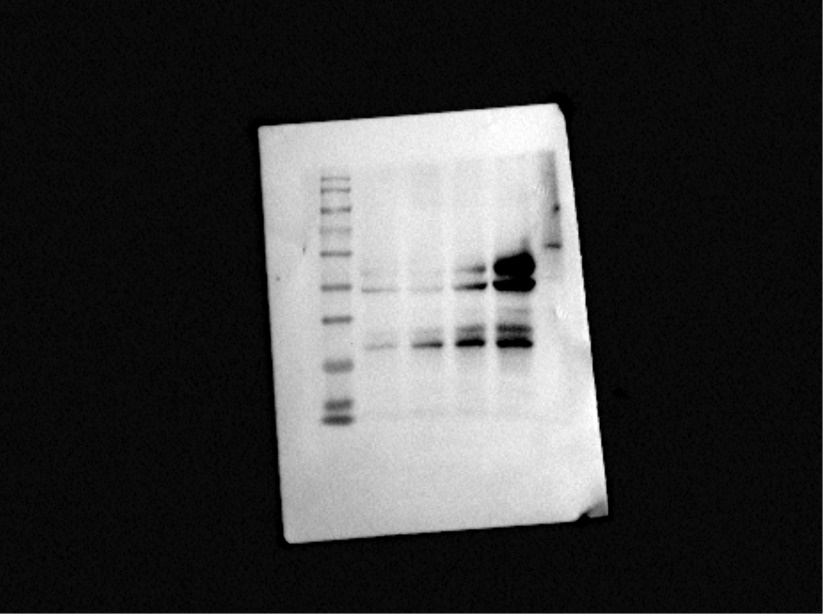


Full and uncropped western blots for figure 3A-1-2


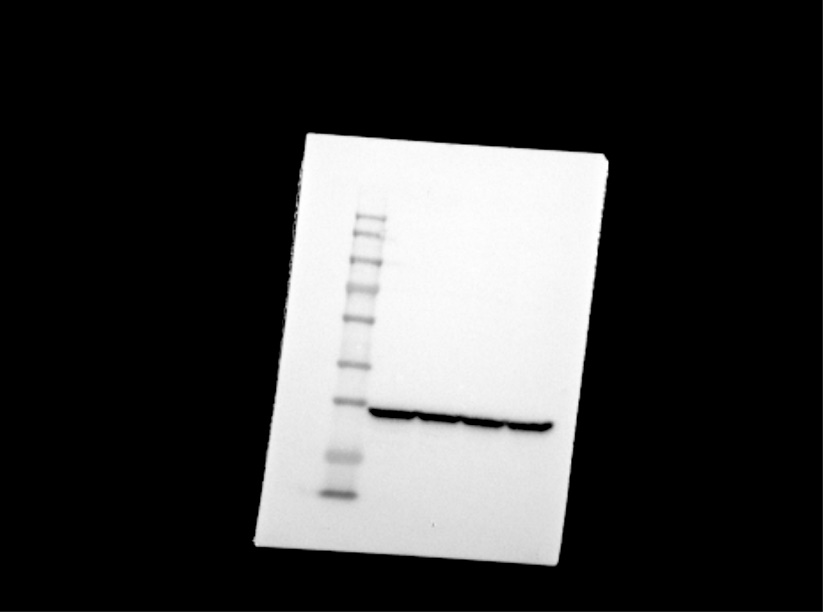


Full and uncropped western blots for figure 3A-2-1


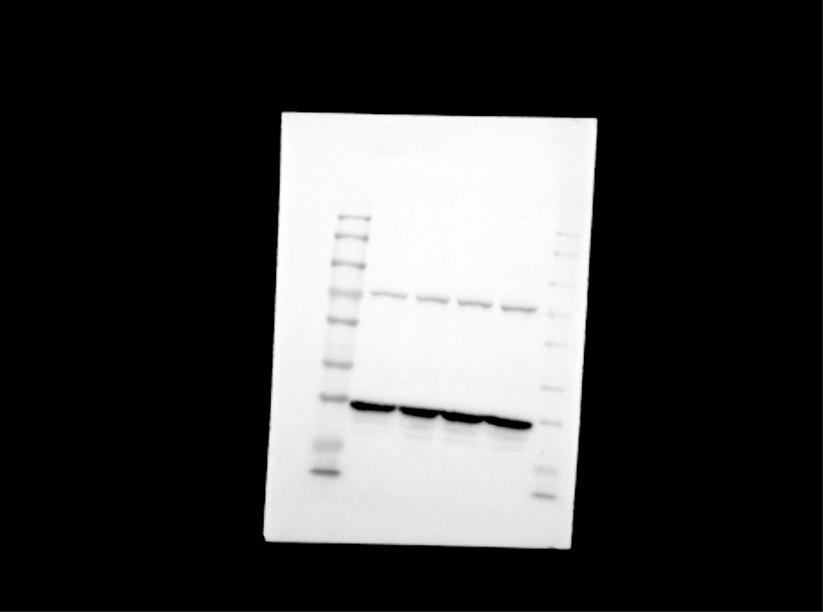


Full and uncropped western blots for figure 3A-2-2


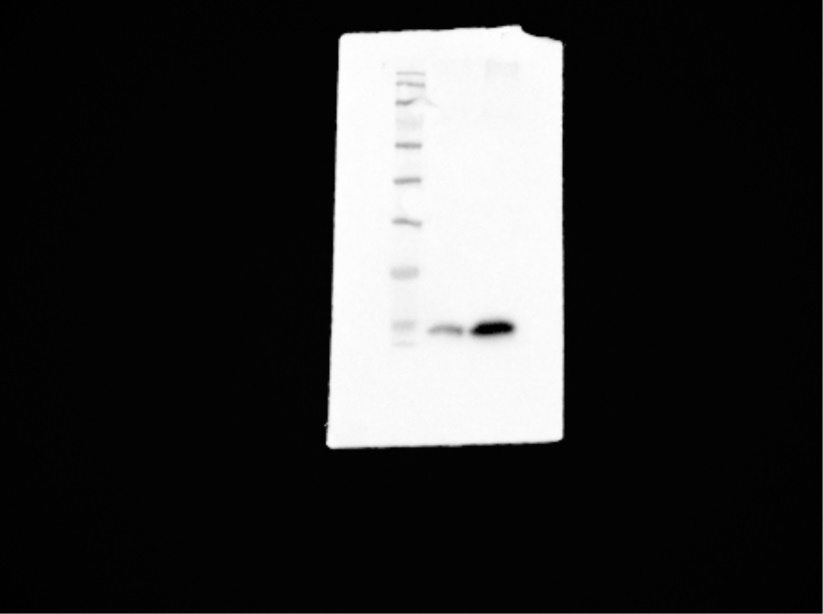


Full and uncropped western blots for figure 3B-1-1


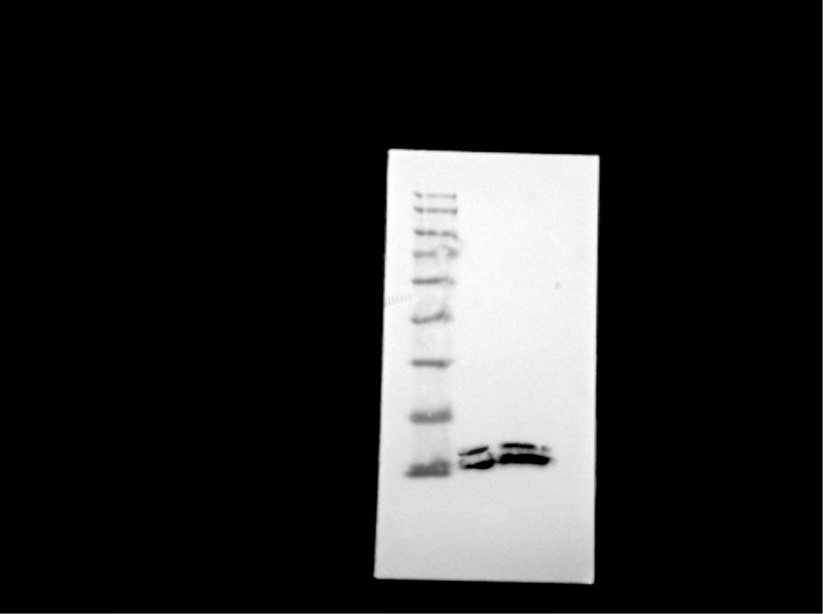


Full and uncropped western blots for figure 3B-1-2


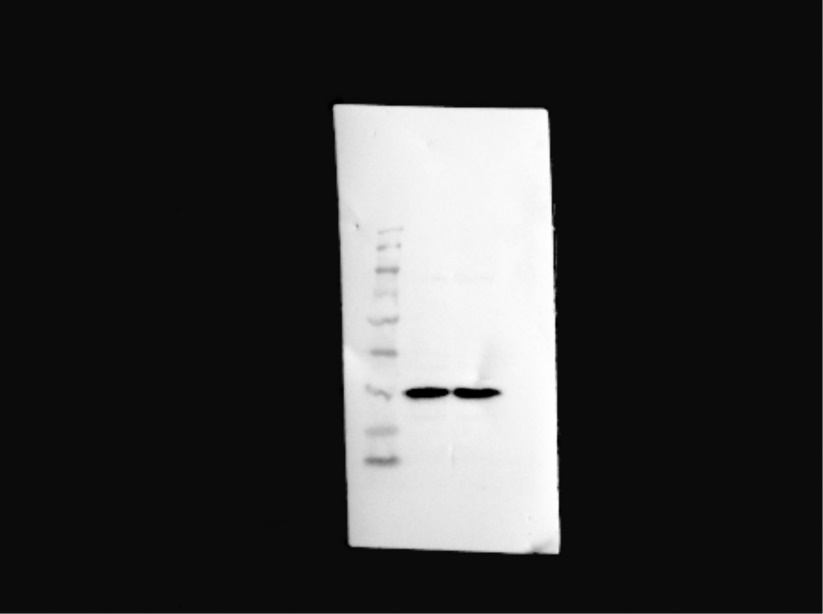


Full and uncropped western blots for figure 3B-2-1


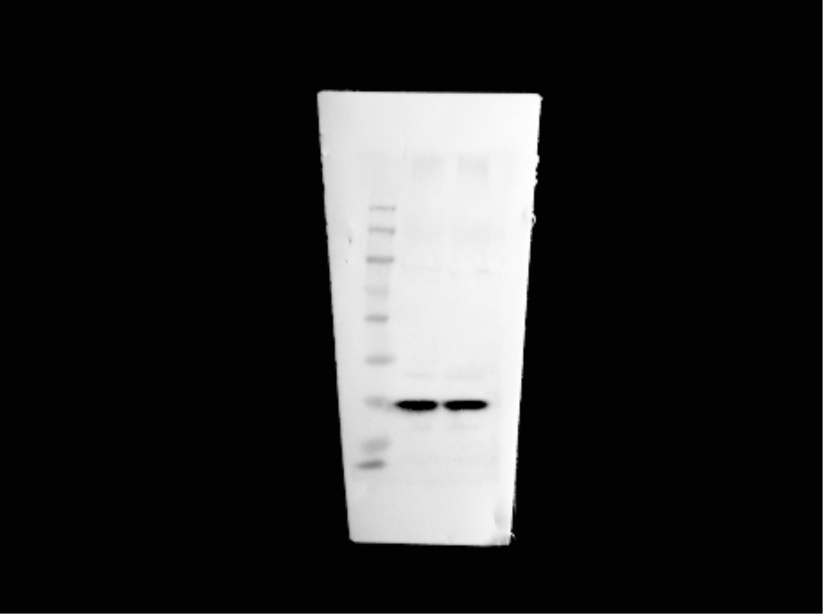


Full and uncropped western blots for figure 3B-2-2


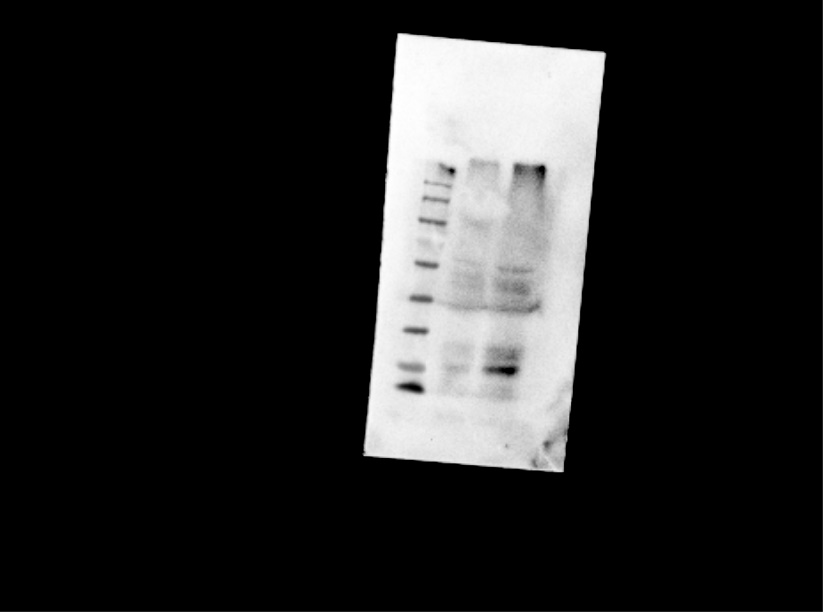


Full and uncropped western blots for figure 3C-1-1


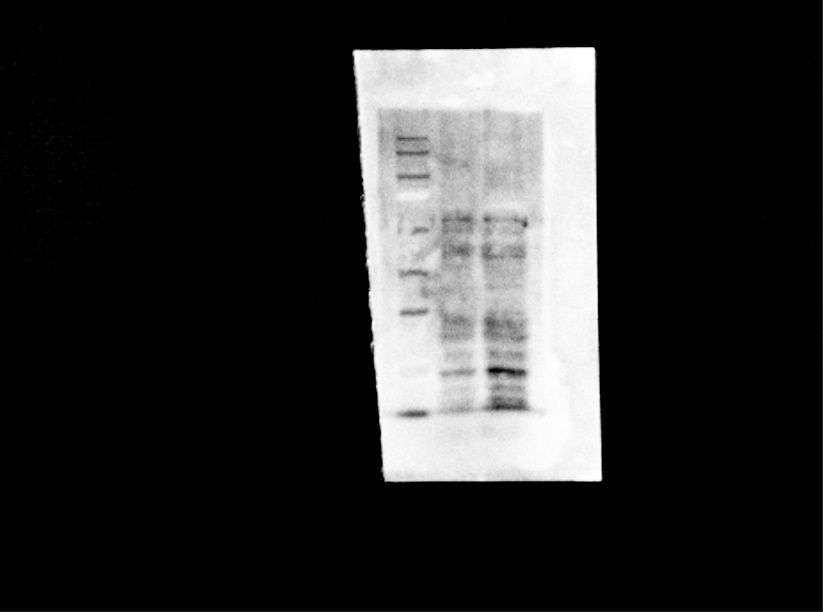


Full and uncropped western blots for figure 3C-1-2


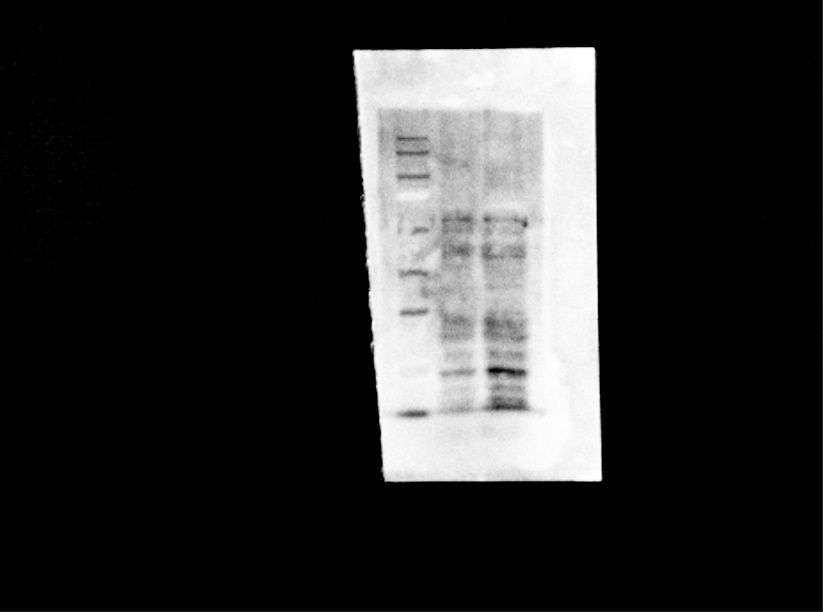


Full and uncropped western blots for figure 3C-2-1


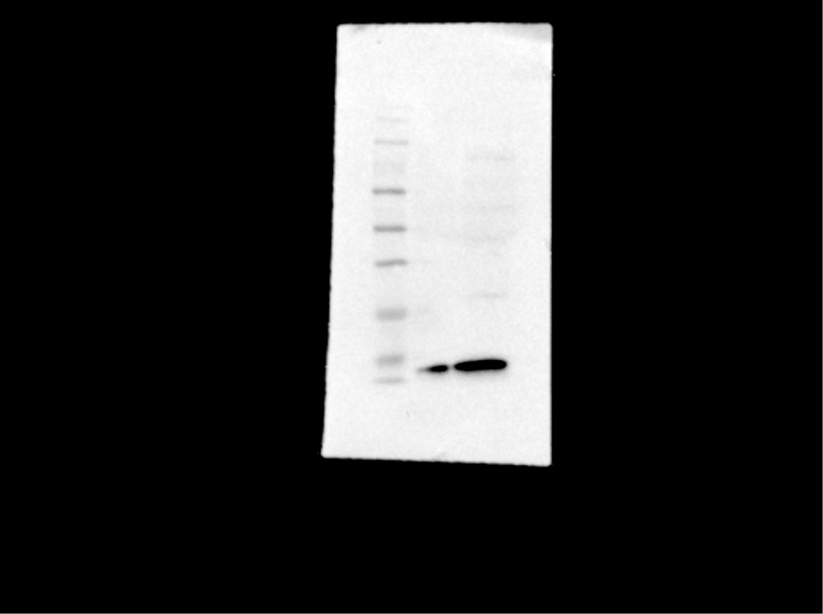

Full and uncropped western blots for figure 3C-2-2


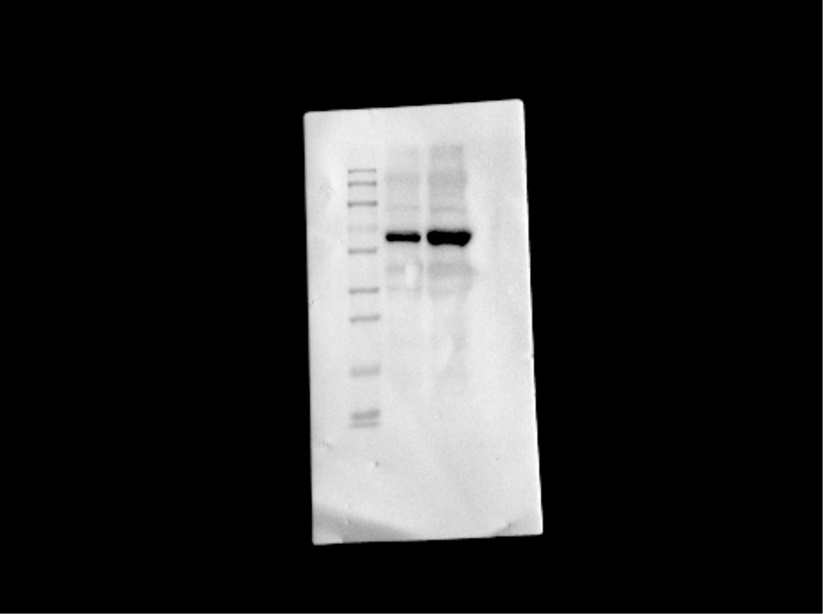


Full and uncropped western blots for figure 4H-1-1


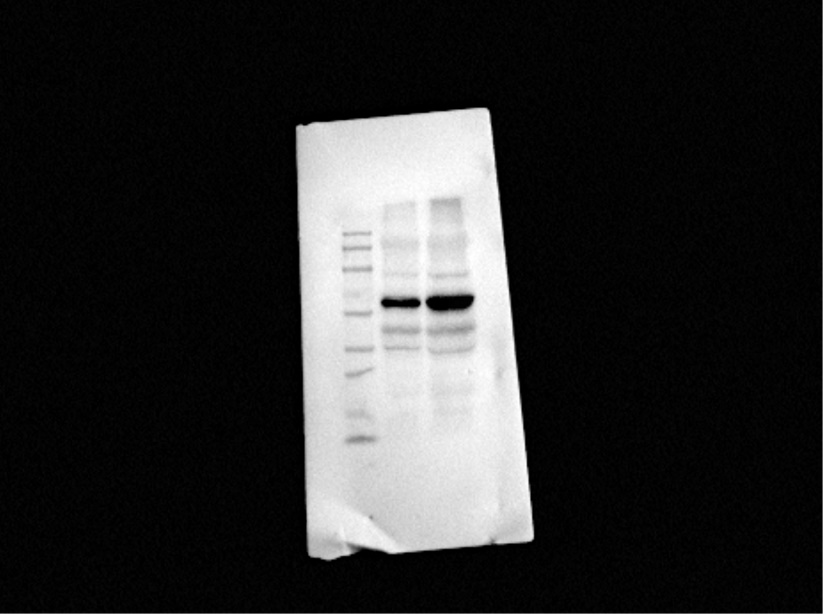


Full and uncropped western blots for figure 4H-1-2


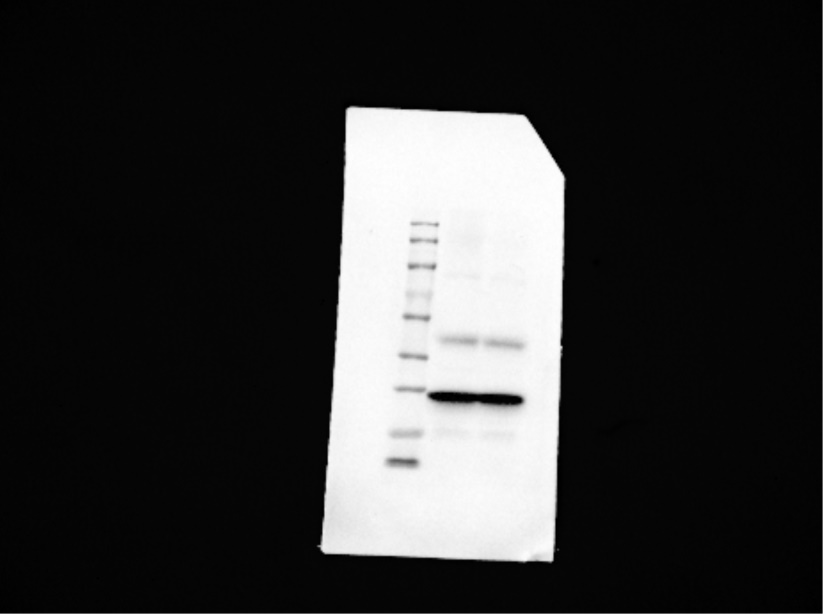


Full and uncropped western blots for figure 4H-2-1


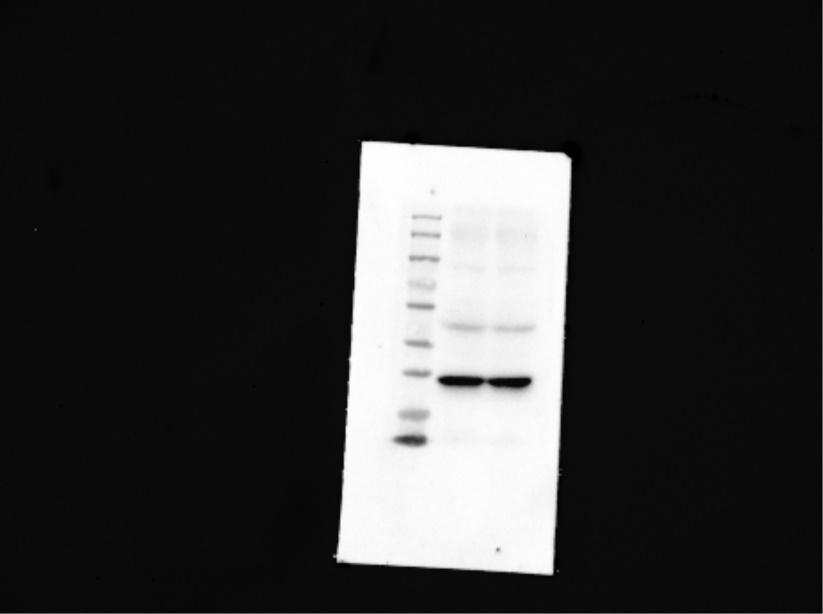


Full and uncropped western blots for figure 4H-2-2


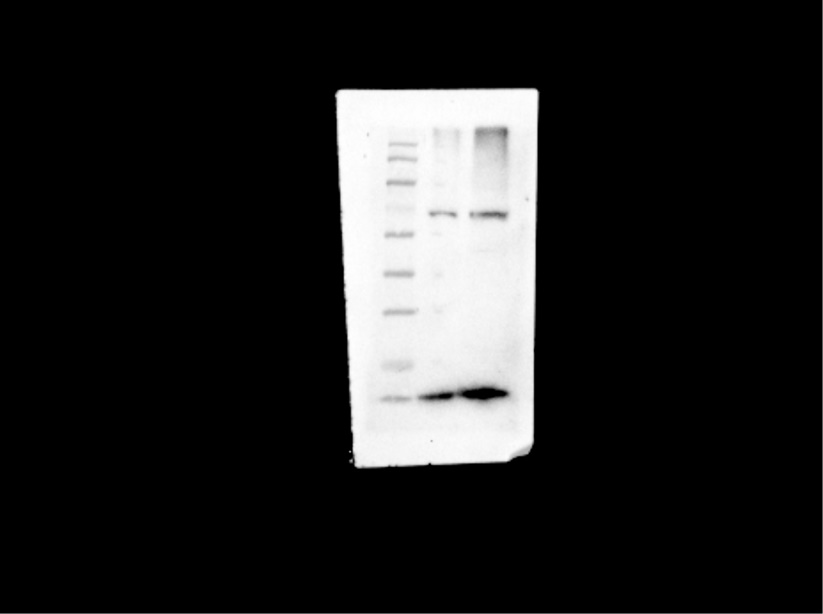


Full and uncropped western blots for figure 4I-1-1


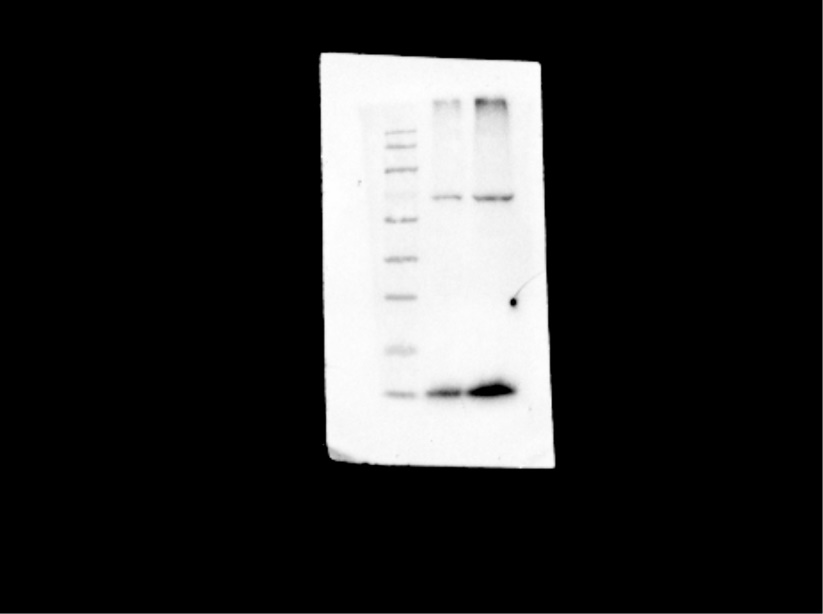


Full and uncropped western blots for figure 4I-1-2


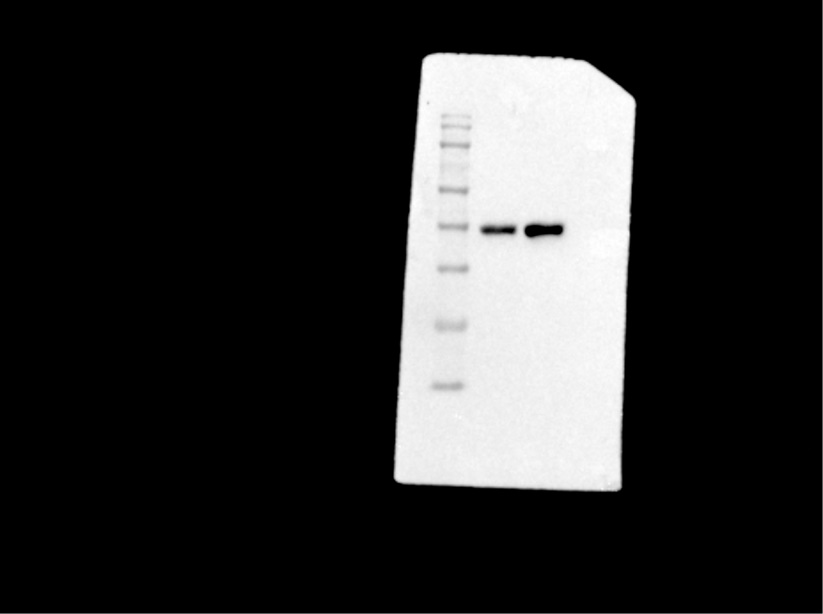


Full and uncropped western blots for figure 4I-2-1


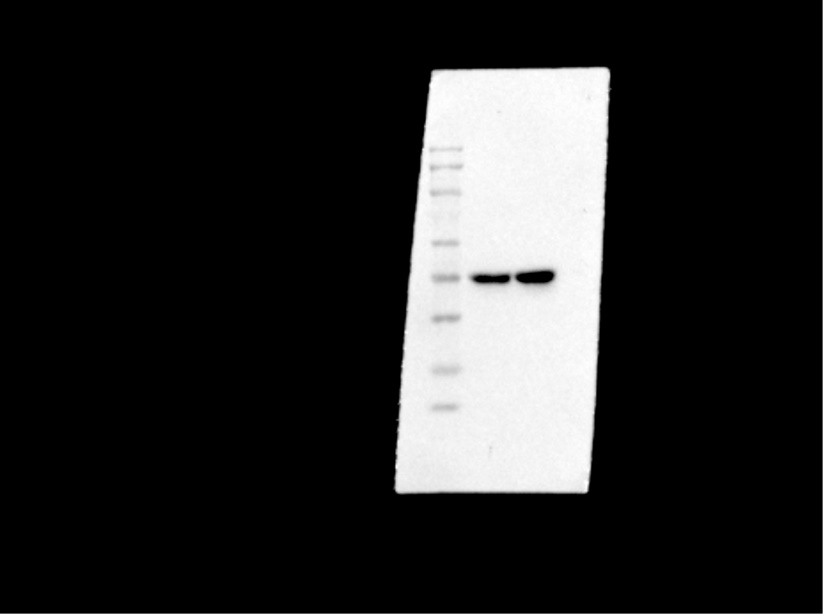


Full and uncropped western blots for figure 4I-2-2


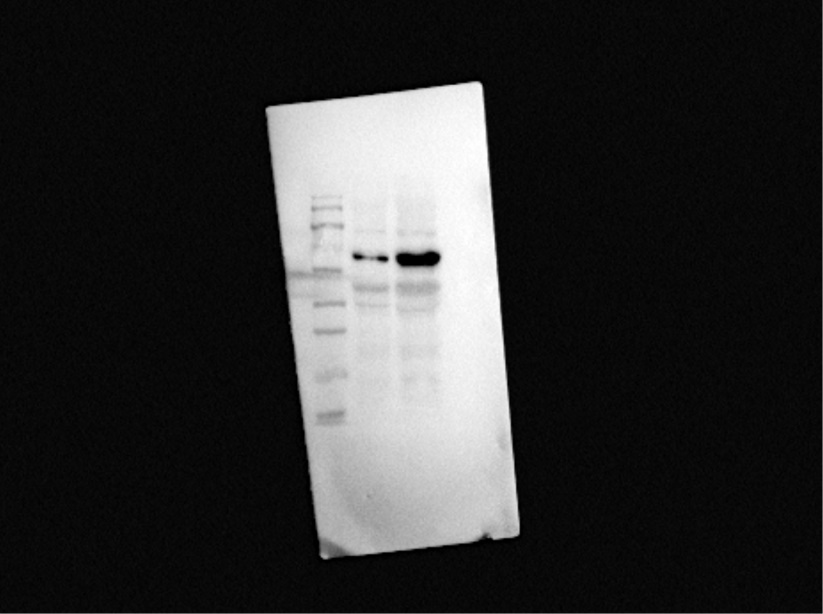


Full and uncropped western blots for figure 4I-3-1


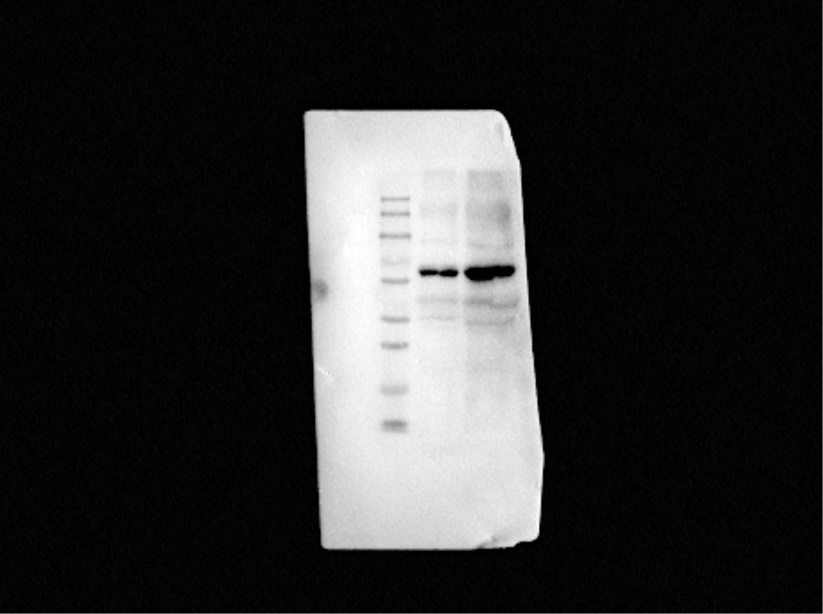


Full and uncropped western blots for figure 4I-3-2


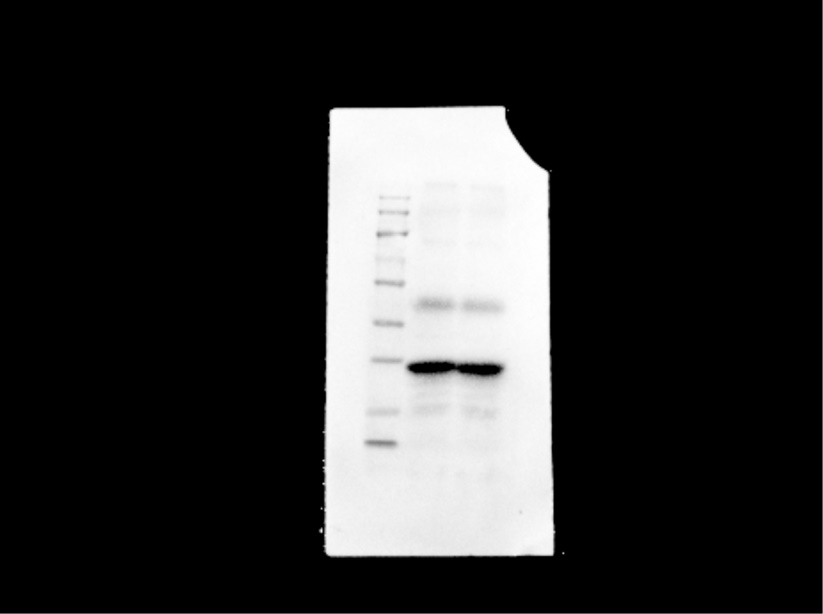


Full and uncropped western blots for figure 4I-4-1


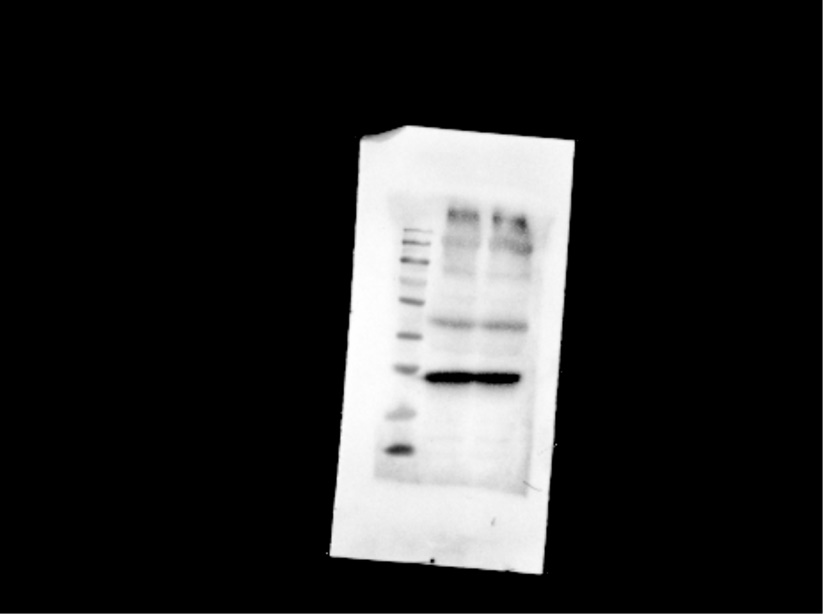


Full and uncropped western blots for figure 4I-4-2


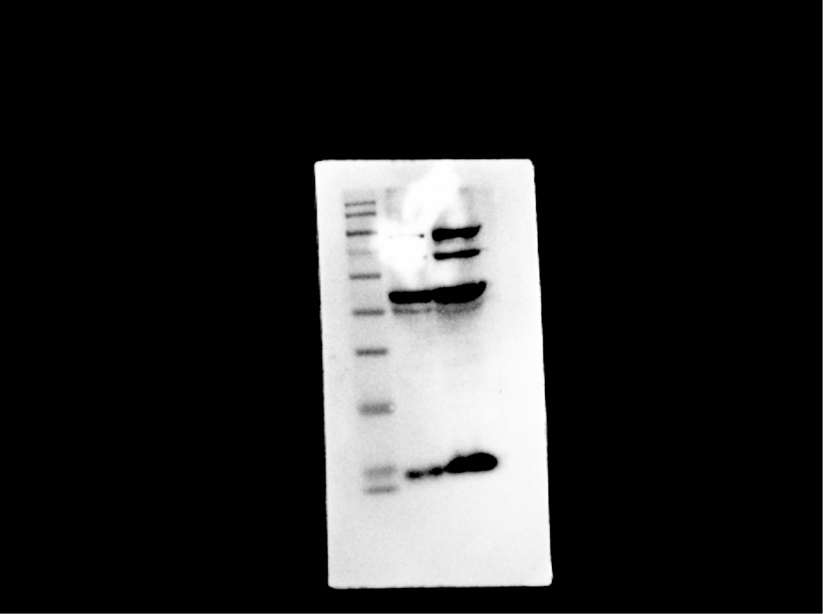


Full and uncropped western blots for figure 6E-1-1


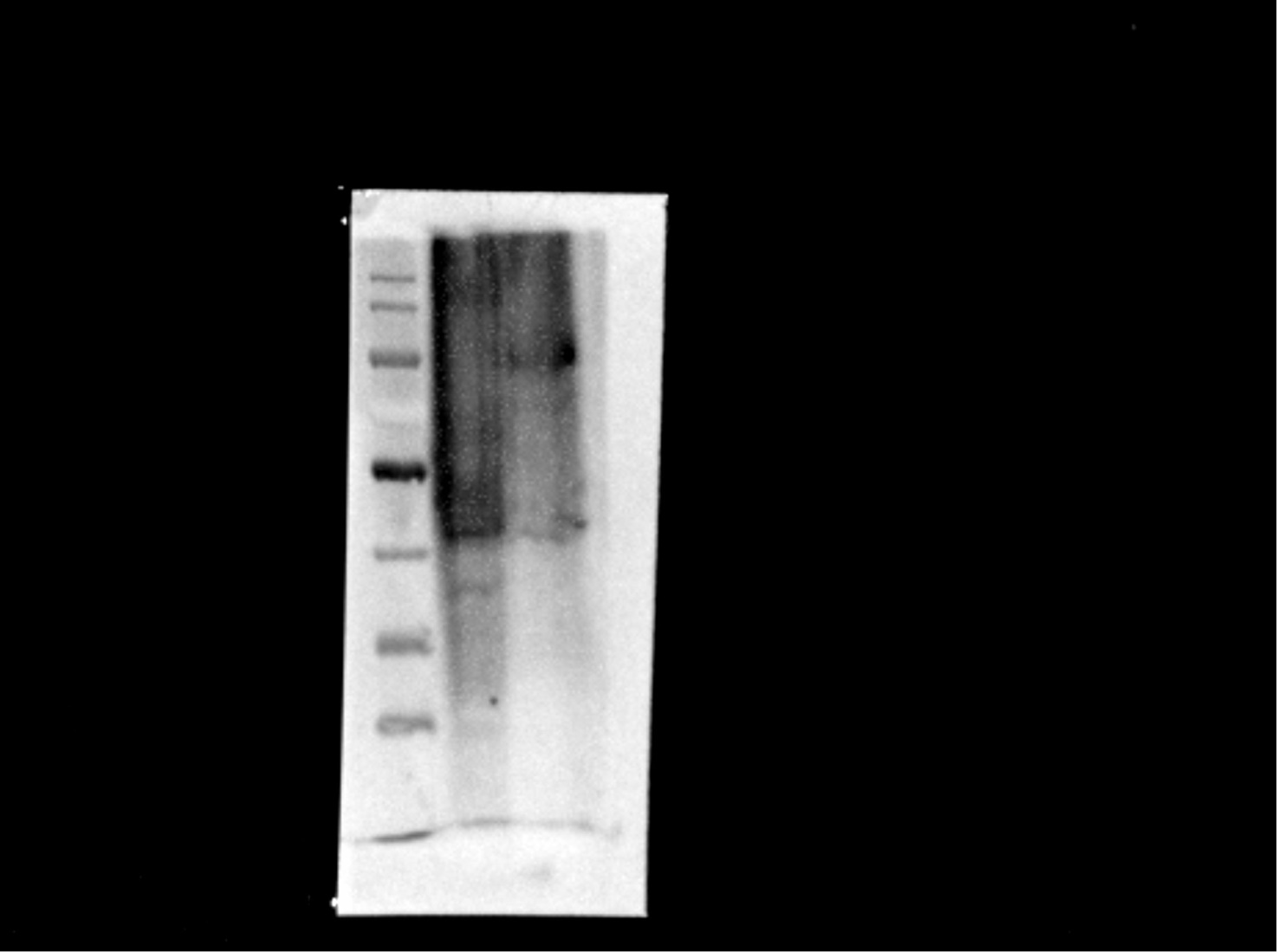


Full and uncropped western blots for figure 6E-1-2


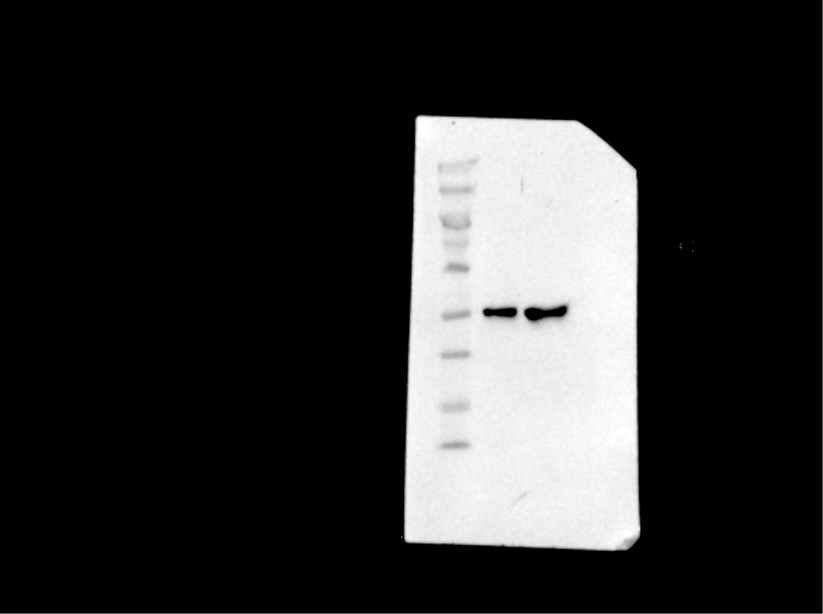


Full and uncropped western blots for figure 6E-2-1


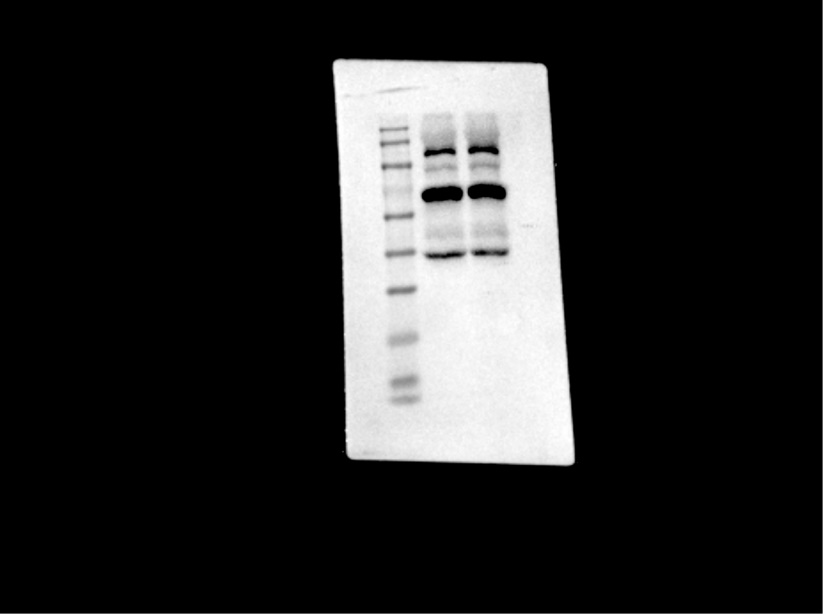


Full and uncropped western blots for figure 6E-2-2


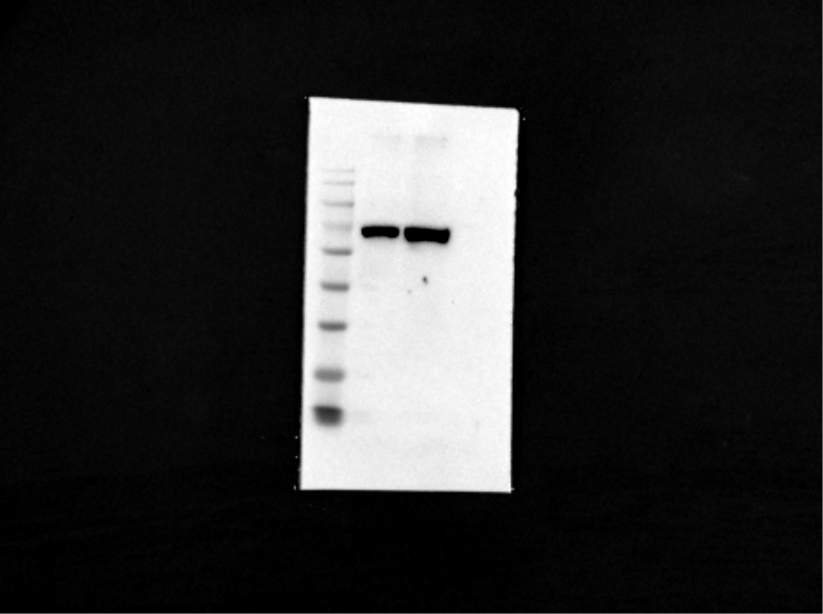


Full and uncropped western blots for figure 6E-3-1


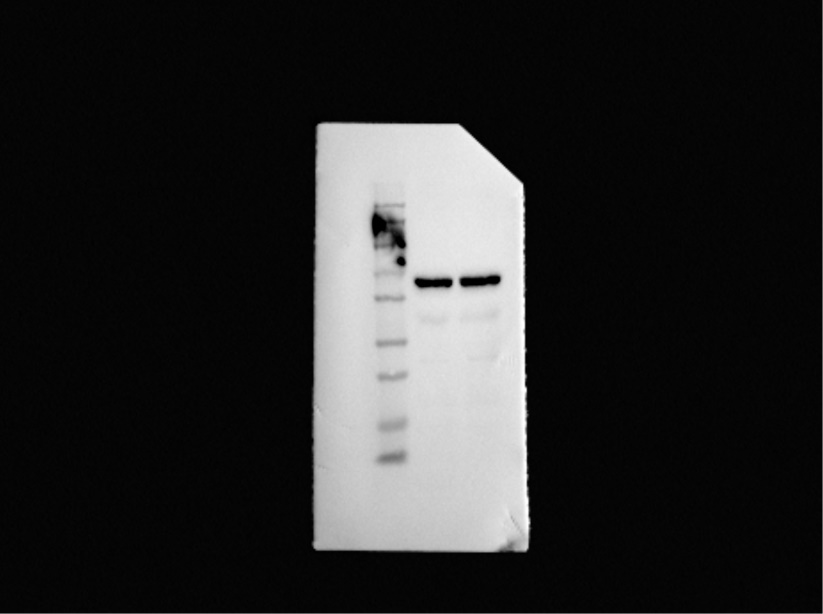


Full and uncropped western blots for figure 6E-3-2


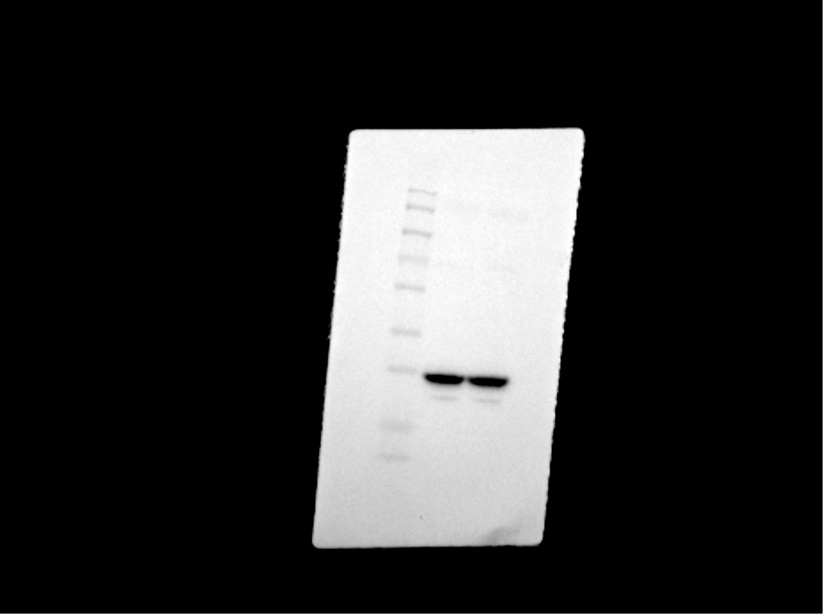


Full and uncropped western blots for figure 6E-4 (1)


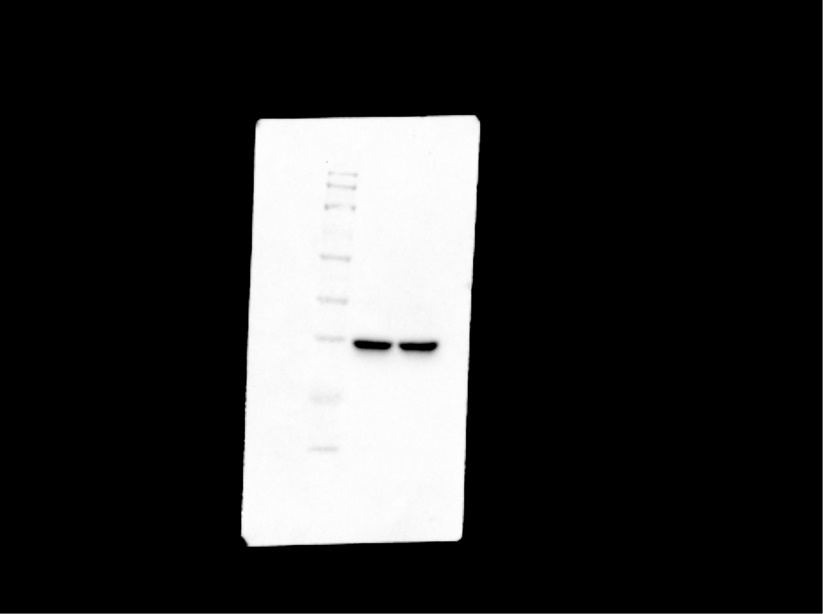


Full and uncropped western blots for figure 6E-4 (2)


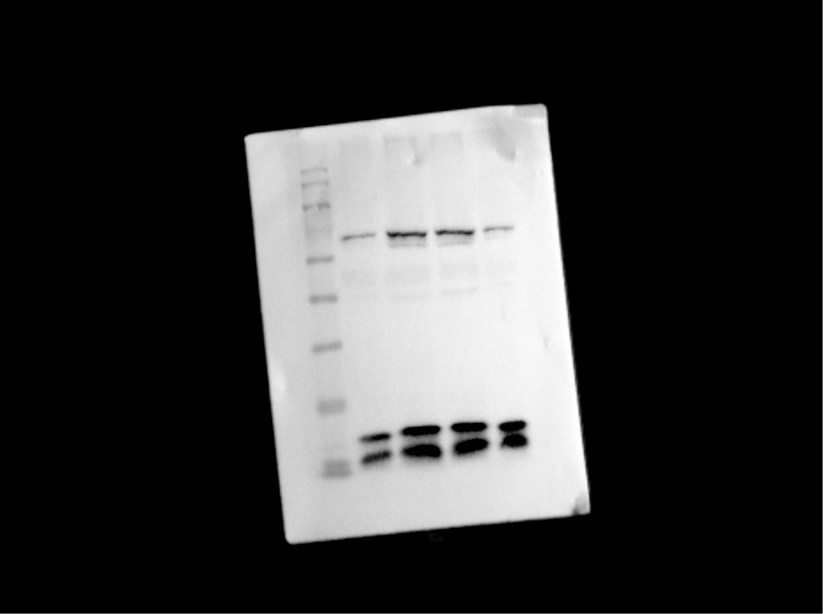


Full and uncropped western blots for figure 7B-1


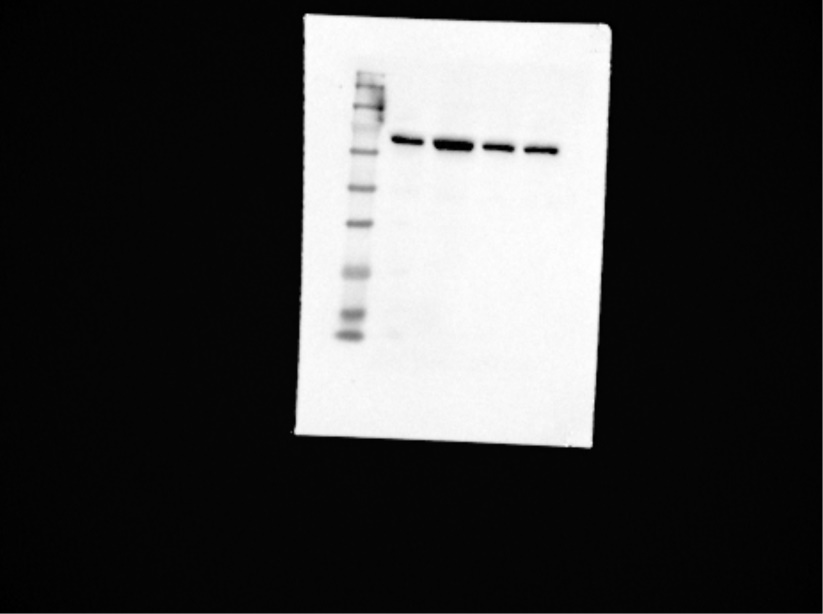


Full and uncropped western blots for figure 7B-2


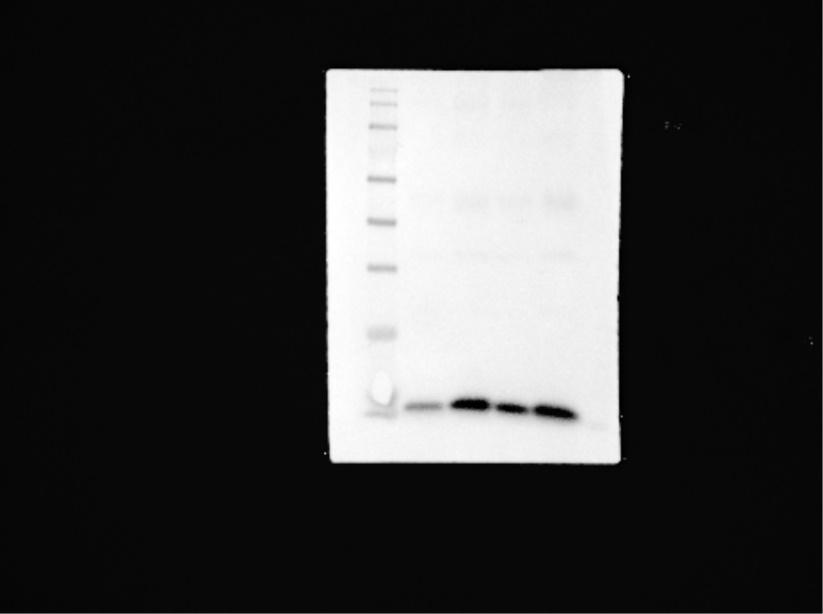


Full and uncropped western blots for figure 7B-3


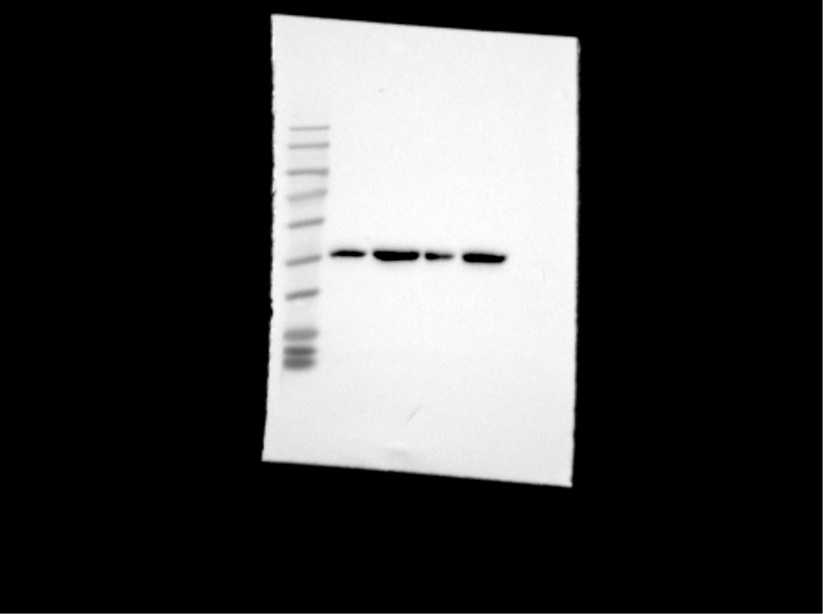


Full and uncropped western blots for figure 7B-4


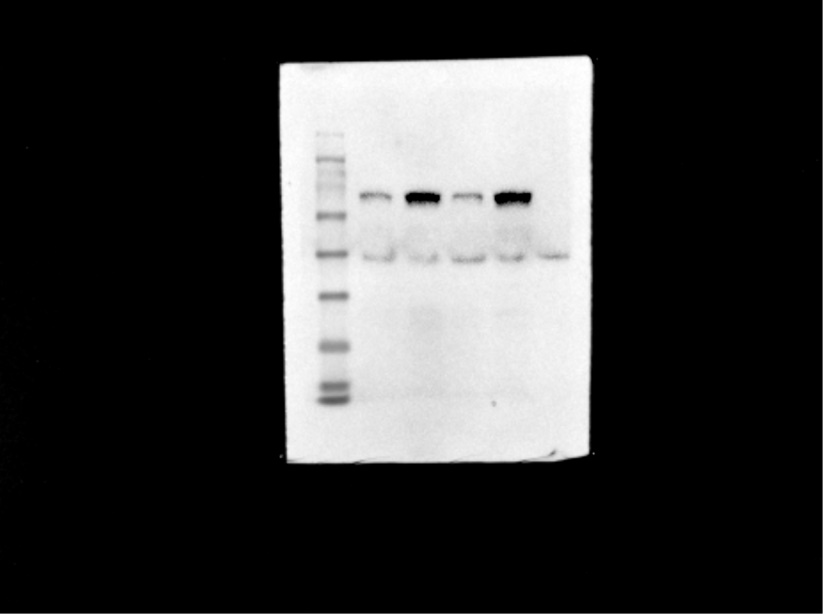


Full and uncropped western blots for figure 7B-5


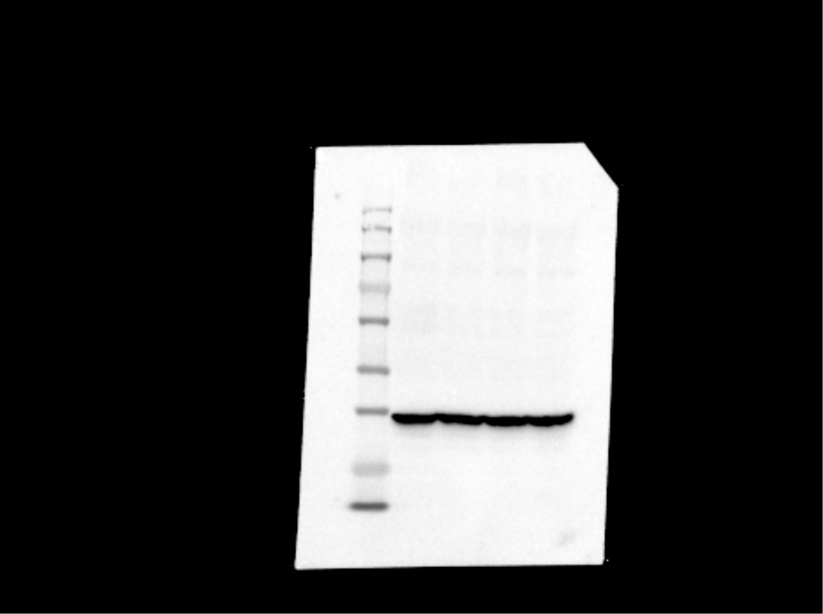


Full and uncropped western blots for figure 7B-6


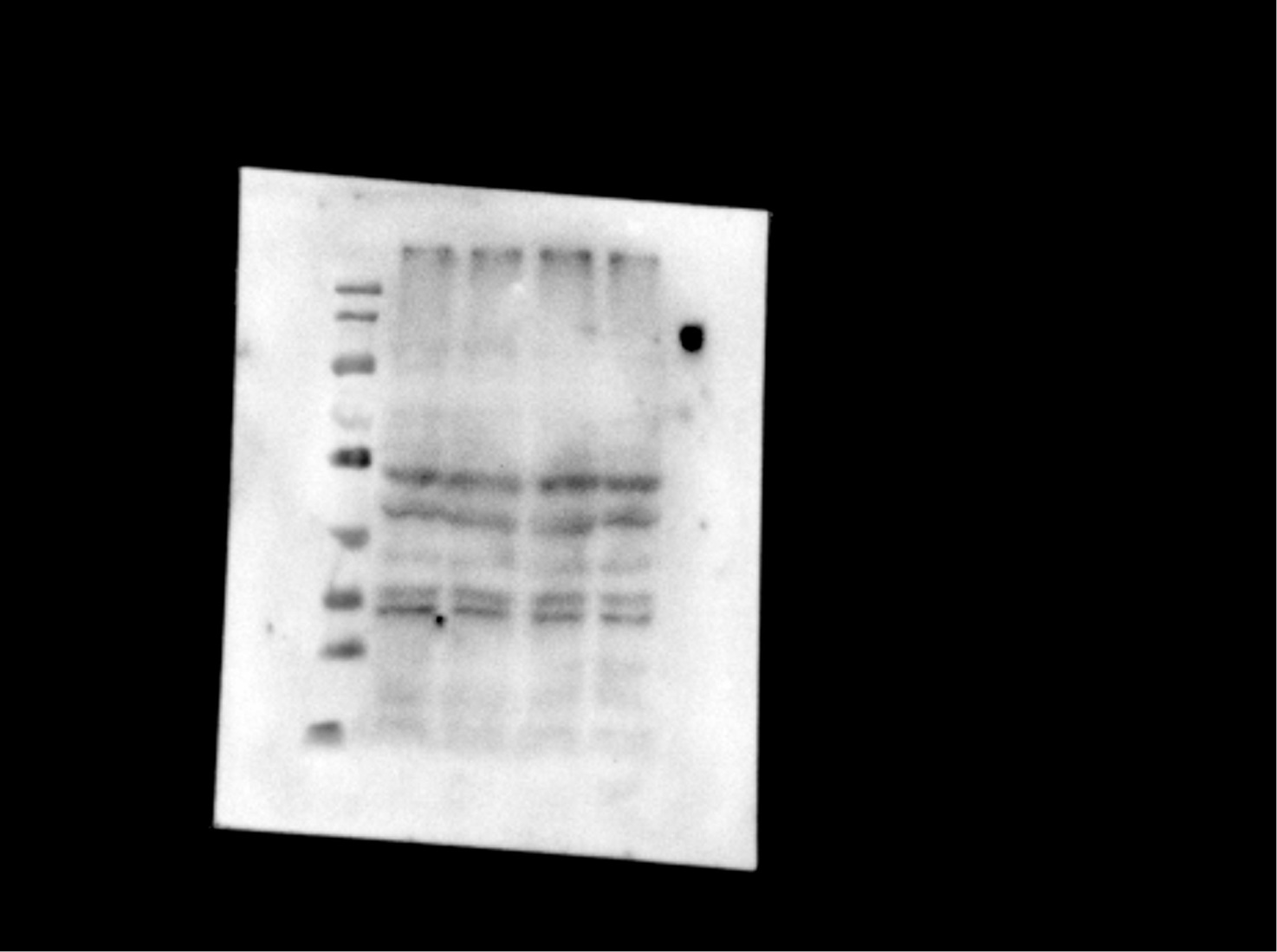


Full and uncropped western blots for figure S3A-1-1


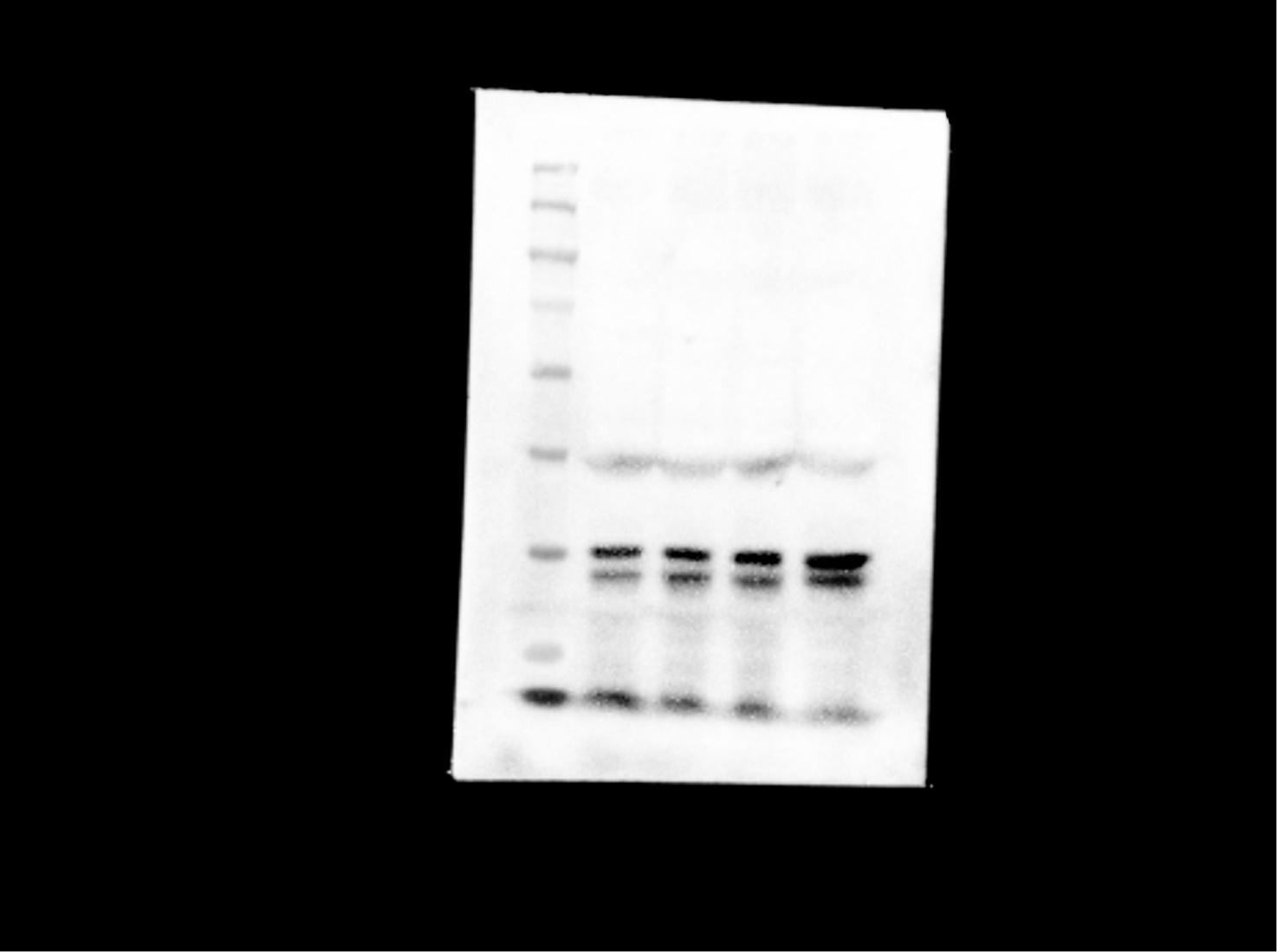


Full and uncropped western blots for figure S3A-1-2


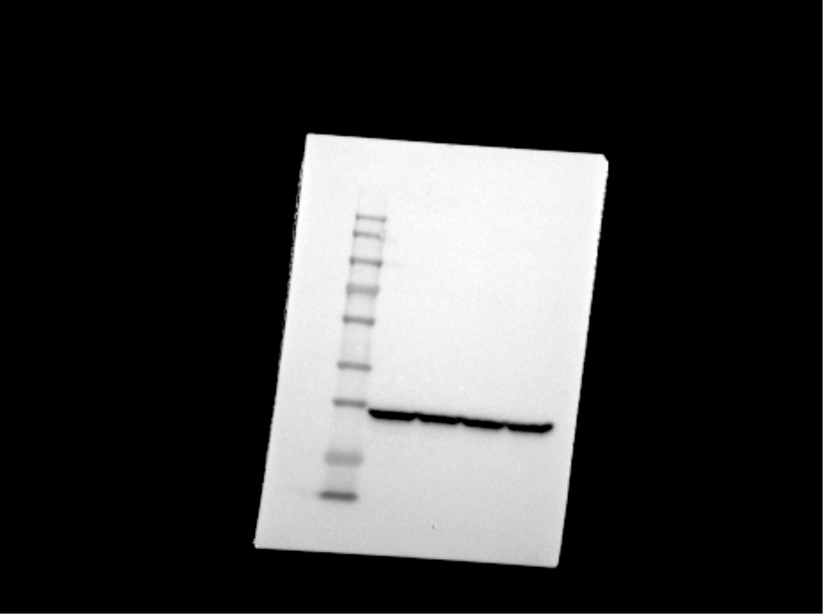


Full and uncropped western blots for figure S3A-2 (1)


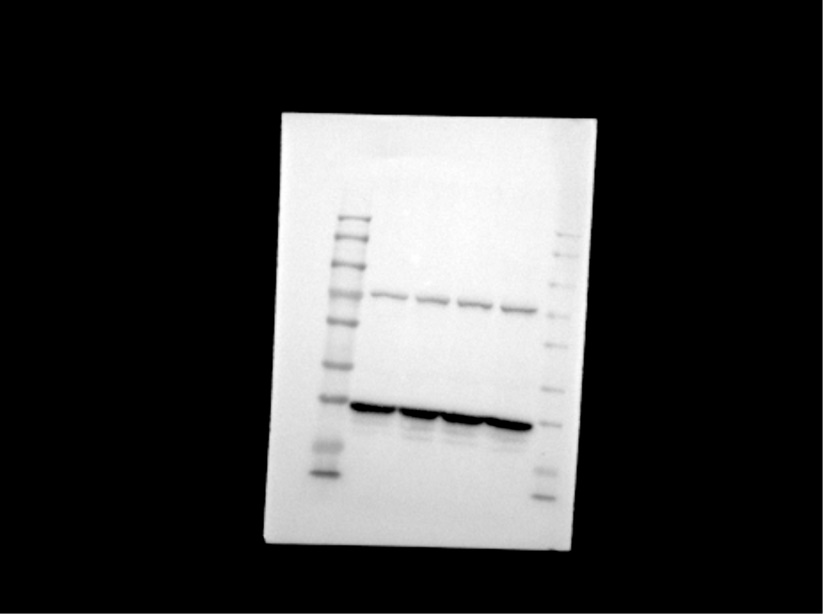


Full and uncropped western blots for figure S3A-2 (2)


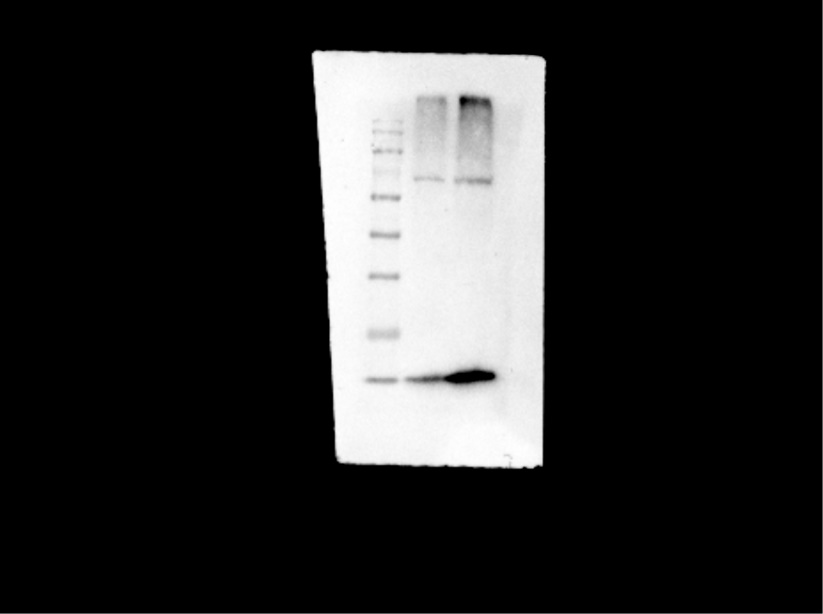


Full and uncropped western blots for figure S3B-1-1


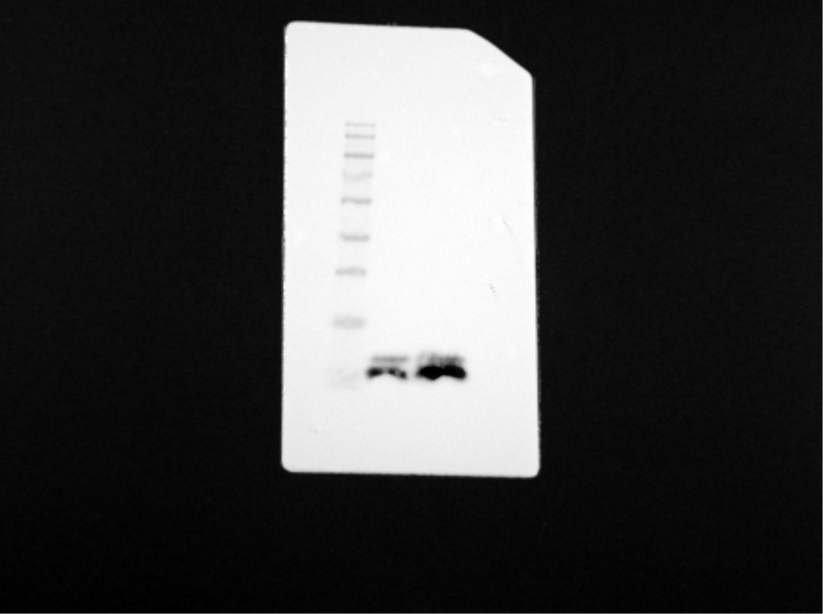


Full and uncropped western blots for figure S3B-1-2


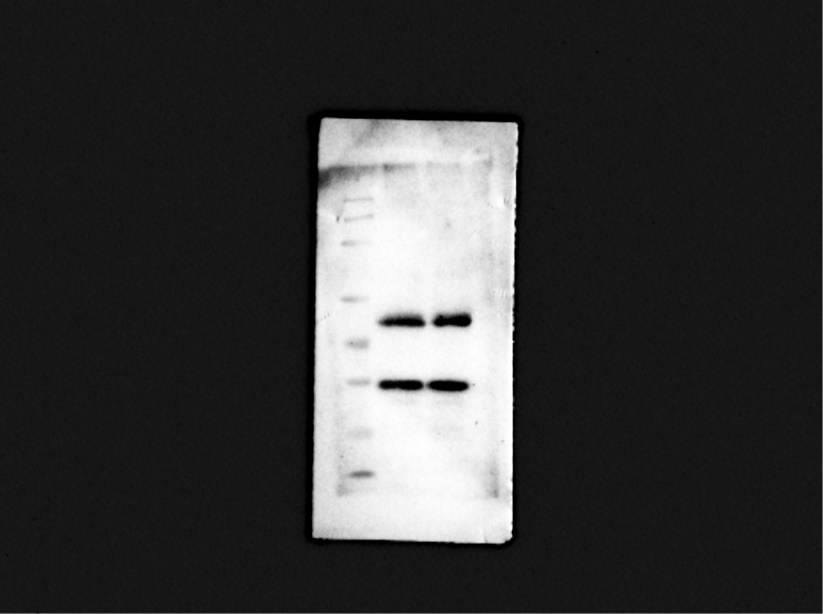


Full and uncropped western blots for figure S3B-2-1


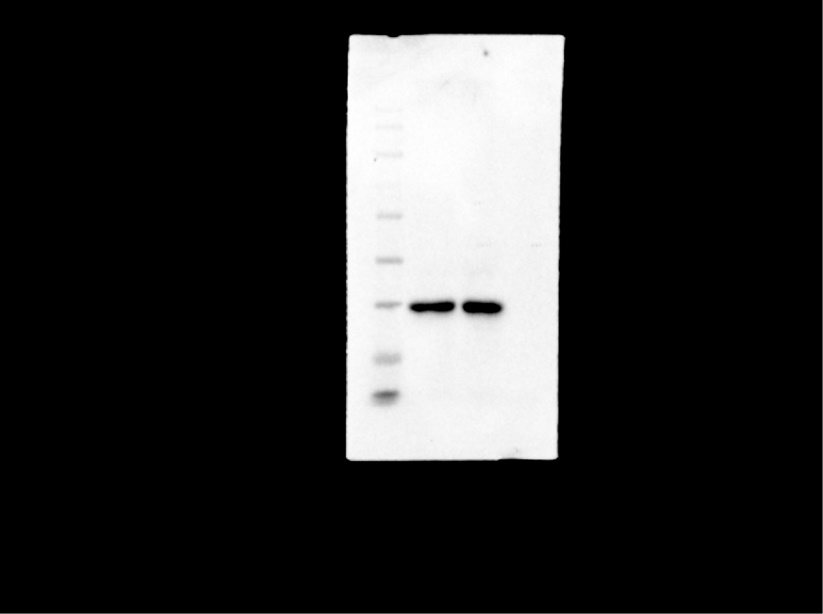


Full and uncropped western blots for figure S3B-2-2


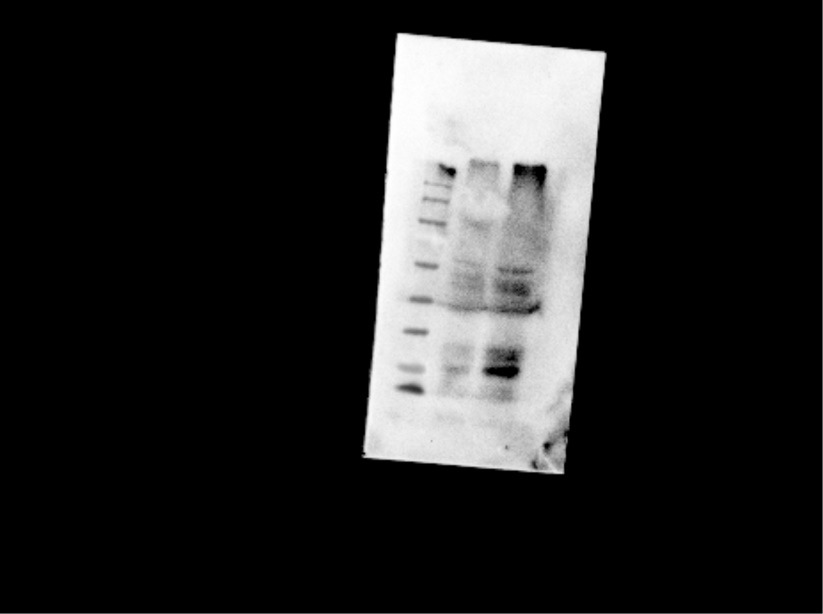


Full and uncropped western blots for figure S3C-1-1


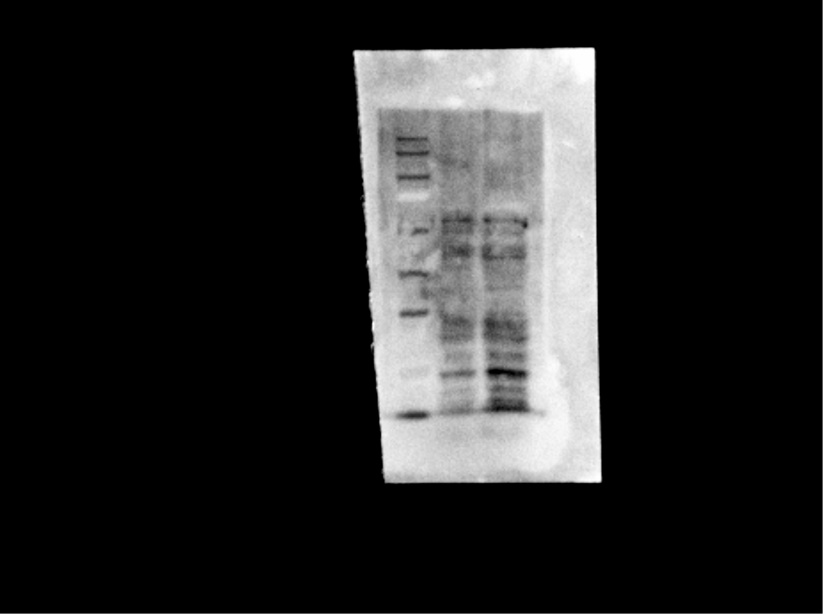


Full and uncropped western blots for figure S3C-1-2


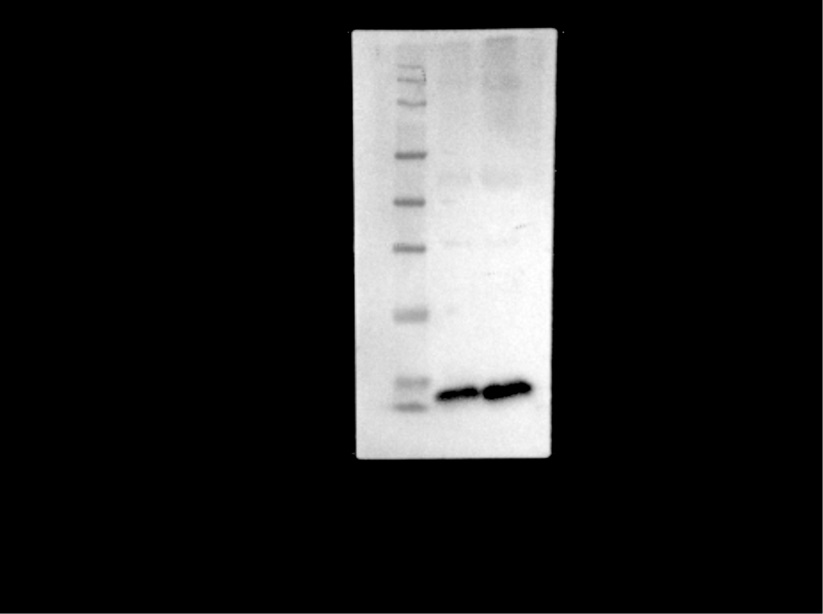


Full and uncropped western blots for figure S3C-2-1


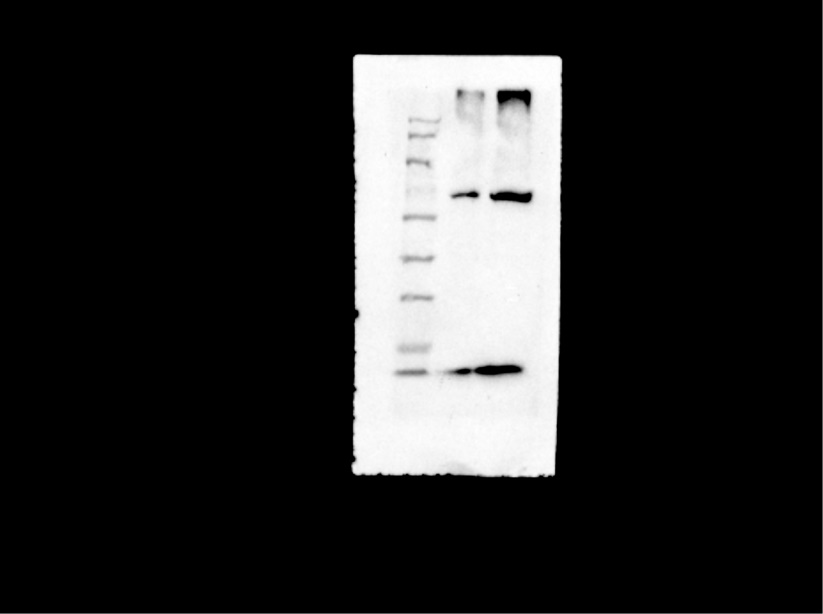


Full and uncropped western blots for figure S3C-2-2


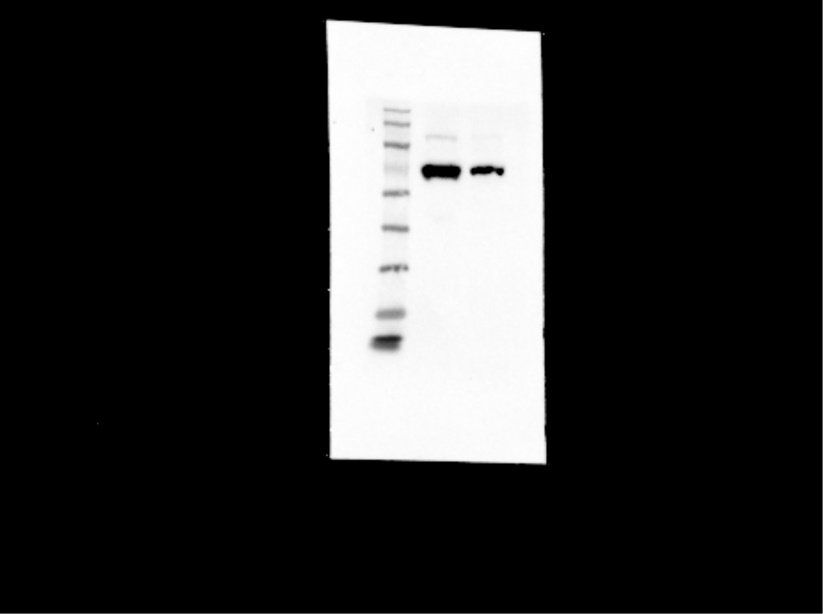


Full and uncropped western blots for figure S5C-1-1


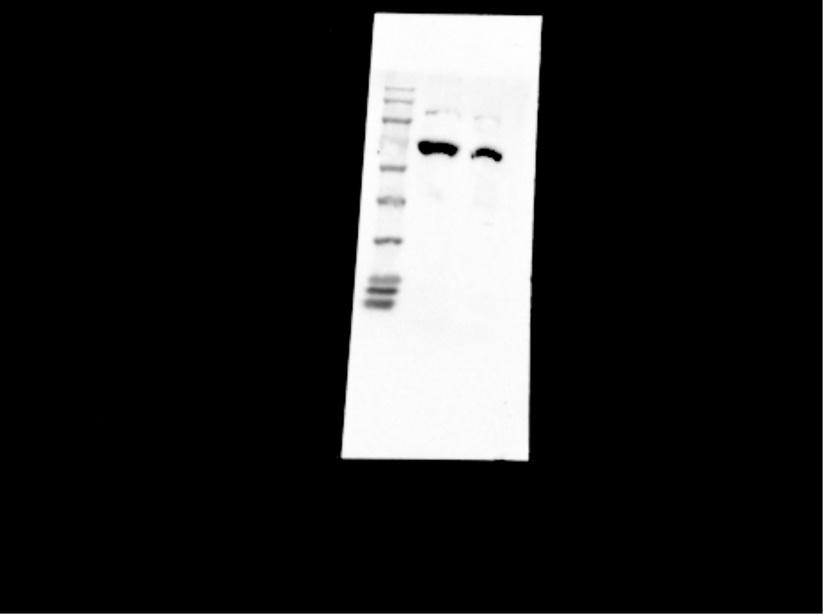


Full and uncropped western blots for figure S5C-1-2


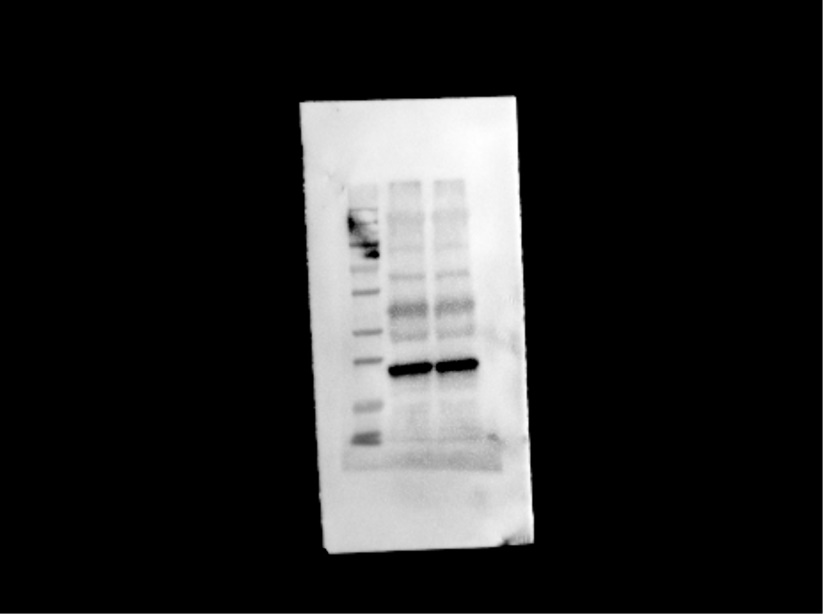


Full and uncropped western blots for figure S5C-2-1


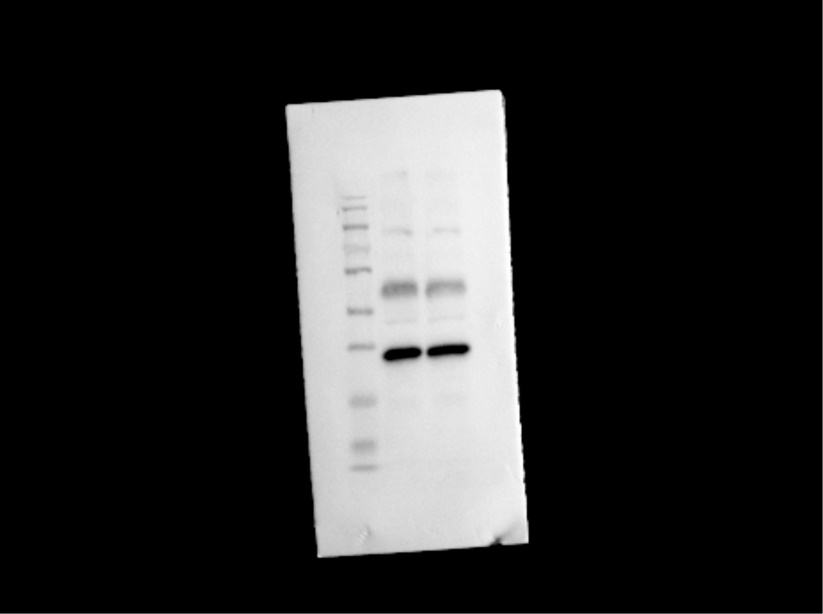


Full and uncropped western blots for figure S5C-2-2


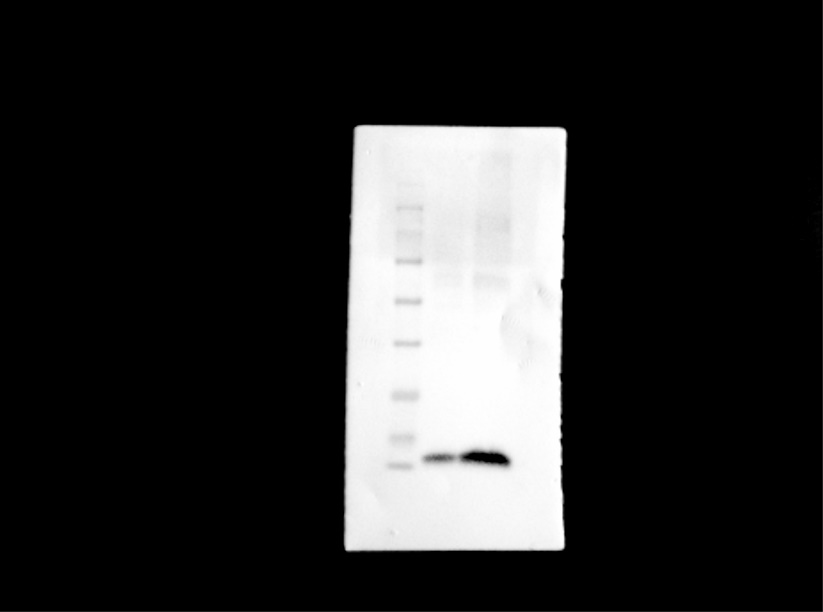


Full and uncropped western blots for figure S7D-1-1


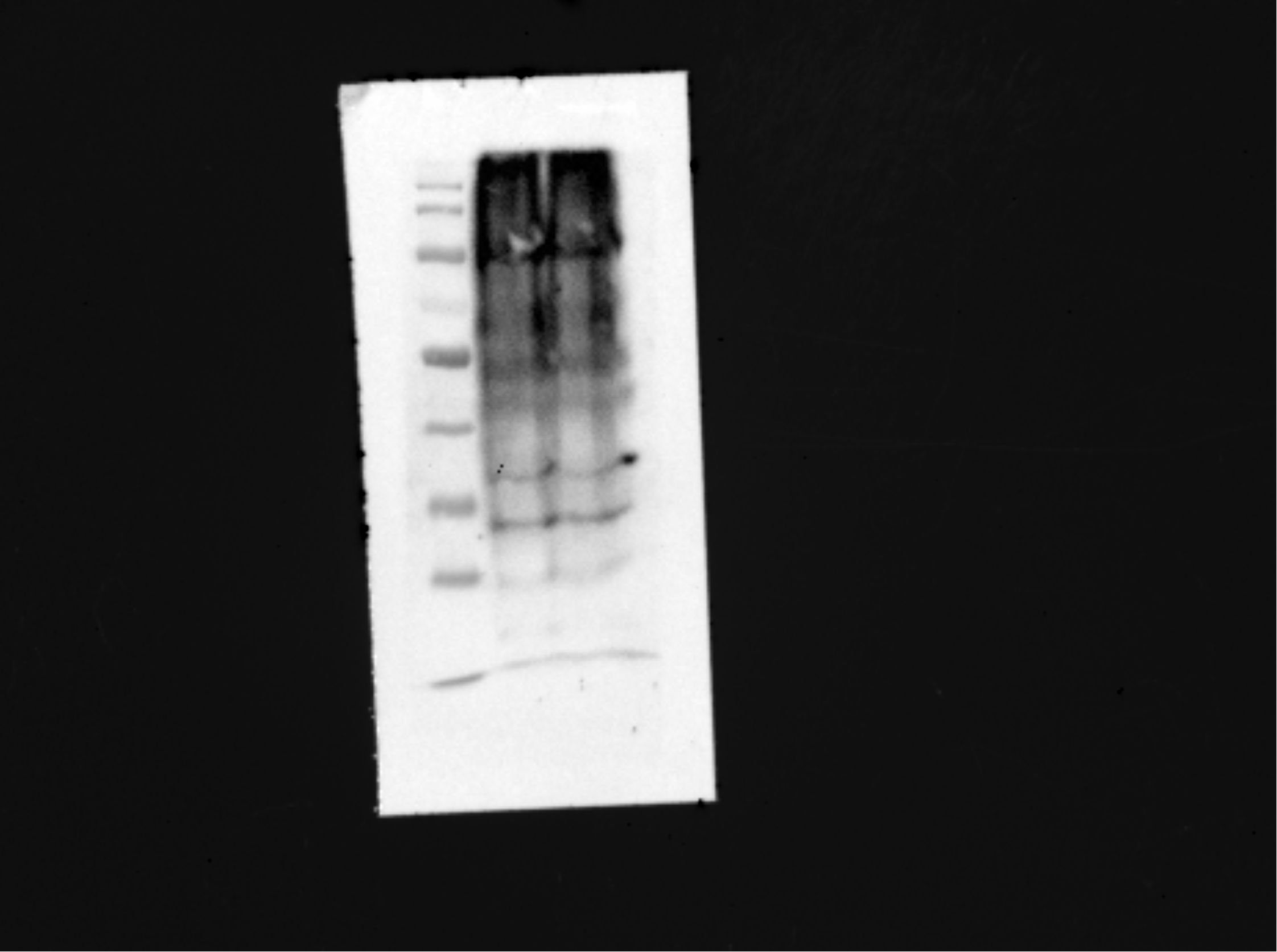


Full and uncropped western blots for figure S7D-1-2


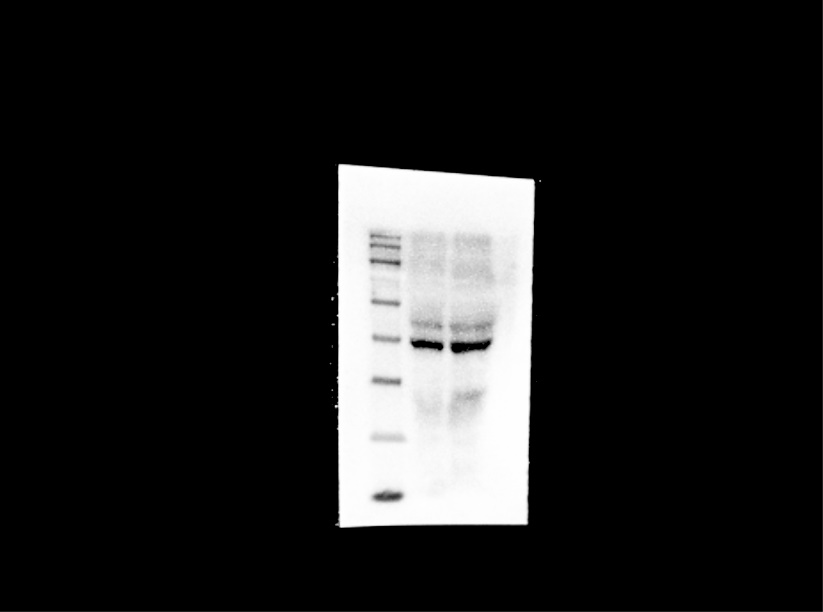


Full and uncropped western blots for figure S7D-2-1


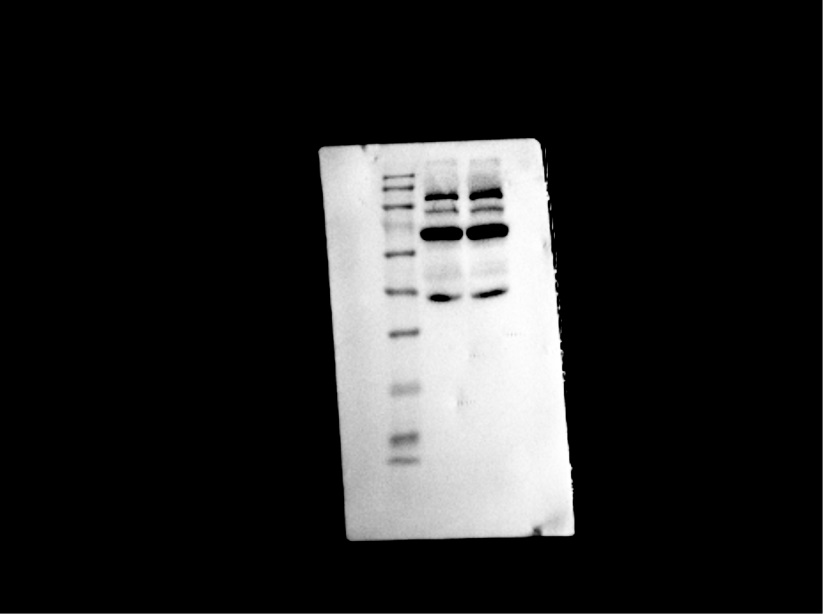


Full and uncropped western blots for figure S7D-2-2


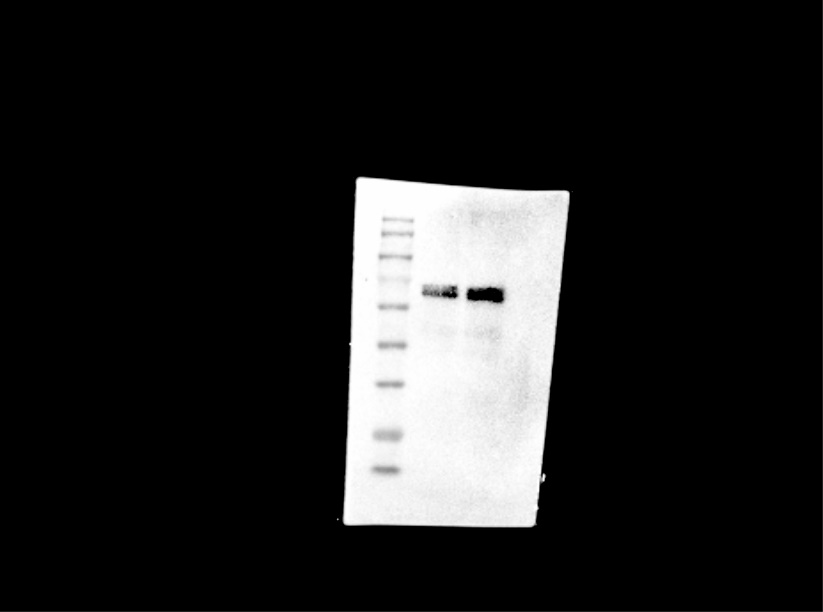


Full and uncropped western blots for figure S7D-3-1


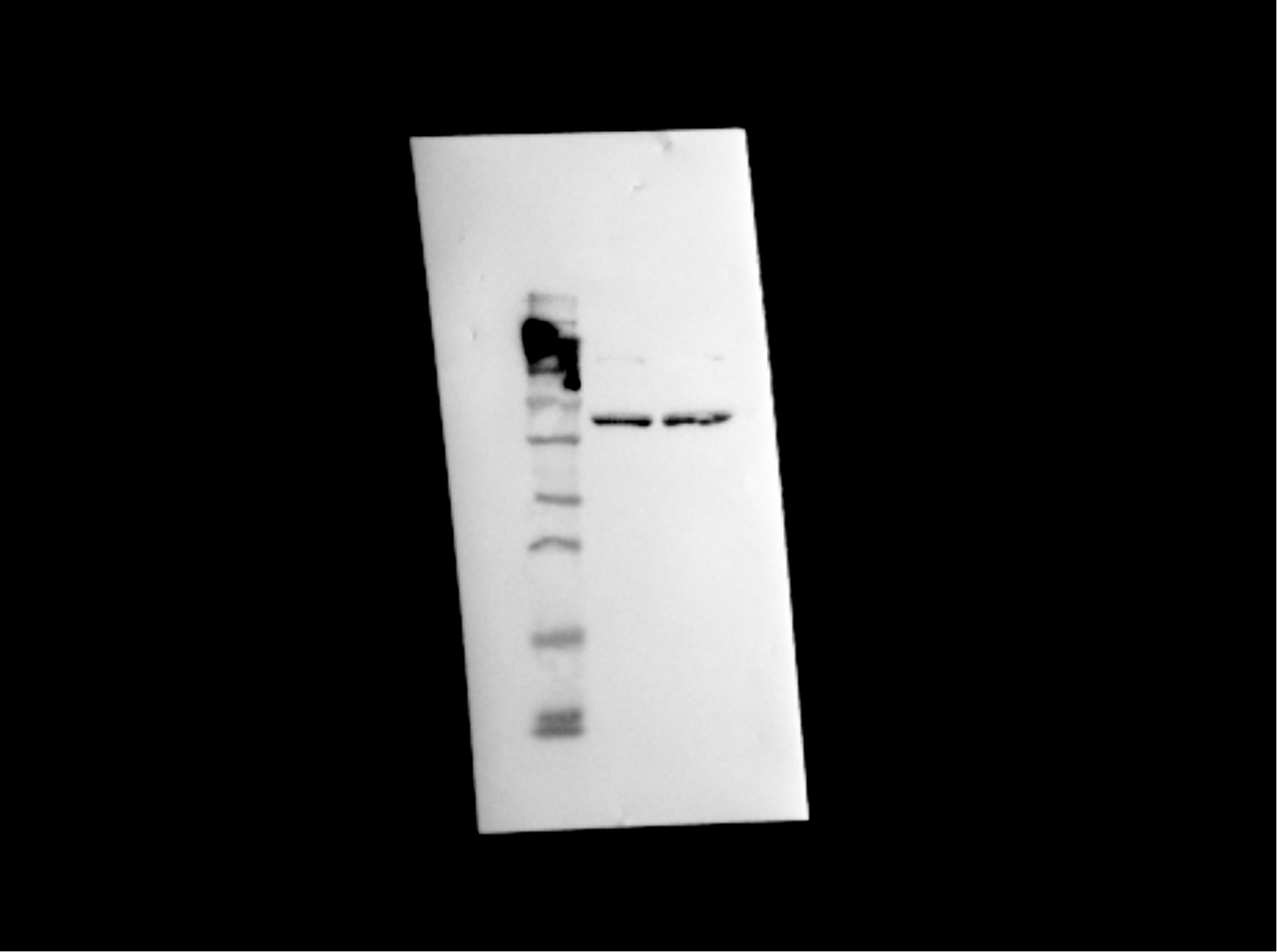


Full and uncropped western blots for figure S7D-3-2


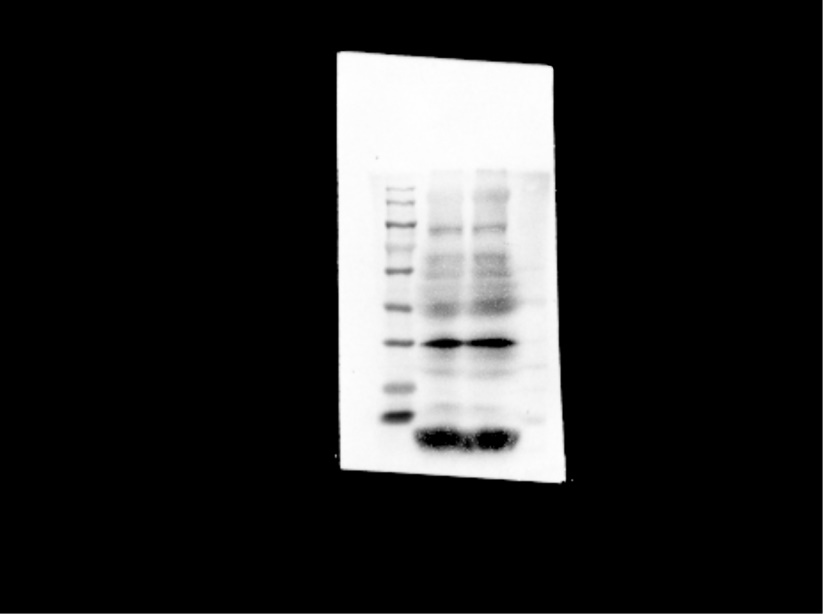


Full and uncropped western blots for figure S7D-4-1

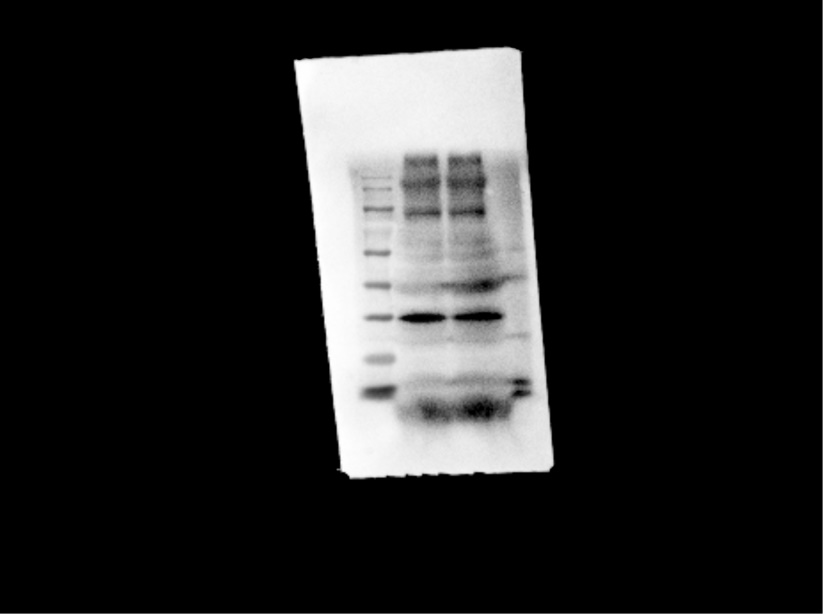


Full and uncropped western blots for figure S7D-4-2


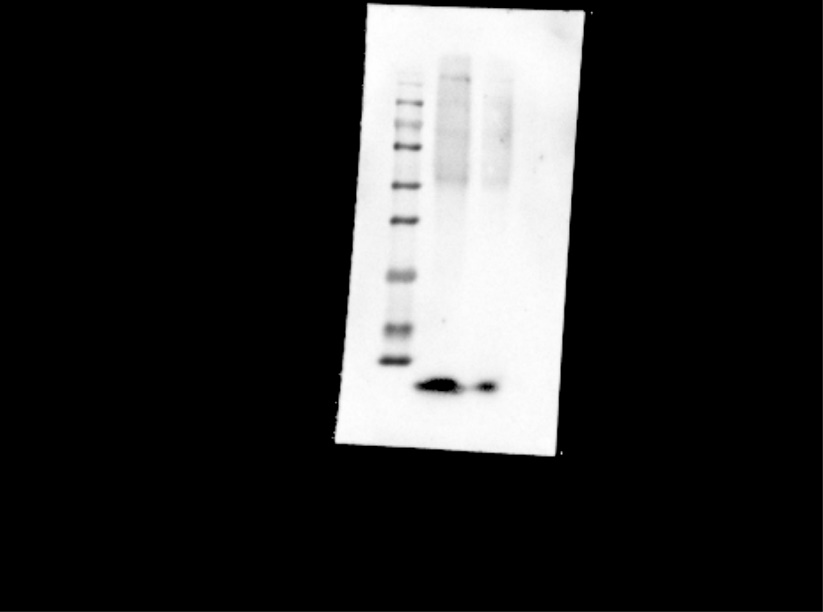


Full and uncropped western blots for figure S8A-1-1


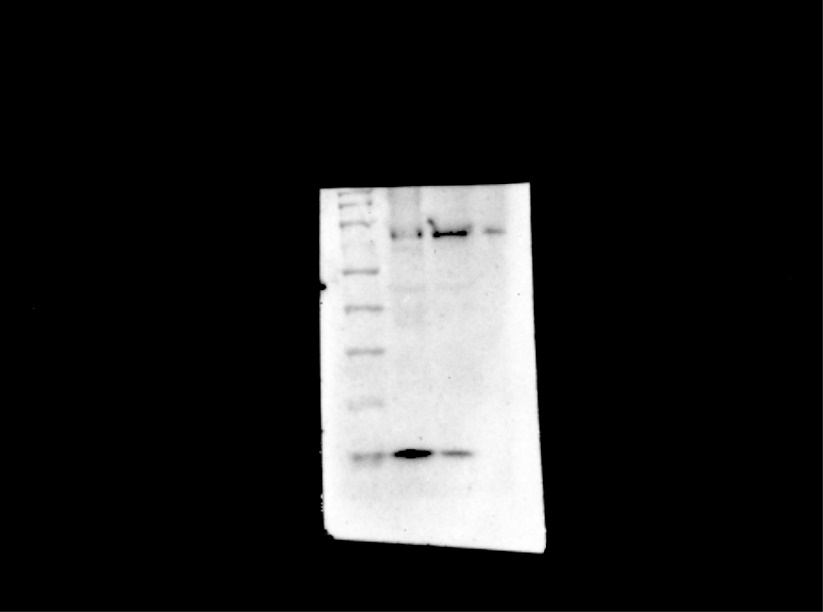


Full and uncropped western blots for figure S8A-1-2


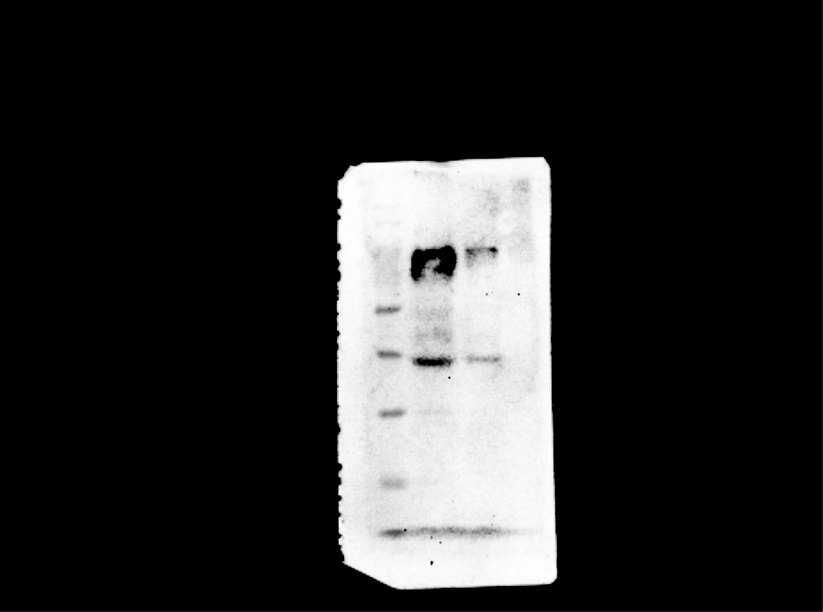


Full and uncropped western blots for figure S8A-2-1


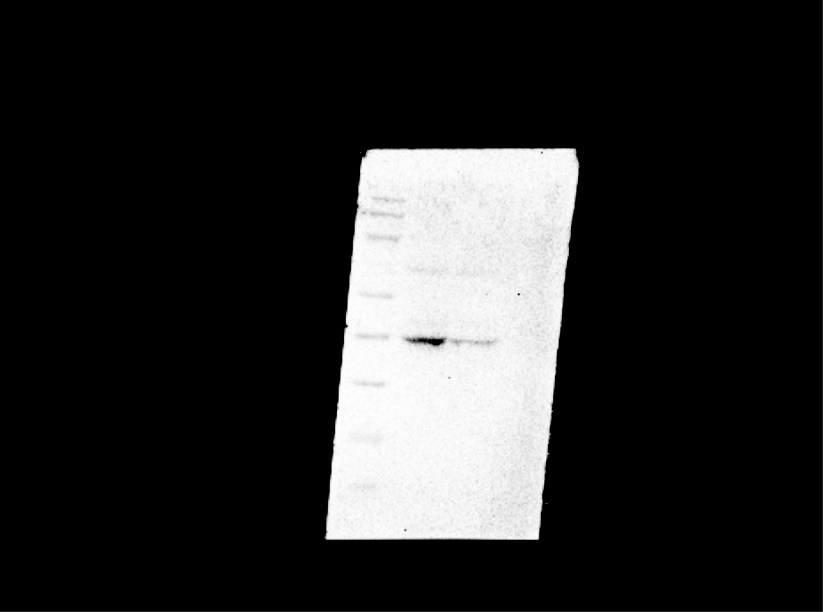


Full and uncropped western blots for figure S8A-2-2


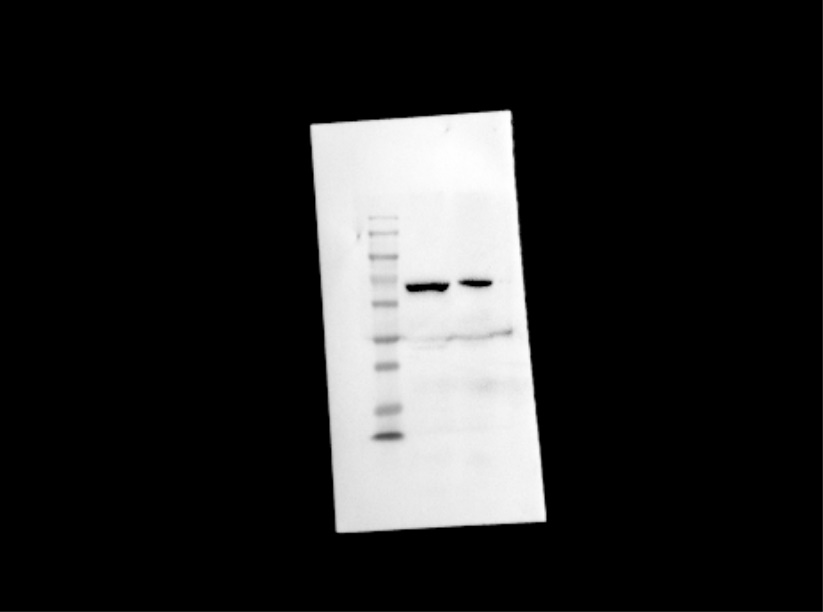


Full and uncropped western blots for figure S8A-3-1


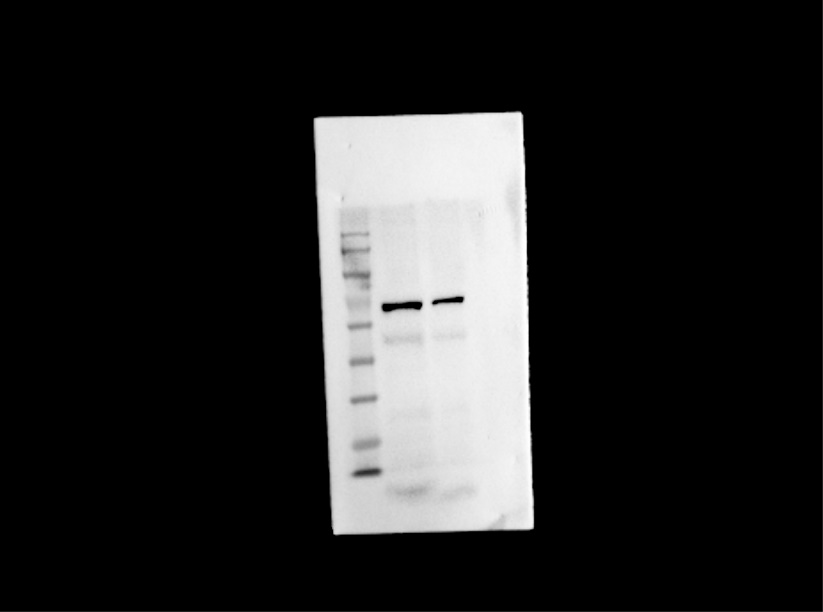


Full and uncropped western blots for figure S8A-3-2


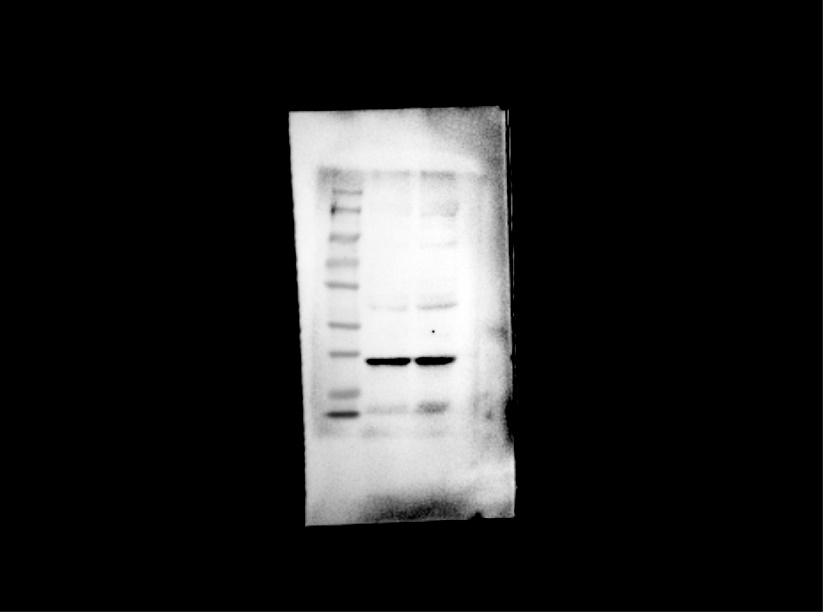


Full and uncropped western blots for figure S8A-4-1


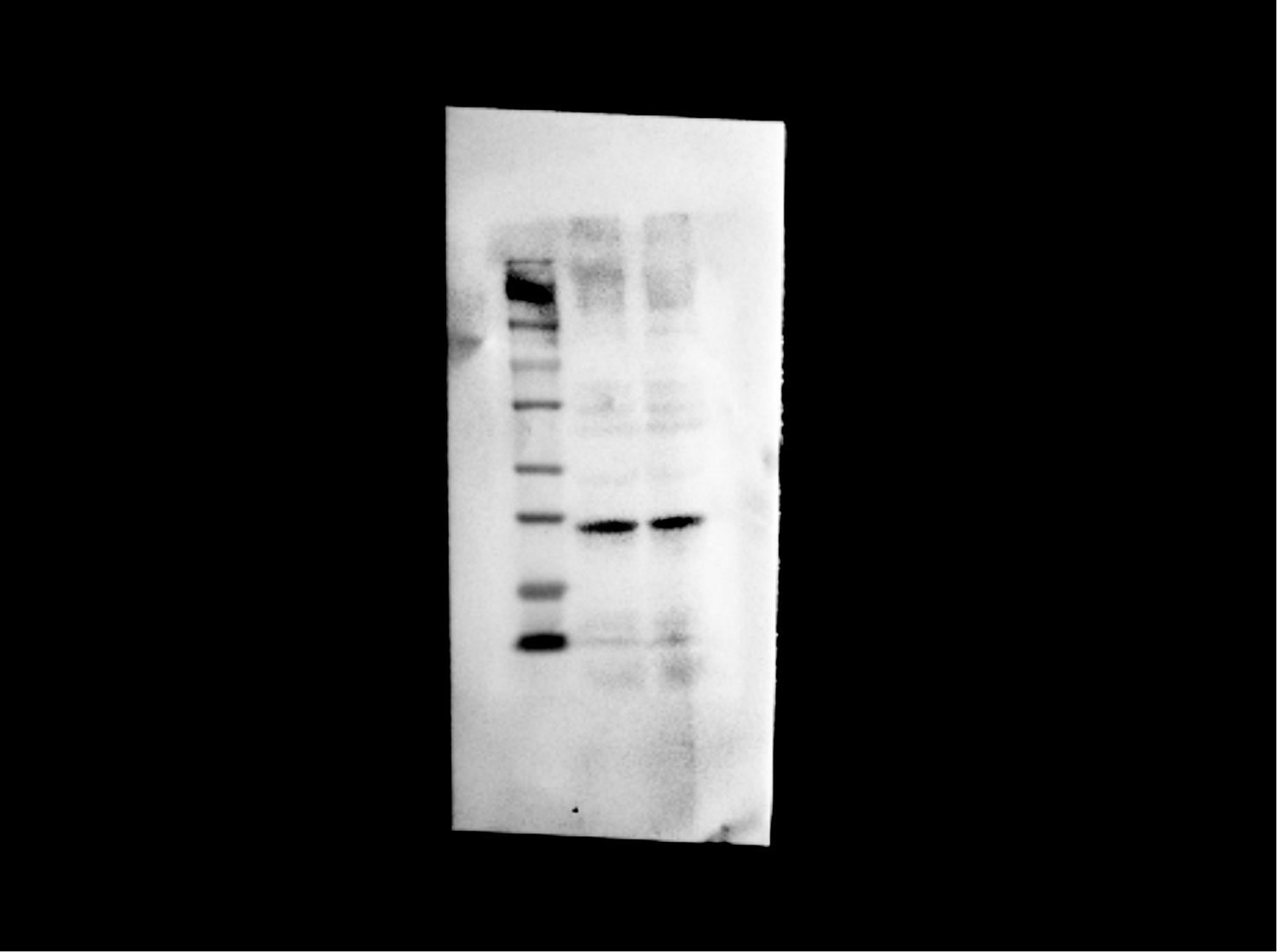


Full and uncropped western blots for figure S8A-4-2


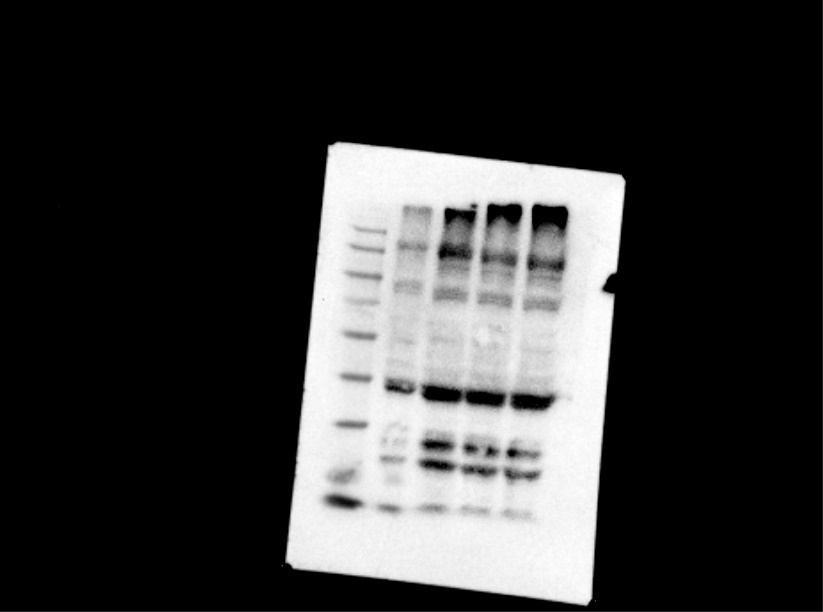


Full and uncropped western blots for figure S9A-1


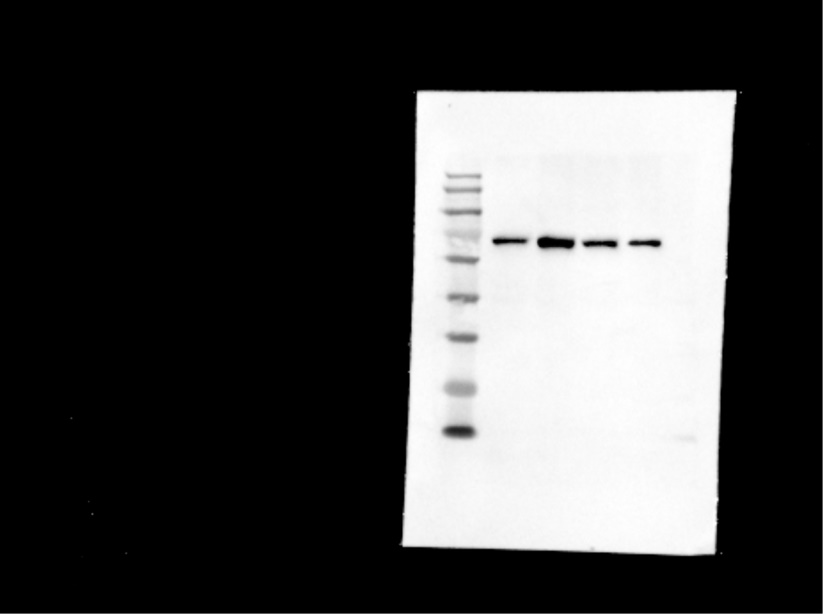


Full and uncropped western blots for figure S9A-2


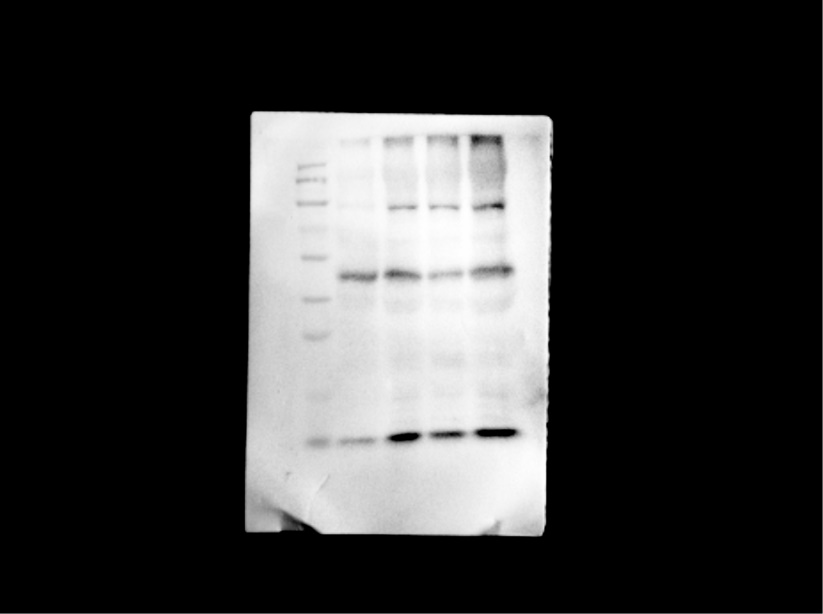


Full and uncropped western blots for figure S9A-3


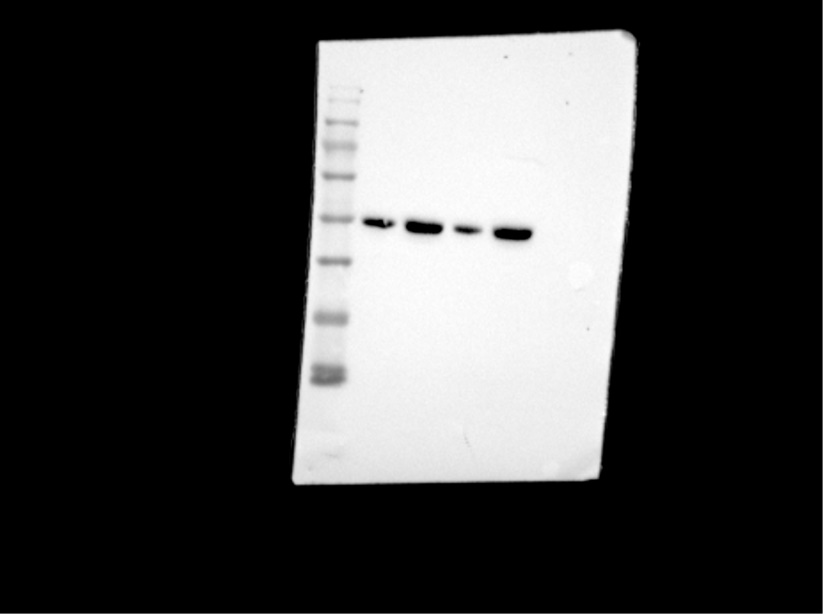


Full and uncropped western blots for figure S9A-4


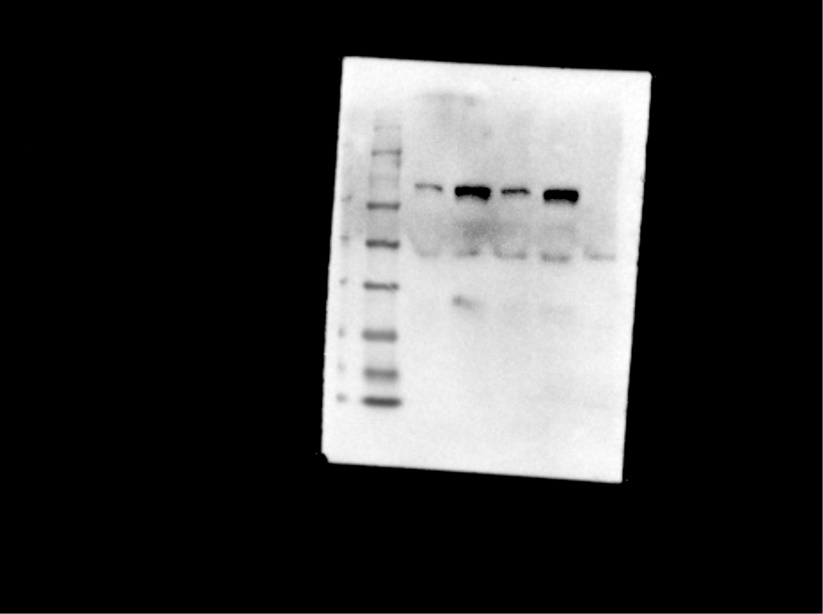


Full and uncropped western blots for figure S9A-5


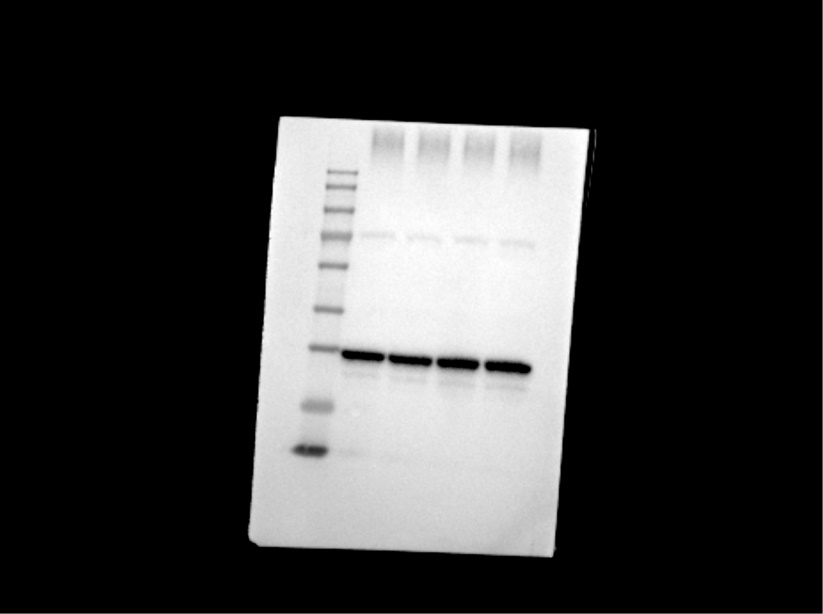


Full and uncropped western blots for figure S9A-6
