# Supplementary material for: Single-cell multi-omics elucidates the role of RPS27-RPS24 fusion gene in osteosarcoma chemoresistance and metabolic regulation
Source: Cell Death Discov. 2025 Apr 25;11:197. doi: 10.1038/s41420-025-02487-9 (PMC12032165; doi:10.1038/s41420-025-02487-9)
Supplement: Supplementary file 2 — Supplementary Materials [file 41420_2025_2487_MOESM2_ESM.docx]

**Table S1. Public dataset used in this study**

| **Dataset** | **Source** | **Type** | **Sample** | **Use** |
| --- | --- | --- | --- | --- |
| GSE162454 | GEO | scRNA-seq | 6 samples of tumor tissue from OS patients | UMAP clustering analysis, cell annotation, and copy number variation analysis |
| GSE154540 | GEO | RNA-seq | 29 samples of tumor tissue from chemotherapy-sensitive and 21 samples from chemoresistance OS patients | Weighted gene co-expression network analysis (WGCNA) |
| GSE39055 | GEO | RNA-seq | 37 samples of tumor tissue from OS patients | Clinical correlation analysis (necrosis rate and tumor recurrence) |
| TARGET-OS | TARGET | RNA-seq | 96 samples of tumor tissue from OS patients | Survival analysis, correlation analysis. |

**Table S2. RT-qPCR primer sequences**

| **Name** | **Sequences** |
| --- | --- |
| GLS (human) | F: 5'- TGGAAAAGAGCCGAGTGGAC -3' |
|  | R: 5'- AAGGAATGCCTTTGATCACCAC -3' |
| GAPDH (human) | F: 5'- AATGGGCAGCCGTTAGGAAA -3' |
|  | R: 5'- GCGCCCAATACGACCAAATC -3' |

**Table S3. Western blot antibody information**

| **Gene** | **Cat.** | **Dilution ratio** | **Manufacturer** | **Country** | **MW (kDa)** |
| --- | --- | --- | --- | --- | --- |
| RPS27 | ab197382 | 1: 200 | abcam | UK | 12 |
| RPS24 | ab196652 | 1: 1000 | abcam | UK | 15 |
| GLS | ab156876 | 1: 2000 | abcam | UK | 73 |
| FDX1 | ab108257 | 1: 2000 | abcam | UK | 14 |
| LIAS | ab96302 | 1: 500 | abcam | UK | 42 |
| DLAT | ab172617 | 1: 1000 | abcam | UK | 70 |
| GAPDH | ab9485 | 1: 2500 | abcam | UK | 37 |
| Flag-DDDDK | ab205606 | 1: 500 | abcam | UK | / |

**
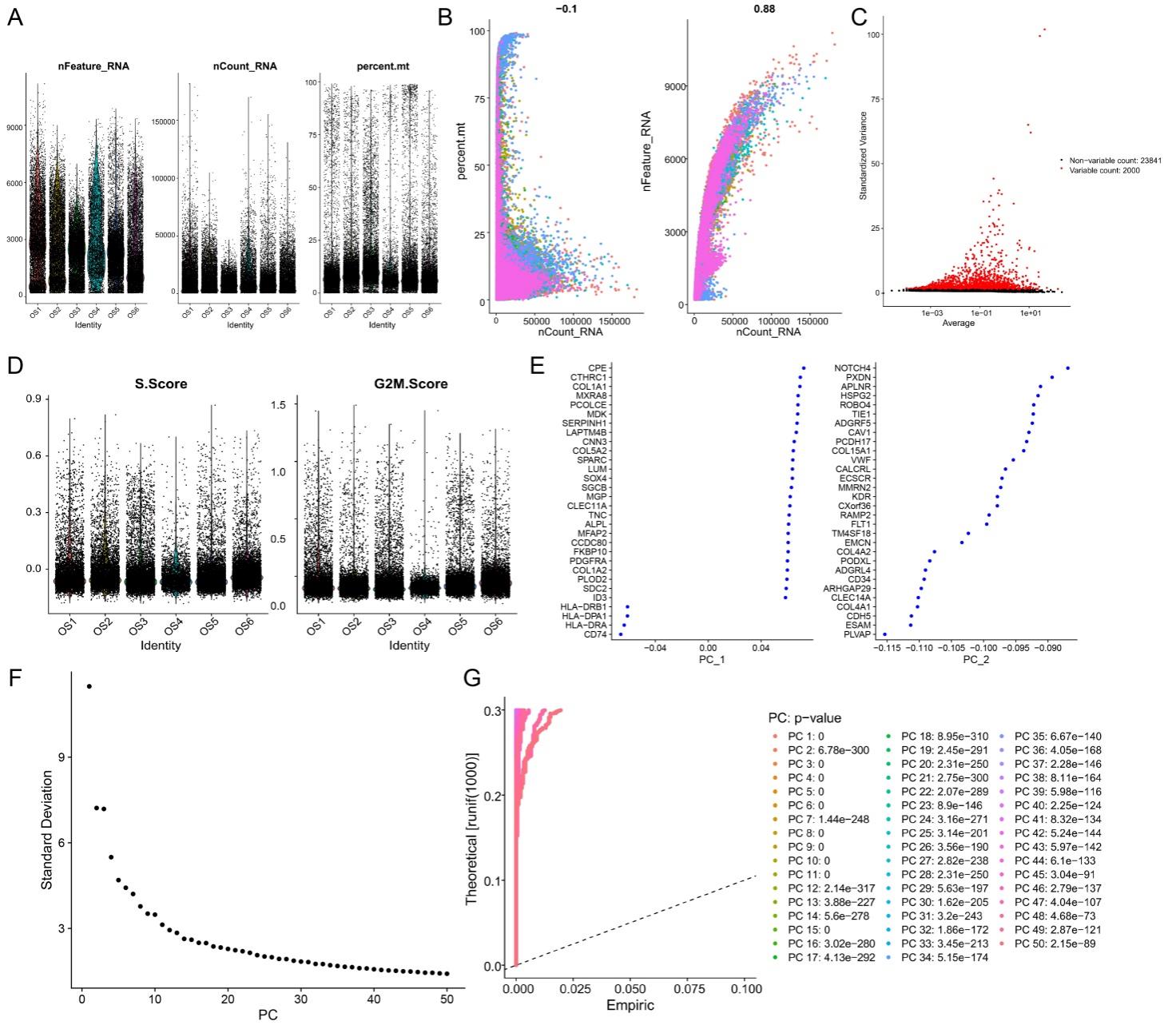
**

**Figure S1. Data quality control, variance analysis, and PCA of scRNA-seq data.**

Note: (A) Violin plots (N = 6) showing gene count (nFeature_RNA), mRNA molecule count (nCount_RNA), and percentage of mitochondrial genes (percent.mt) for each cell in scRNA-seq data; (B) Correlation between nCount and percent.mt (left) and between nCount and nFeature (right) (N = 6); (C) Variance analysis to identify highly variable expressed genes, with red representing the top 2000 highly variable genes and black representing low variable genes; (D) Cell cycle states of each cell, with S.Score representing the S phase and G2M.Score representing the G2M phase (N = 6); (E) Scatter plot of top 30 gene expression points for PC_1 and PC_2 in PCA analysis (N = 6); (F) Distribution of standard deviations of principal components, with larger standard deviations indicating more "important" principal components (N = 6); (G) Distribution of *P* for principal components relative to the mean distribution, with smaller p-values indicating more "important" principal components, dashed line represents *P* = 0.05, and values above the dashed line indicate *P* < 0.05.

**
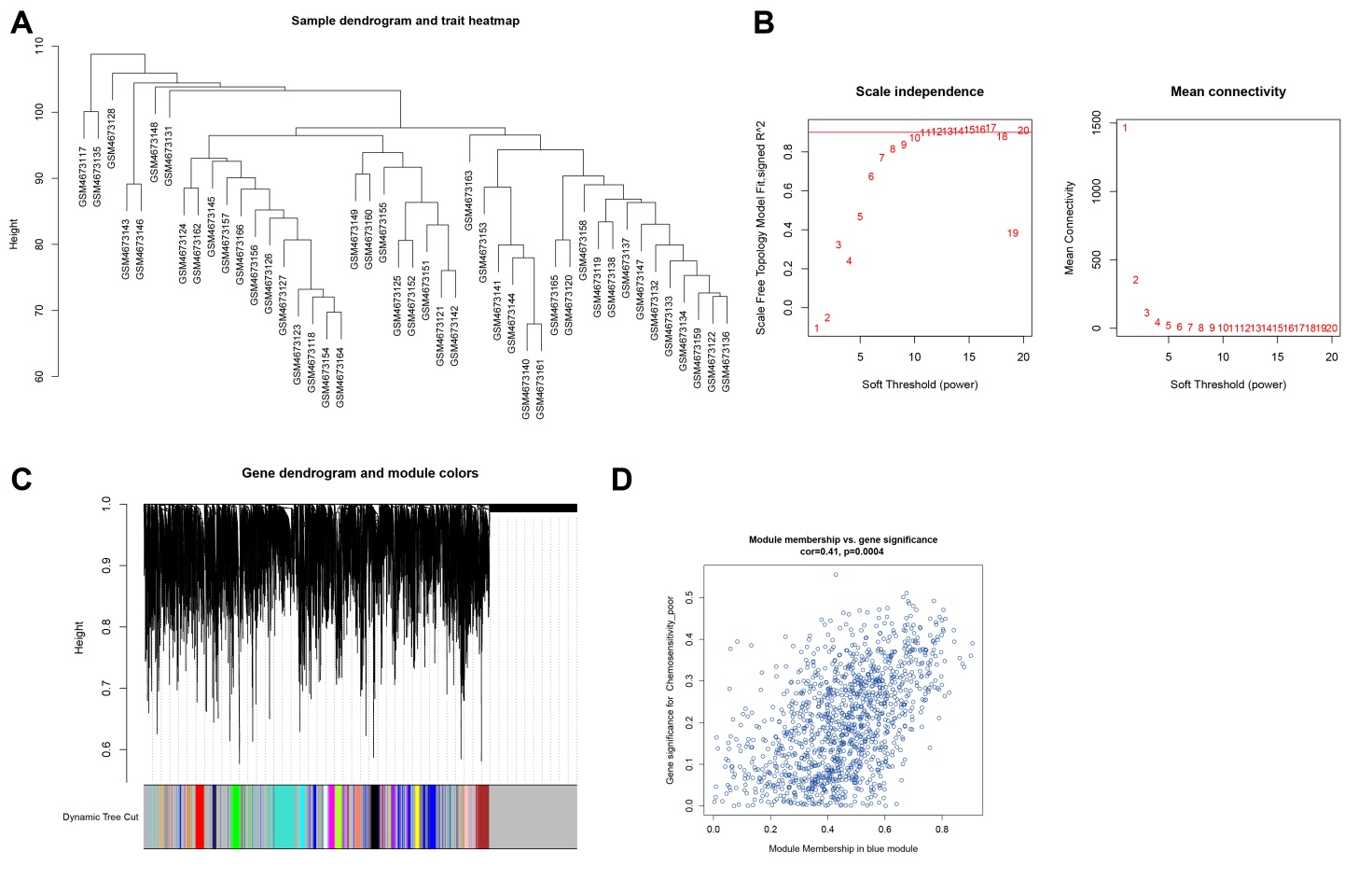
**

**Figure S2. Identification of key modules in chemoresistance in OS using WGCNA.**

Note: (A) Sample clustering dendrogram of the merged dataset (N = 50); (B) Scale-free fit index (left) and average connectivity (right) for various soft-thresholding powers β, with the red line indicating a correlation coefficient (R^2^=0.9); (C) Setting a mergeCutHeight of 0.25 resulted in the division into 18 modules, with each color representing a module; (D) Scatter plot showing the correlation between the blue module characteristic genes and clinical features.

**
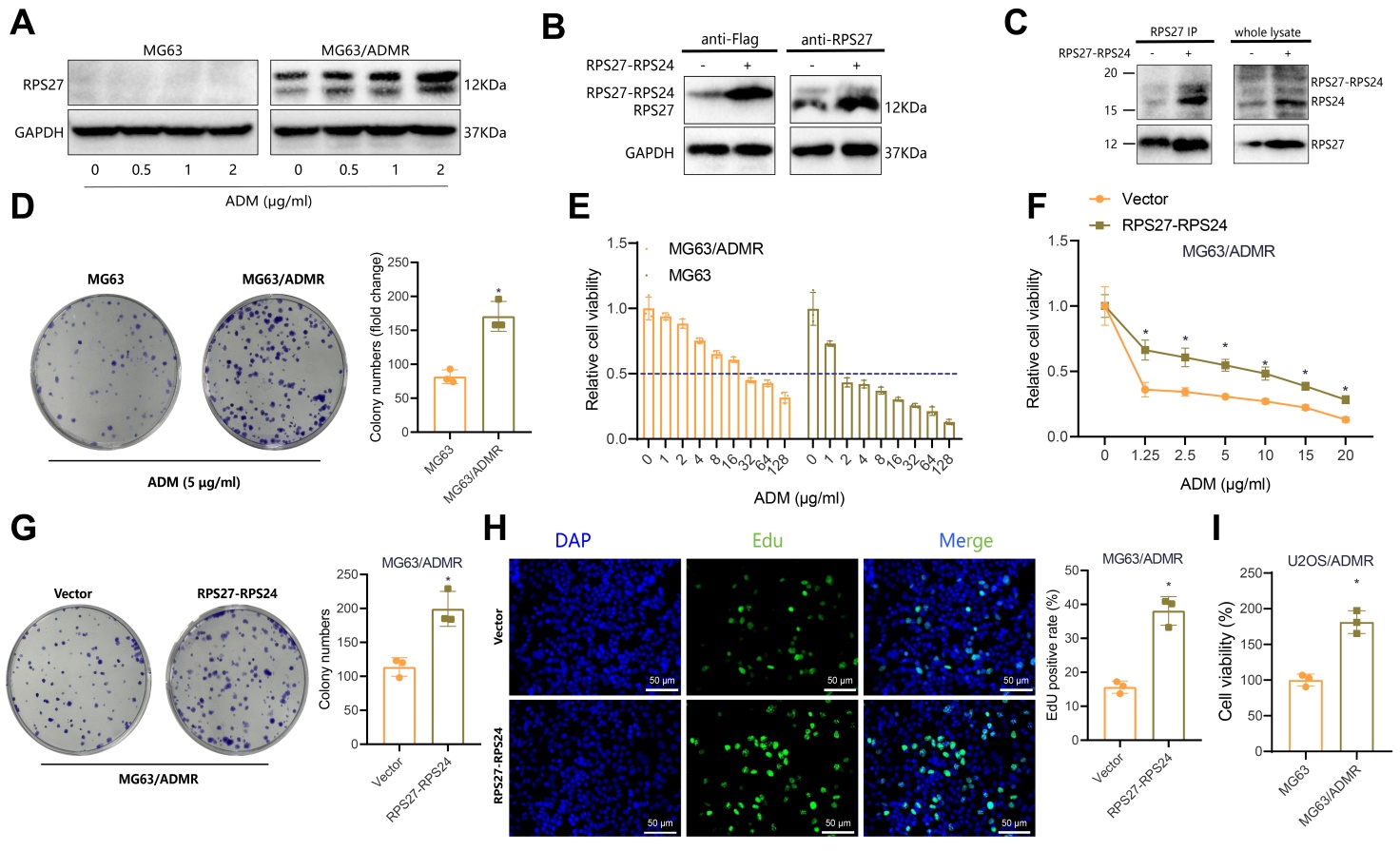
**

**Figure S3. Impact of RPS27-RPS24 on chemoresistance in MG63 cells.**

Note: (A) Western blot analysis of RPS27 protein expression in MG63 and MG63/ADMR cells in different groups; (B) Western blot analysis of RPS27 protein expression in MG63 cells transfected with either Vector or RPS27-RPS24 overexpression plasmids; (C) IP experiment detecting the expression of RPS27-RPS24 protein in MG63 cells transfected with either Vector or RPS27-RPS24 overexpression plasmids; (D) Clonogenic assay measuring cell proliferation of MG63 and MG63/ADMR cells after treatment with 5 µg/ml ADM, along with the corresponding statistical graph; (E) Sensitivity of MG63 and MG63/ADMR cells to ADM; (F) MTT assay evaluating cell viability of MG63/ADMR cells treated with different concentrations of ADM for 48 hours; (G) Clonogenic assay measuring cell proliferation of MG63/ADMR cells treated with 5 µg/ml ADM after transfection with either Vector or RPS27-RPS24 overexpression plasmids, along with the corresponding statistical graph; (H) EdU assay assessing cell proliferation of MG63/ADMR cells treated with 5 µg/ml ADM after transfection with either Vector or RPS27-RPS24 overexpression plasmids (scale bar: 50 μm), along with the corresponding statistical graph; (I) Treatment of MG63/ADMR cells with 1 µM Thapsigargin for 24 hours and performance of MTT assays post-transfection with either Vector or RPS27-RPS24 overexpression plasmids. An independent sample t-test was applied for comparing two groups, and two-way ANOVA was used for time-based data comparisons. * indicates *P* < 0.05 compared to the MG63 group or Vector group. Cell experiments were performed in triplicates.


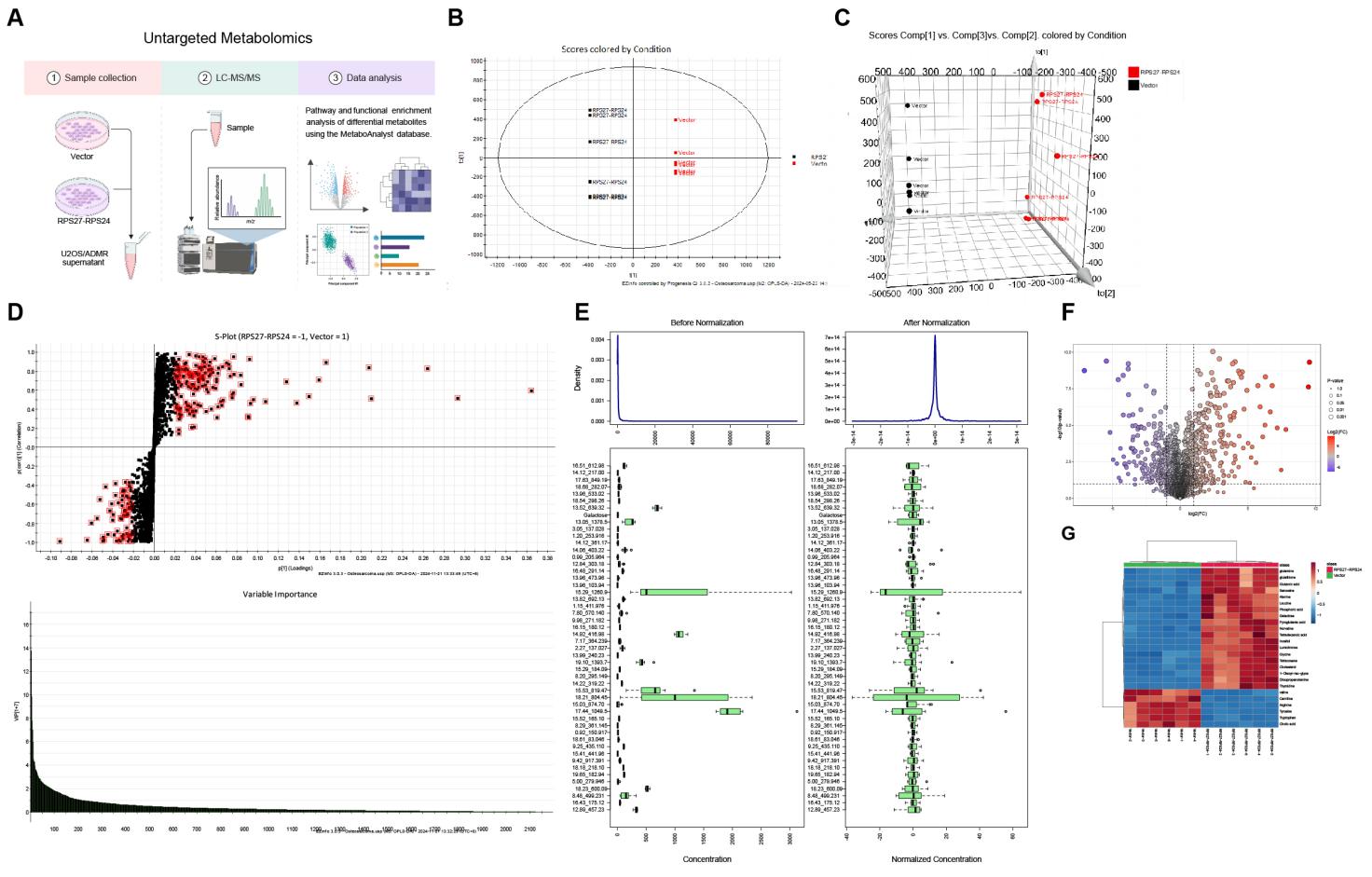


**Figure S4. Non-targeted Metabolomics Analysis of U2OS/ADMR Cells with Stable Expression of Vector or RPS27-RPS24.**

(A) Schematic diagram of the metabolomics workflow in this study; (B) 2D PCA plot of data from Vector group (N=6) and RPS27-RPS24 group (N=6) U2OS/ADMR cell samples; (C) 3D PCA plot of data from Vector group (N=6) and RPS27-RPS24 group (N=6) U2OS/ADMR cell samples; (D) Differential metabolite screening, with red box indicating VIP > 1; (E) Normalized intensities before (left) and after (right) data correction; (F) Volcano plot analysis of differences between the Vector group and RPS27-RPS24 group, with red representing upregulated metabolites, blue representing downregulated metabolites, and gray indicating metabolites with insignificant differential expression; (G) Heatmap of the top 25 metabolites based on VIP value.

**
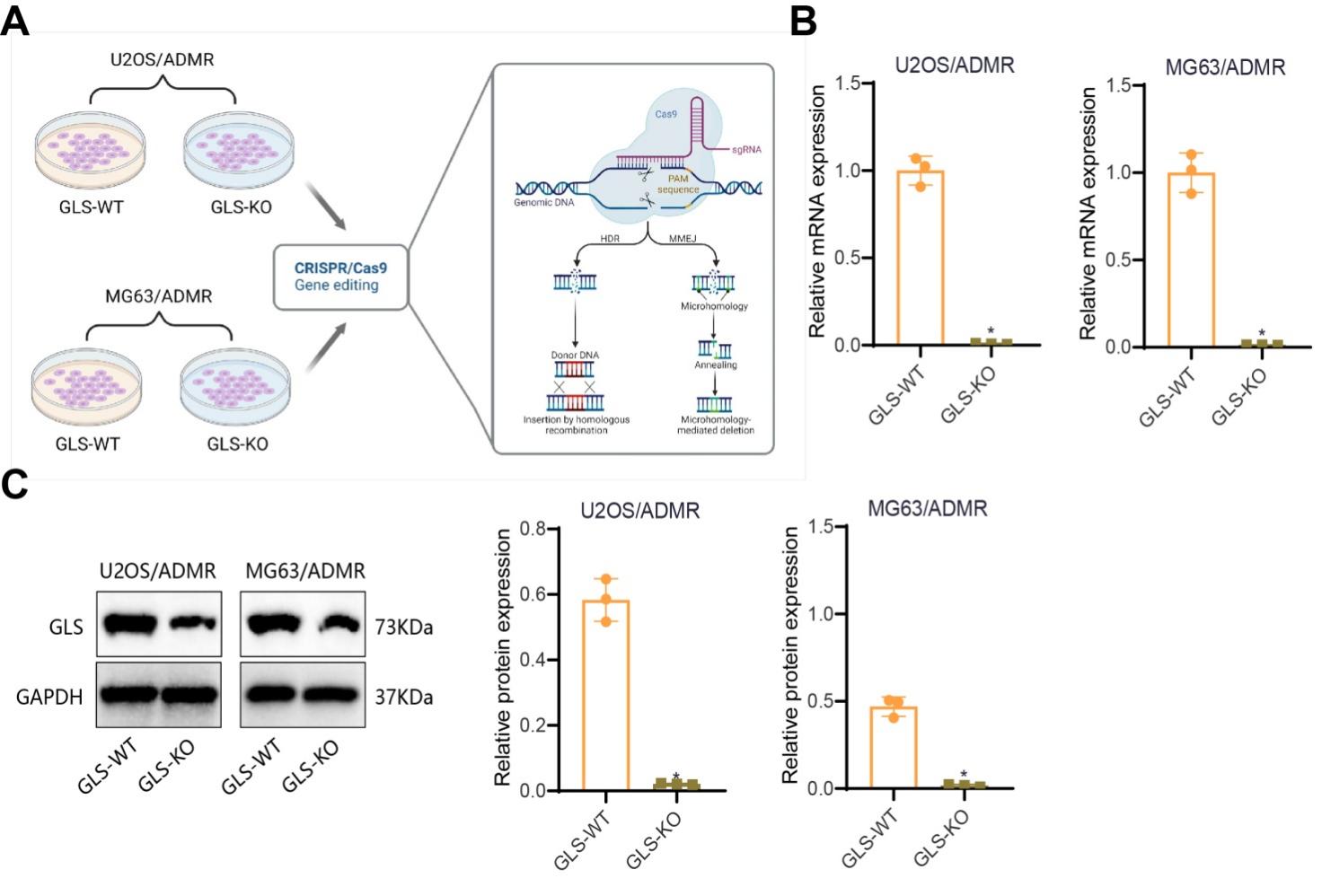
**

**Figure S5. GLS knockout using CRISPR/Cas9 technology.**

Note: (A) Schematic representation of the CRISPR/Cas9 technology workflow; (B-C) Validation of GLS knockout efficiency through RT-qPCR (B) and Western blot (C); * indicates *P* < 0.05 compared to the GLS-WT group. Cell experiments were repeated three times.

**
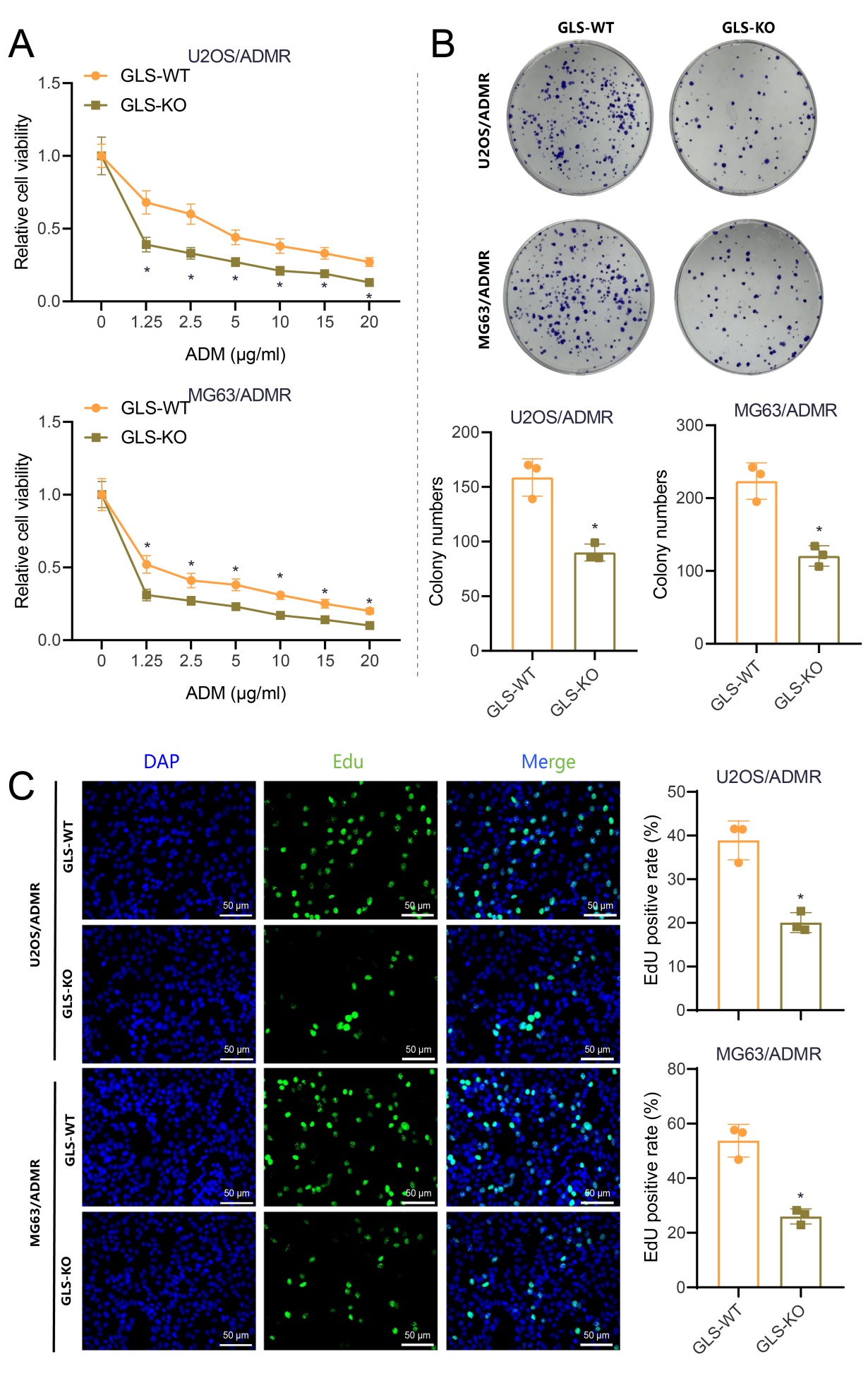
**

**Figure S6. Effects of GLS knockdown on chemoresistance in U2OS/ADMR and MG63/ADMR cells.**

Note: (A) Cell viability was assessed by MTT assay after treating OS cells with different concentrations of ADM for 48 h; (B) Clonogenic assay was performed to assess cell proliferation after transfection with Vector or RPS27-RPS24 overexpression plasmids, and the results were represented in a statistical graph; (C) Cell proliferation was evaluated using the EdU assay after transfection with Vector or RPS27-RPS24 overexpression plasmids (scale bar: 50 μm), and the results were represented in a statistical graph. Independent samples t-tests were employed to compare the two sets of data, and two-way ANOVA was conducted to compare the data across different time points. * indicates *P* < 0.05 compared to the GLS-WT or Vector group; all cell experiments were performed thrice.

**
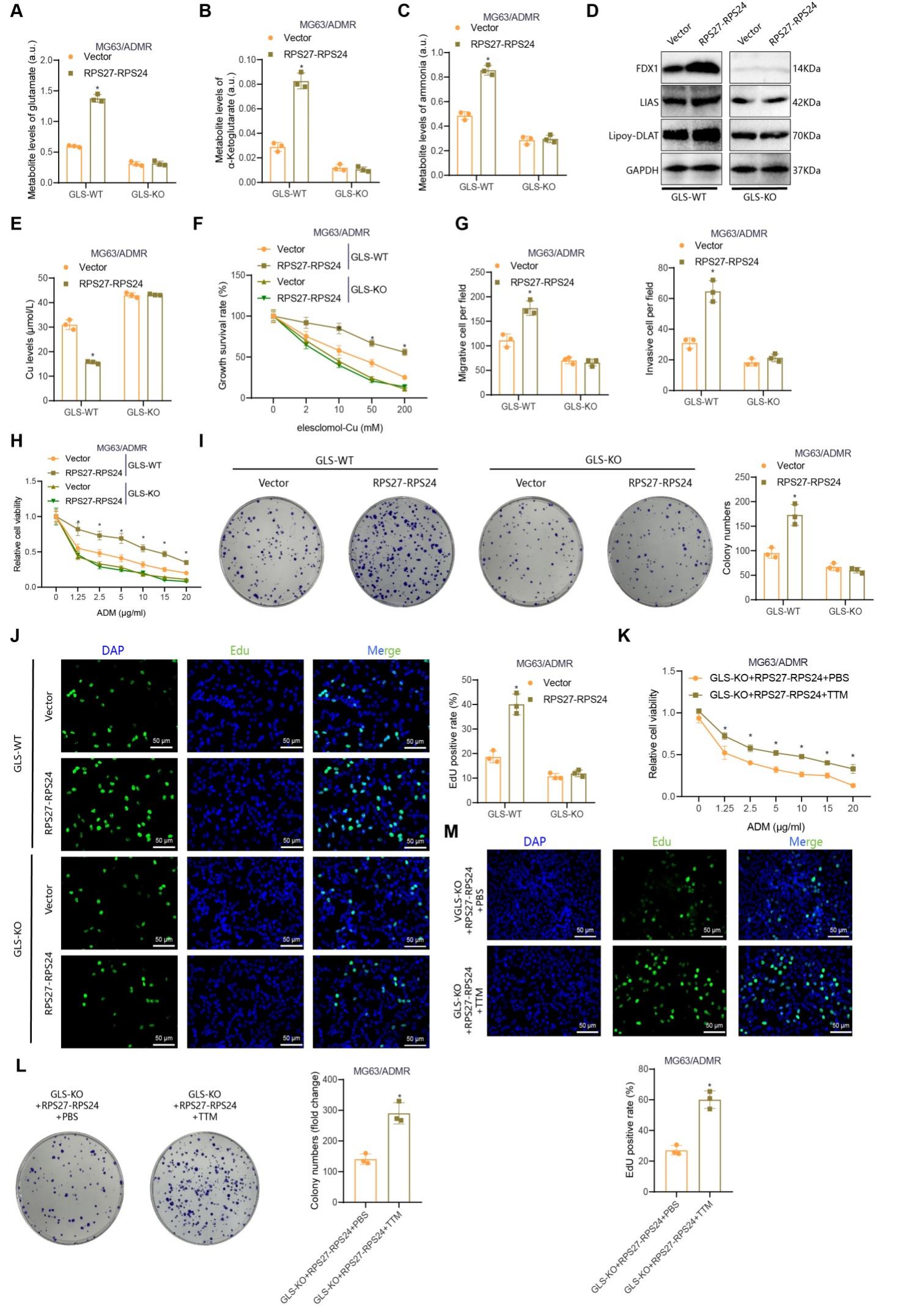
**

**Figure S7. Effects of RPS27-RPS24 on GLS-mediated glutaminolysis, copper death, and chemoresistance in MG63/ADMR cells.**

Note: (A-C) The intracellular levels of glutamine (A), α-ketoglutarate (B), and ammonia (C) were measured after adding glutamine medium for 8 h in each group; (D) Protein expression of FDX1, LIAS, and Lipoy-DLAT was detected by Western blot; (E) Copper ion levels in the cells were determined; (F) Cell survival and growth were assessed by CCK-8 assay after treating the cells with different concentrations of elesclomol-Cu (ratio=1:1) for 72 h; (G) Cell migration capacity was evaluated by scratch assay (upper panel) and cell invasion ability was assessed using the Transwell assay (lower panel); (H) Cell viability of GLS-WT and GLS-KO MG63/ADMR cells was measured by MTT assay; (I) Clonogenic assay was performed to assess cell proliferation of GLS-WT and GLS-KO cells after treatment with 5 µg/ml ADM, and the results were represented in a statistical graph; (J) Cell proliferation was evaluated using the EdU assay after treatment with 5 µg/ml ADM in GLS-WT and GLS-KO cells (scale bar: 50 μm), and the results were represented in a statistical graph; (K) Cell viability of GLS-KO+RPS27-RPS24+PBS and GLS-KO+RPS27-RPS24+TTM MG63/ADMR cells was measured by MTT assay; (L) Clonogenic assay was performed to assess cell proliferation of GLS-KO+RPS27-RPS24+PBS and GLS-KO+RPS27-RPS24+TTM cells after treatment with 5 µg/ml ADM, and the results were represented in a statistical graph; (M) Cell proliferation was evaluated using the EdU assay after treatment with 5 µg/ml ADM in GLS-KO+RPS27-RPS24+PBS and GLS-KO+RPS27-RPS24+TTM cells (scale bar: 50 μm), and the results were represented in a statistical graph. When comparing two sets of data, an independent samples t-test is employed. For comparing data across different time periods or factors, a two-way ANOVA was utilized. * indicates *P* < 0.05 compared to the Vector or GLS-KO+RPS27-RPS24+PBS group; all cell experiments were performed thrice.

**
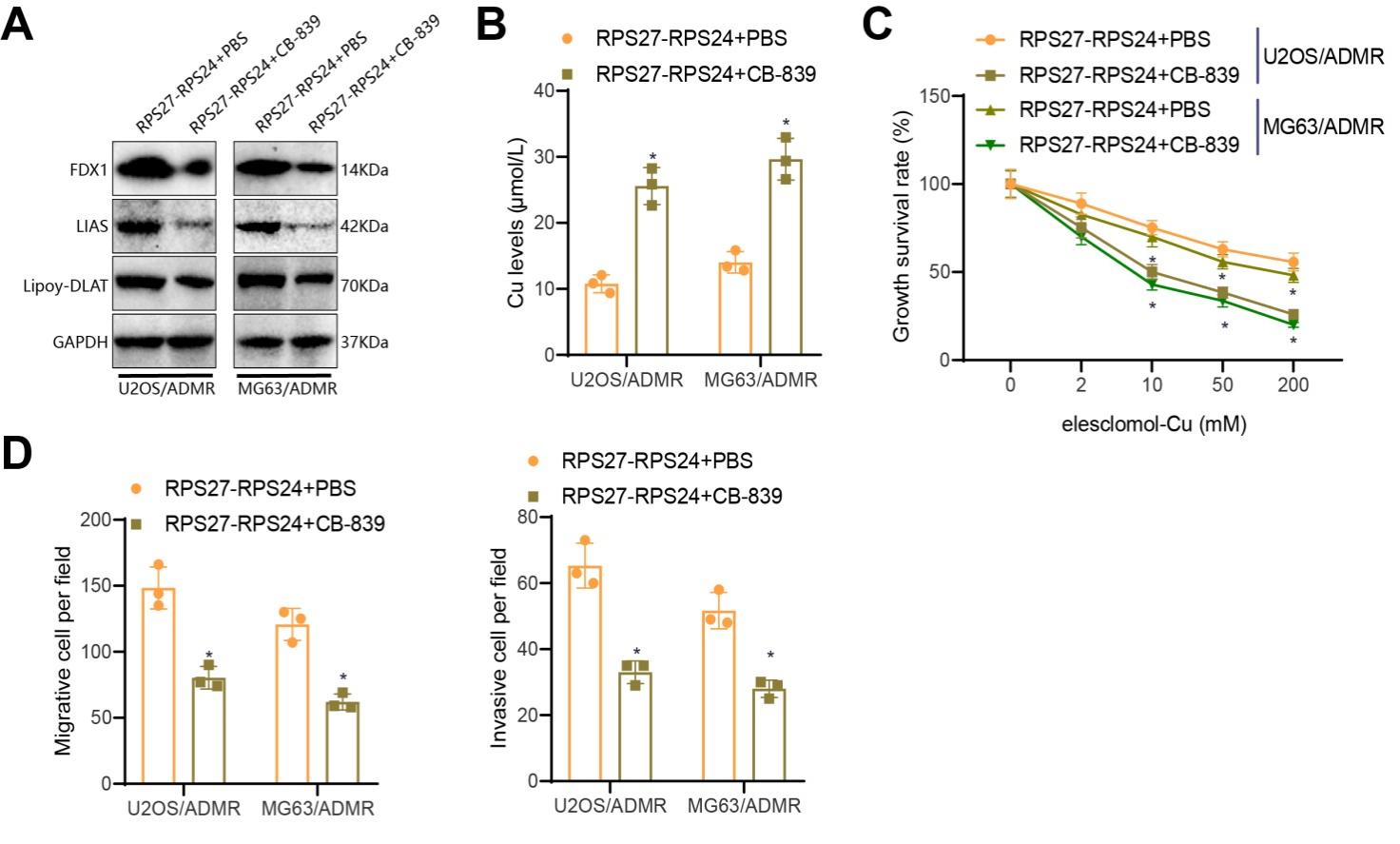
**

**Figure S8. Effects of the glutaminolysis inhibitor CB-839 on copper death in U2OS/ADMR and MG63/ADMR cells.**

Note: (A) Protein expression of FDX1, LIAS, and Lipoy-DLAT was detected by Western blot; (B) Copper ion levels in the cells were determined; (C) Cell survival and growth were assessed by CCK-8 assay after treating the cells with different concentrations of elesclomol-Cu (ratio=1:1) for 72 h; (D) Cell migration capacity was evaluated by scratch assay (upper panel) and cell invasion ability was assessed using the Transwell assay (lower panel). For comparing data between two groups, an independent samples t-test was conducted. To compare data across different time points, a two-way ANOVA was employed. * indicates *P* < 0.05 compared to the RPS27-RPS24+PBS group; all cell experiments were performed thrice.

**
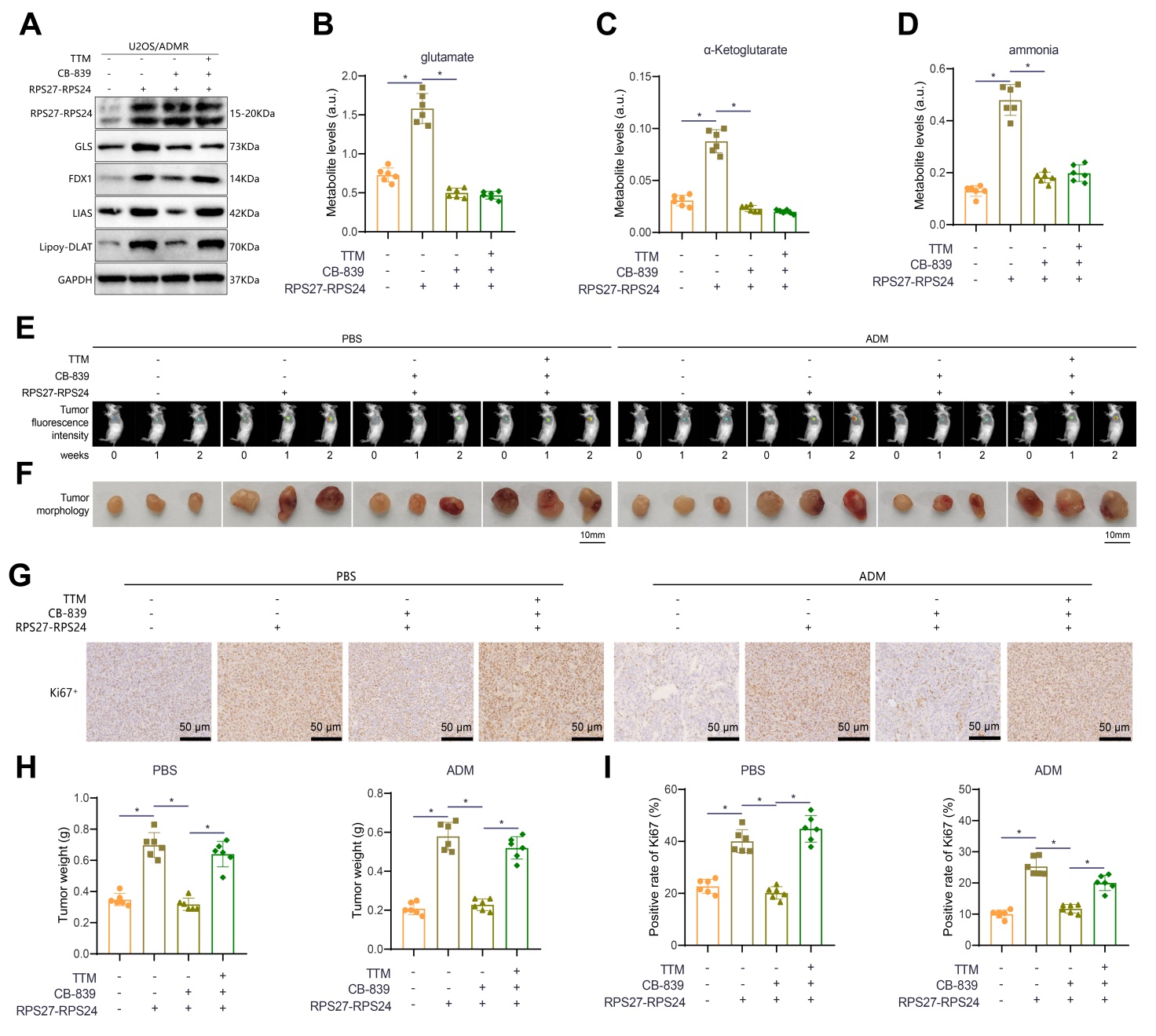
**

**Figure S9. Effects of RPS27-RPS24 on tumorigenesis and chemoresistance in MG63 cells through regulation of glutamine metabolism and copper death.**

Note: (A) Protein expression levels of RPS27-RPS24, GLS, FDX1, LIAS, and Lipoy-DLAT in tumor tissues of different groups were detected by Western blot; (B-D) Metabolism levels of glutamine (B), α-ketoglutarate (C), and ammonia (D) in tumor tissues of different groups were assessed; (E) Tumor growth was monitored at different time points using bioluminescence intensity; (F) Morphology of tumor tissues from different groups, with 3 representative examples shown for each group; (G) Immunohistochemical staining was performed to detect the protein expression level of Ki67 in tumor tissues of different groups (scale bar = 50 μm); (H) Tumor tissue weight in different groups; (I) Statistical analysis of Ki67 positive expression. For comparing data between different groups, one-way ANOVA was utilized. * indicates *P* < 0.05 compared to the Vector+PBS+PBS group or RPS27-RPS24+PBS+PBS group or RPS27-RPS24+CB-839+PBS group ), with 6 mice in each group.
